# HuR/ELAVL1 drives malignant peripheral nerve sheath tumor growth and metastasis

**DOI:** 10.1172/JCI130379

**Published:** 2020-06-15

**Authors:** Marta Palomo-Irigoyen, Encarni Pérez-Andrés, Marta Iruarrizaga-Lejarreta, Adrián Barreira-Manrique, Miguel Tamayo-Caro, Laura Vila-Vecilla, Leire Moreno-Cugnon, Nagore Beitia, Daniela Medrano, David Fernández-Ramos, Juan José Lozano, Satoshi Okawa, José L. Lavín, Natalia Martín-Martín, James D. Sutherland, Virginia Guitiérez de Juan, Monika Gonzalez-Lopez, Nuria Macías-Cámara, David Mosén-Ansorena, Liyam Laraba, C. Oliver Hanemann, Emanuela Ercolano, David B. Parkinson, Christopher W. Schultz, Marcos J. Araúzo-Bravo, Alex M. Ascensión, Daniela Gerovska, Haizea Iribar, Ander Izeta, Peter Pytel, Philipp Krastel, Alessandro Provenzani, Pierfausto Seneci, Ruben D. Carrasco, Antonio Del Sol, María Luz Martinez-Chantar, Rosa Barrio, Eduard Serra, Conxi Lazaro, Adrienne M. Flanagan, Myriam Gorospe, Nancy Ratner, Ana M. Aransay, Arkaitz Carracedo, Marta Varela-Rey, Ashwin Woodhoo

**Affiliations:** 1 Center for Cooperative Research in Biosciences (CIC bioGUNE), Basque Research and Technology Alliance (BRTA), Bizkaia Technology Park, Derio, Spain.; 2 Centro de Investigación Biomédica en Red de Enfermedades Hepáticas y Digestivas (CIBERehd), Instituto de Salud Carlos III, Madrid, Spain.; 3 Bioinformatic Platform, CIBERehd, Instituto de Salud Carlos III, Barcelona, Spain.; 4 Computational Biology Group, Luxembourg Centre for Systems Biomedicine (LCSB), University of Luxembourg, Belvaux, Luxembourg.; 5 Integrated BioBank of Luxembourg, Dudelange, Luxembourg.; 6 Centro de Investigación Biomédica en Red de Cáncer (CIBERONC), Instituto de Salud Carlos III, Madrid, Spain.; 7 Institute of Translational and Stratified Medicine, Faculty of Medicine and Dentistry, Plymouth University, Derriford Research Facility, Devon, United Kingdom.; 8 Department of Surgery, Thomas Jefferson University, Philadelphia, Pennsylvania, USA.; 9 Computational Biology and Systems Biomedicine, Biodonostia Health Research Institute, San Sebastián, Spain.; 10 IKERBASQUE, Basque Foundation for Science, Bilbao, Spain.; 11 Tissue Engineering Laboratory, Bioengineering Area, Instituto Biodonostia, San Sebastián, Spain.; 12 Department of Pathology, University of Chicago, Chicago, Illinois, USA.; 13 Novartis Institutes for Biomedical Research, Basel, Switzerland.; 14 Department of Cellular, Computational and Integrative Biology (CIBIO), University of Trento, Trento, Italy.; 15 Chemistry Department, University of Milan, Milan, Italy.; 16 Department of Oncologic Pathology, Dana-Farber Cancer Institute, Harvard Medical School, Boston, Massachusetts, USA.; 17 Moscow Institute of Physics and Technology, Dolgoprudny, Russia.; 18 Hereditary Cancer Group, Institute for Health Science Research Germans Trias I Pujol (IGTP) and Program of Predictive and Personalized Medicine of Cancer (PMPPC), Barcelona, Spain.; 19 Hereditary Cancer Program, Catalan Institute of Oncology, and; 20 Program in Molecular Mechanisms and Experimental Therapy in Oncology (Oncobell), Bellvitge Biomedical Research Institute (IDIBELL), Hospitalet de Llobregat, Barcelona, Spain.; 21 Department of Histopathology, Royal National Orthopaedic Hospital NHS Trust, Stanmore, United Kingdom.; 22 UCL Cancer Institute, University College London, London, United Kingdom.; 23 Laboratory of Genetics and Genomics, National Institute on Aging–Intramural Research Program, NIH, Baltimore, Maryland, USA.; 24 Division of Experimental Hematology and Cancer Biology, Department of Pediatrics, Cincinnati Children’s Hospital Medical Center, University of Cincinnati, Cincinnati, Ohio, USA.; 25 Biochemistry and Molecular Biology Department, University of the Basque Country (UPV/EHU), Bilbao, Spain.

**Keywords:** Cell Biology, Oncology, Cancer, Epigenetics, Oncogenes

## Abstract

Cancer cells can develop a strong addiction to discrete molecular regulators, which control the aberrant gene expression programs that drive and maintain the cancer phenotype. Here, we report the identification of the RNA-binding protein HuR/ELAVL1 as a central oncogenic driver for malignant peripheral nerve sheath tumors (MPNSTs), which are highly aggressive sarcomas that originate from cells of the Schwann cell lineage. HuR was found to be highly elevated and bound to a multitude of cancer-associated transcripts in human MPNST samples. Accordingly, genetic and pharmacological inhibition of HuR had potent cytostatic and cytotoxic effects on tumor growth, and strongly suppressed metastatic capacity in vivo. Importantly, we linked the profound tumorigenic function of HuR to its ability to simultaneously regulate multiple essential oncogenic pathways in MPNST cells, including the Wnt/β-catenin, YAP/TAZ, RB/E2F, and BET pathways, which converge on key transcriptional networks. Given the exceptional dependency of MPNST cells on HuR for survival, proliferation, and dissemination, we propose that HuR represents a promising therapeutic target for MPNST treatment.

## Graphical abstract

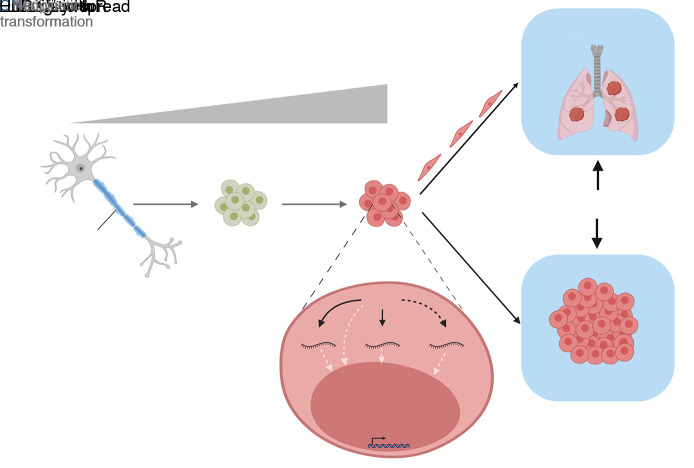

## Introduction

Malignant peripheral nerve sheath tumors (MPNSTs) are highly aggressive sarcomas with a strong metastatic potential, usually to the lung (1). MPNST patients are largely refractory to current treatments that include radical surgical resection and/or radiation and chemotherapy, and have very poor 5-year survival rates ranging between 15% and 50% (1). Half of MPNST cases arise in the context of neurofibromatosis type 1 syndrome (NF1), and these tumors are a leading cause of death in NF1 patients. In NF1, loss of the *NF1* tumor suppressor gene that encodes the Ras GTPase-activating protein neurofibromin leads to the development of benign neurofibromas that are located on the skin (cutaneous neurofibromas) or can be deep-seated in large peripheral nerves (plexiform neurofibromas). Plexiform neurofibromas can transform into MPNSTs, which can also occur spontaneously (sporadic MPNSTs) or after radiotherapy. There is general acceptance that cells of the Schwann cell lineage are the crucial neoplastic cells in MPNSTs (1, 2).

A number of mutations that drive MPNST pathogenesis have been identified, with a surprising degree of overlap in NF1-associated and sporadic forms. These include molecular variants of the *NF1* tumor suppressor gene that are present in all NF1 patients, and in a majority of sporadic and radiation-induced MPNSTs (2, 3). Other ancillary, yet essential, cancer-driving genetic aberrations include loss of the genes *CDKN2A*, *TP53*, *RB*, or *PTEN*, or the genes encoding the PRC2 components *SUZ12* or *EED*, and amplification of *PDGFRA*, *EGFR*, or *MET* (4, 5). In addition, recent studies have shown that activation of multiple signaling pathways, including the PI3K/AKT/mTOR, RAS/RAF-MEK-ERK, Wnt/β-catenin, and HIPPO-YAP/TAZ pathways, and other less ubiquitous molecular alterations involving aurora kinases and transcription factors (TFs) such as SOX9, also contribute to MPNST pathogenesis (1, 3, 6).

Gene dysregulation is a hallmark of cancer cells. Genetic alterations in cancer cells invariably lead to a global remodeling of their transcriptome, allowing them to acquire advanced functional capabilities for survival, proliferation, and dissemination. MPNSTs have a unique transcriptomic signature that is clearly distinct from normal or even neurofibroma-derived primary Schwann cells or tumors (7, 8), and strongly associated with key Schwann cell developmental programs, including control of survival and proliferation. Targeting dysregulated gene expression programs in cancers has emerged as a promising therapeutic strategy, and there is an intense focus on identifying the key molecular regulators that govern these programs (9, 10). In particular, RNA-binding proteins (RBPs) are increasingly recognized as attractive targets because of their ability to regulate the type and abundance of hundreds of transcripts by modulating every aspect of their post-transcriptional life — splicing, transport, localization, translation, stabilization, and decay. Furthermore, each RBP can bind to multiple overlapping groups of functionally related RNAs, forming “RNA regulons” that control many biological functions (11).

We have previously shown that the ubiquitously expressed RBP HuR/ELAVL1 was highly expressed in immature Schwann cells, a stage of development characterized by a peak in Schwann cell proliferation and apoptosis. We found that HuR was bound to and regulated several key mRNAs, coordinately regulating them at the post-transcriptional level (12). Subsequently, as immature Schwann cells differentiated, we found that they lost expression of HuR, and the production of HuR targets encoding proliferation and apoptosis proteins was downregulated. Notably, many of the HuR targets in immature Schwann cells become re-expressed in MPNSTs, and the encoded proteins play key roles in tumor growth, as shown for SOX9 (8) and BRD4 (13). HuR is frequently upregulated in different cancer types (14), leading us to hypothesize that HuR could become re-expressed in MPNSTs, where it would have a key role in driving the dysregulated transcriptomic programs. Here, we present evidence that HuR is potently tumorigenic in MPNSTs and that suppressing HuR expression reduces tumor growth and metastasis. We propose that the malignant influence of HuR is linked to enhancing multiple key oncogenic programs operating in MPNST cells.

## Results

### HuR is upregulated in human MPNSTs.

**Figure 1:**
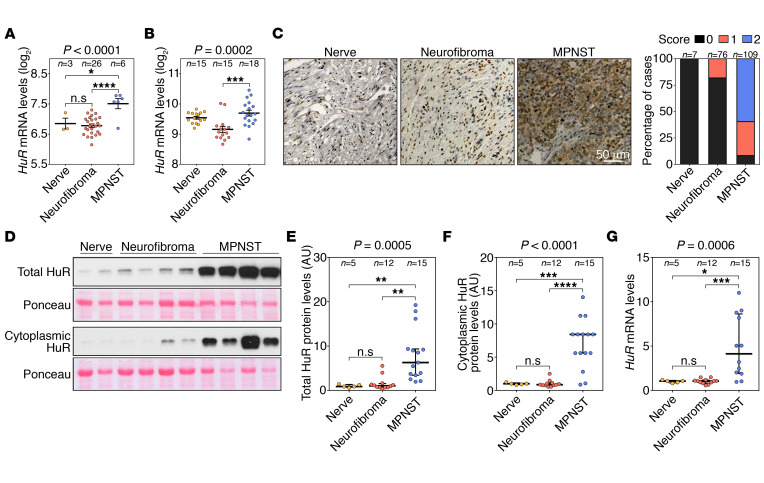
HuR is upregulated in human MPNSTs. (**A** and **B**) *HuR* mRNA levels in nerves, neurofibromas, and MPNSTs from patients (**A**) and mouse models (**B**) from the Jessen cohort (GSE41747) (7). (**C**) Representative immunohistochemistry micrographs of endogenous HuR protein levels (brown) in a tissue microarray panel of human nerves (*n* = 7), benign neurofibromas (*n* = 76), and MPNSTs (*n* = 109) (15). Score 0, low HuR staining; 1, intermediate staining; 2, high HuR staining. Scale bar: 50 μm. (**D**–**G**) Western blot and quantitative reverse transcriptase PCR (RT-qPCR) analysis of HuR levels in a panel of human nerves (*n* = 5), benign neurofibromas (*n* = 12), and MPNSTs (*n* = 15) obtained from Stanmore Musculoskeletal Biobank. (**D**) Representative immunoblots showing total and cytoplasmic HuR levels in a selection of samples. (**E** and **F**) Graphs representing densitometry analysis of total HuR protein levels, corrected for Ponceau red signals (**E**), and cytoplasmic HuR protein levels, corrected for Ponceau red signals (**F**). (**G**) *HuR* mRNA levels as measured by RT-qPCR analysis. Data are presented as mean ± SEM (**A** and **B**) or median with interquartile range (**E**–**G**); 1-way ANOVA with Tukey’s multiple-comparisons test. *P < 0.05; **P < 0.01; ***P < 0.001; ****P < 0.0001. The number of samples (*n*) per group is indicated.

To explore the potential role of HuR in Schwann cell cancers, we searched a publicly available expression data set (Gene Expression Omnibus [GEO] GSE41747) (7) and found that *HuR/ELAVL1* mRNA levels were significantly upregulated in MPNSTs, both in patients and in mouse samples (Figure 1, A and B). Next, we analyzed HuR protein abundance by immunohistochemistry in a human tissue microarray panel comprising normal nerves (*n* = 7), benign neurofibromas (*n* = 76), and MPNSTs (*n* = 109) (15) and, similarly, found a strong upregulation of HuR protein expression in MPNSTs (Figure 1C). Finally, we validated these results by examining HuR protein and mRNA expression in an independent cohort of frozen human normal nerves (*n* = 5), neurofibromas (*n* = 12), and MPNSTs (*n* = 15), obtained from the Stanmore Musculoskeletal Biobank (United Kingdom). We confirmed that total HuR protein levels were significantly elevated in the MPNST samples (Figure 1, D and E). We also examined cytoplasmic HuR levels, since HuR export from the nucleus to the cytoplasm is linked to its function as a post-transcriptional regulator of target mRNAs (16). As shown (Figure 1, D and F), cytoplasmic HuR levels were higher in MPNST samples. *HuR* mRNA levels were also higher in these samples (Figure 1G).

We did not find significant differences in the abundance of *HuR* mRNA or protein when comparing dermal neurofibromas and plexiform neurofibromas from our frozen cancer panel (Supplemental Figure 1A; supplemental material available online with this article; https://doi.org/10.1172/JCI130379DS1; see complete unedited blots in the supplemental material), nor were there differences in *HuR* mRNA levels in publicly available data sets (Supplemental Figure 1, B and C). Notably, we also did not find significant differences in the levels of HuR mRNA or protein when comparing NF1-derived and sporadic MPNSTs (Supplemental Figure 1, D and E).

The high abundance and cytoplasmic localization of HuR point to a potentially important role in both sporadic and NF1-derived MPNSTs.

### RIP-Chip identifies HuR mRNA targets associated with key cancer traits.

To examine the biological significance of the high HuR levels in MPNSTs, we analyzed HuR-associated mRNAs on a genome-wide scale by ribonucleoprotein immunoprecipitation (RIP) under conditions that preserve endogenous RNA-protein interactions, followed by microarray detection of bound mRNAs (RIP-Chip) (17, 18). Notably, RIP preferentially enriches for the stably bound subsets of putative mRNA targets rather than transient targets that might be detected by ribonucleoprotein cross-linking. This favors the identification of targets forming part of functional complexes that lead to mRNA target stabilization and increased translation (19).

**Figure 2:**
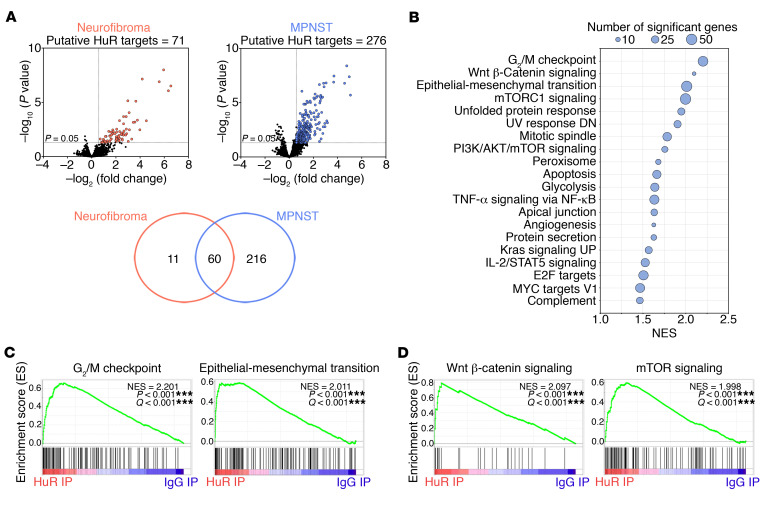
HuR is bound to key targets in human MPNSTs. (**A**) Volcano plots show enrichment of transcripts most significantly bound to HuR compared with control IgG in neurofibromas (left) (*n* = 8) and MPNSTs (right) (*n* = 12) obtained from Stanmore Musculoskeletal Biobank. Red or blue dots, fold change >1.5; adjusted *P* value < 0.05. Venn diagram shows overlap between putative HuR mRNA targets from neurofibromas and MPNSTs. (**B**) GSEA analysis of putative HuR mRNA targets in MPNSTs for MSigDB hallmarks. The top 20 gene sets (FDR iQ values < 0.1) are plotted relative to normalized enrichment score (NES). Circles denote the number of enriched genes in each category. (**C** and **D**) GSEA plots of HuR IP and control IgG IP for key cancer traits (**C**) and oncogenic pathways (**D**) in MPNSTs.

RIP analysis was performed using an anti-HuR antibody or control anti-IgG antibody and cytoplasmic extracts from human neurofibroma (*n* = 8) and MPNST (*n* = 12) samples from the aforementioned frozen cancer panel, and the isolated RNA was identified by microarray analysis (Supplemental Figure 2, A and B). Background mRNAs identified in side-by-side control immunoprecipitation reactions using mouse anti-IgG antibody were subtracted to identify bona fide HuR-interacting mRNAs. We found that HuR was specifically bound to 71 and 276 mRNAs in neurofibroma and MPNST samples, respectively (Figure 2A and Supplemental Tables 1 and 2). The large majority of neurofibroma targets (60 of 71; 85%) were also associated with HuR in the MPNST samples, whereas 216 HuR-bound transcripts were exclusively expressed in MPNST samples (Figure 2A), in line with the high HuR expression in MPNST samples, and supporting a role for HuR in malignant Schwann cell tumors.

To identify molecular pathways associated with HuR-bound transcripts in MPNSTs, we performed gene set enrichment analysis (GSEA) using Molecular Signatures Database (MSigDB) hallmarks (20). GSEA revealed significant enrichment of signatures associated with oncogenic traits including cell cycle progression, epithelial-mesenchymal transition, and angiogenesis (21), as well as signatures for the key oncogenic TFs MYC and E2F (refs. 22, 23, and Figure 2B). In addition, key signaling pathways in MPNSTs, including the Wnt/β-catenin, PI3K/AKT/mTOR, and RAS pathways (1, 2), were also enriched (Figure 2, B–D, and Supplemental Table 3).

Together, these data point to a potentially important biological function of HuR in MPNSTs, possibly regulating key signaling pathways that control oncogenic traits in these malignant tumors.

### HuR promotes MPNST cell growth in vitro and in vivo.

To characterize the functional importance of HuR, we first evaluated its abundance in established MPNST cell lines. Using a publicly available expression data set (GEO GSE14038) (8), we found that *HuR* mRNA levels were significantly upregulated in MPNST cell lines compared with neurofibroma-derived Schwann cells (Supplemental Figure 3A). Furthermore, higher *HuR* mRNA and protein levels were observed in 4 commonly used MPNST cell lines — ST88-14, 90-8, S462, and STS-26T — compared with freshly isolated Schwann cells derived from human nerves (Supplemental Figure 3, B and C).

We then silenced HuR in these 4 MPNST cell lines by lentiviral delivery of 2 distinct HuR-specific shRNAs and examined cellular growth. Three of the cell lines (ST88-14, 90-8, and S462) were derived from NF1 patients with confirmed loss of heterozygosity at the *NF1* locus for all 3 cell lines; the 90-8 cell line additionally showed a known microdeletion of *NF1*, whereas the sporadic MPNST line STS-26T had no mutations detected in the 60 exons of the *NF1* gene. These MPNST cell lines share a common gene expression profile distinct from that of normal Schwann cells, although they differ in proliferation rates and in expression of cell cycle proteins (24). Both constitutively expressed shRNAs reduced HuR levels efficiently, and suppressed cell growth, as shown by measurement of ATP levels and by direct cell counts in all 4 cell lines (Supplemental Figure 3, D–F). Furthermore, HuR silencing significantly reduced the clonogenic and anchorage-independent growth capacity of all 4 cell lines (Supplemental Figure 4, A–C).

**Figure 3:**
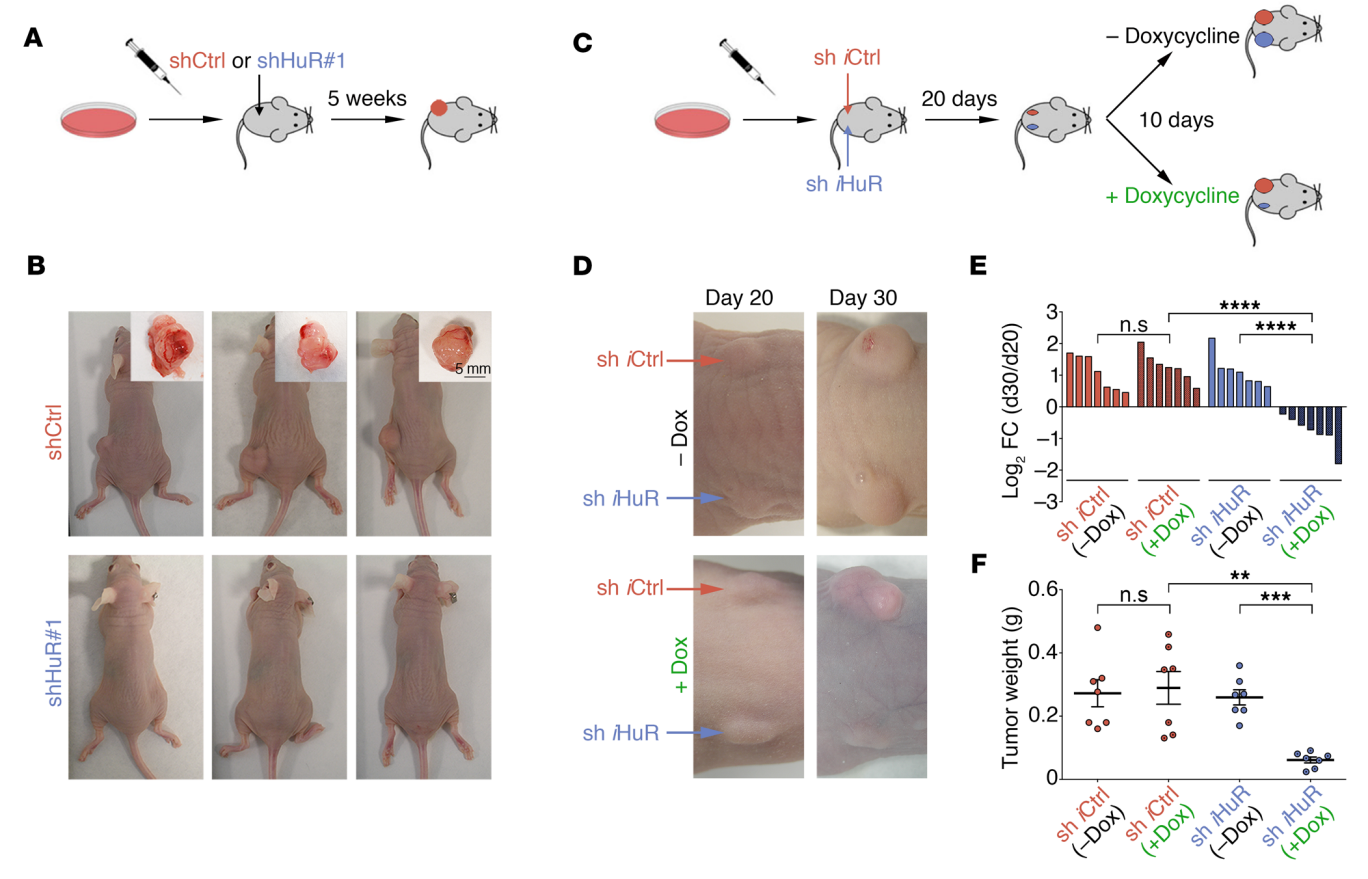
HuR promotes MPNST cell growth in vivo. (**A** and **B**) Constitutive HuR silencing prevents tumor formation in vivo. (**A**) Schematic representation of xenotransplantation experiments. (**B**) Representative pictures of tumors from nude mice injected with shCtrl or shHuR#1 STS-26T MPNST cells 5 weeks after transplant (*n* = 5) for each condition. Scale bar: 5 mm. (**C**–**F**) Inducible HuR silencing promotes tumor regression in vivo. (**C**) Schematic representation of xenotransplantation experiments using inducible HuR-silencing strategy. (**D**) Representative pictures of tumors from nude mice injected with sh *i*Ctrl or sh *i*HuR STS-26T MPNST cells on left and right flank, respectively, at 20 days after injection (day 20) and 10 days later (day 30), with (+ Dox) or without doxycycline diet (– Dox). (**E**) Waterfall plot showing change in tumor volume (represented as log_2_ fold change) of individual tumors formed at 20 days after transplant and after 10 days with or without doxycycline treatment for each of 4 groups of mice. sh *i*Ctrl (–Dox) *n* = 7; sh *i*Ctrl (+Dox) *n* = 7; sh *i*HuR (–Dox) *n* = 7; sh *i*HuR (+Dox) *n* = 7. (**F**) Graph showing weight of excised tumors for the 4 groups of mice. Each data point represents 1 mouse; 1-way ANOVA with Tukey’s multiple-comparisons test. **P < 0.01; ***P < 0.001; ****P < 0.0001.

Since the in vitro consequences of HuR perturbation were shared by all MPNST cell lines, we selected as representative STS-26T for in vivo validation. STS-26T cells in which HuR levels were normal or reduced by silencing were implanted subcutaneously in immunodeficient mice. Subsequent analysis revealed that whereas control cells efficiently formed tumors (5/5), tumor formation was completely abolished in HuR-silenced cells (0/5) (Figure 3, A and B).

Next, we examined the effects of HuR depletion on the growth of already established tumors. To this end, we used a doxycycline-inducible lentiviral shRNA system targeting HuR. STS-26T cells expressing pTRIPZ-shControl (sh *i*Ctrl) or pTRIPZ-shHuR (sh *i*HuR) were injected subcutaneously in the left and right flanks of immunodeficient mice, respectively, and tumors were allowed to form for about 20 days until they were palpable (~100 mm^3^ average). Mice were randomly assigned to standard chow diet or doxycycline diet for a period of 10 days, whereupon mice were sacrificed and tumors extracted (Figure 3C). Remarkably, induction of *HuR* shRNA by doxycycline treatment severely blunted tumor growth, and even led to visible tumor regression (Figure 3D). Tumor regression was observed in all mice following HuR depletion, and on average tumors shrank by 40% (Figure 3E) and weighed significantly less than control tumors (Figure 3F). HuR-depleted cells formed smaller tumors, and the efficiency of HuR silencing was confirmed in those tumors by Western blotting analysis (Supplemental Figure 5, A and B). Notably, tumors arising from sh *i*HuR cells without doxycycline treatment were indistinguishable in all parameters from tumors arising from sh *i*Ctrl cells with or without doxycycline treatment, supporting the efficiency and specificity of the inducible system used.

We also documented a marked reduction in the proliferation marker Ki67 and a rise in an apoptotic marker, active caspase-3, in HuR-depleted tumors (Supplemental Figure 5C). We validated these results in the panel of cultured MPNST cell lines, where, similarly, genetic depletion of HuR in the 4 cell lines potently reduced cell proliferation as measured by (a) cell cycle analysis by flow cytometry, which showed a general increase in the percentage of cells in the G_1_ phase and a decrease in cells in the S and G_2_/M phases, consistent with a G_1_ cell cycle arrest, and (b) BrdU incorporation (Supplemental Figure 6, A and B). We also found a marked increase in senescence after HuR silencing in all 4 cell lines (Supplemental Figure 6C). Detection of annexin V by flow cytometry indicated a trend toward increased apoptosis in all 4 cell lines after HuR knockdown when cells were cultured under basal, growth-promoting conditions (10% serum). Exposure to cellular stress, such as culture of cells under growth-limiting conditions (2% serum) (13), exaggerated this effect and led to a marked increase in apoptotic and necrotic cell death after HuR depletion (Supplemental Figure 6D).

Together, these data indicate that HuR plays an important functional role in MPNST cell biology by controlling key features such as survival, proliferation, and replicative immortality.

### HuR promotes MPNST metastasis in vivo.

MPNSTs have a high metastatic potential, and up to 50% of patients develop metastatic disease, usually to the lung, further worsening 5-year survival rates of patients (1, 25). Yet, metastasis is one of the least understood aspects of MPNSTs. Given the profound antitumor effects of HuR in MPNST cells, and the identification of epithelial-mesenchymal transition signature as one of the top enriched categories of HuR targets from GSEA analysis (Figure 2, B and C), we decided to examine whether HuR could be important for MPNST metastasis, using a surrogate model of lung metastasis. Briefly, this model measures the ability of cells injected in the lateral tail vein of immunodeficient mice to survive in circulation, arrest at a distant organ, extravasate, adapt to growth in the foreign microenvironments of a distant tissue, and grow into metastatic lesions, recapitulating many of the essential late steps in metastasis (26).

**Figure 4:**
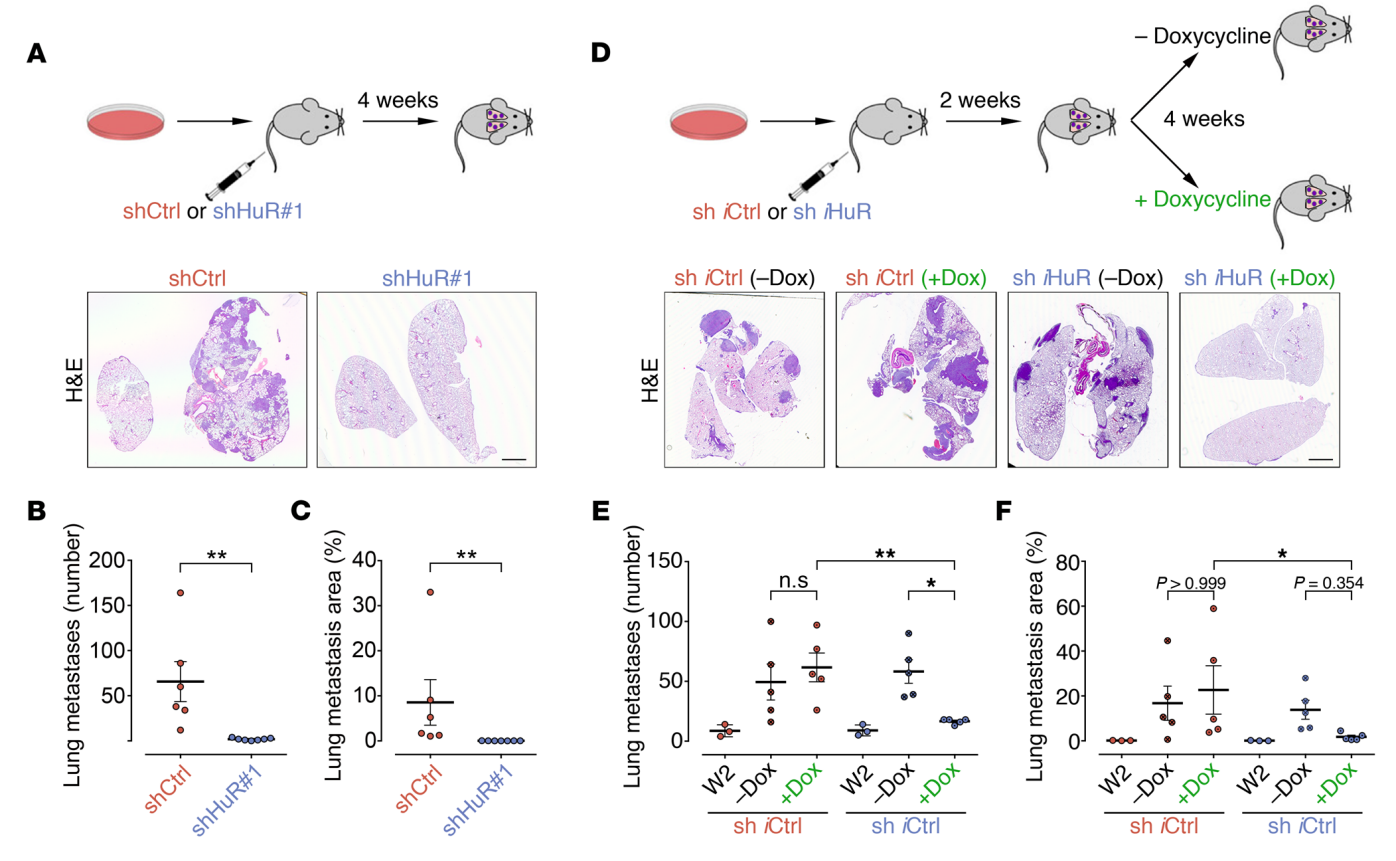
HuR promotes MPNST metastasis in vivo. (**A**–**C**) Constitutive HuR silencing prevents lung metastasis of STS-26T MPNST cells. (**A**) Schematic representation of lung metastasis experiments. shCtrl (*n* = 6) or shHuR#1-expressing (*n* = 7) STS-26T MPNST cells were injected in the tail vein of nude mice, and lung architecture analyzed by H&E staining 4 weeks later. Representative pictures of lung histology are shown. Scale bar: 2 mm. (**B** and **C**) Number of lung metastases (**B**) and lung metastatic area, expressed as a percentage of total lung area (**C**), were quantified by H&E histology. (**D**–**F**) Inducible HuR silencing prevents growth of established lung metastatic nodules. (**D**) Schematic representation of experiments. sh *i*Ctrl or sh *i*HuR–expressing STS-26T MPNST cells were injected in the tail vein of nude mice. A group of mice (*n* = 3 for each condition) was sacrificed at 2 weeks (W2) to analyze basal formation of lung metastases, and the rest fed with normal diet (–Dox) or doxycycline diet to induce expression of shRNAs (+Dox) for a further 4 weeks before analysis of lung histology by H&E staining. Representative pictures of lung histology for the following groups are shown: sh *i*Ctrl with normal diet (sh *i*Ctrl; –Dox) *n* = 5; sh *i*Ctrl with doxycycline diet (sh *i*Ctrl; +Dox) *n* = 5, sh *i*HuR with normal diet (sh *i*HuR; –Dox) *n* = 5, sh *i*HuR with doxycycline diet (sh *i*HuR; +Dox) *n* = 5. Scale bar: 2 mm. (**E** and **F**) Number of lung metastases (**E**) and lung metastatic area, expressed as a percentage of total lung area (**F**), were quantified. Each data point represents 1 mouse. Data are presented as mean ± SEM; 2-tailed unpaired Mann-Whitney *U* test (**B** and **C**); 1-way ANOVA with Holm-Šidák multiple-comparisons test (**E** and **F**). *P < 0.05; **P < 0.01.

We silenced HuR constitutively in STS-26T cells, an MPNST cell line that exhibits metastatic tropism to the lung (27), injected them in the tail vein, and examined metastatic colonization of the lung 4 weeks later by histology (Figure 4A). Remarkably, very few metastatic lesions were observed in HuR-depleted cells in contrast to control cells, which formed numerous and large metastatic foci (Figure 4, B and C). Next, we used our inducible lentiviral silencing system to examine the effect of HuR silencing on the colonization step of the metastatic process, i.e., the survival of the cells in these foreign microenvironments during the metastatic process, and the reactivation of their proliferation programs to form overt metastatic lesions. We injected STS-26T cells expressing pTRIPZ-shControl (sh *i*Ctrl) or pTRIPZ-shHuR (sh *i*HuR) in the tail vein, and allowed them to begin to form metastases for 2 weeks. Mice were randomly assigned to standard chow or doxycycline-containing diet for 4 weeks, whereupon mice were sacrificed and lungs extracted for histological analysis (Figure 4D). Induction of *HuR* shRNA by doxycycline treatment blocked the conversion of these micrometastases into the macroscopic neoplastic growth seen in the control cells with or without doxycycline treatment and in sh *i*HuR cells without doxycycline treatment (Figure 4, D–F).

Together, these data support a key role for HuR in MPNST metastasis, potentially controlling key aspects of the metastatic process, from the survival of the cells in the bloodstream to their colonization and proliferation within distant organs.

### HuR overexpression in Schwann cells is not sufficient to trigger oncogenic transformation or dissemination.

Since HuR is expressed at notably higher levels in MPNST compared with Schwann cells (Figure 1 and Supplemental Figure 3, A–C), we next examined whether elevating HuR levels in human Schwann cells was sufficient to elicit cell transformation and dissemination.

We ectopically expressed HA-tagged HuR using a doxycycline-inducible lentiviral system (28) in immortalized normal human Schwann cells (iHSCλ2) (Supplemental Figure 7A), and found that this led to a small but significant increase in cell growth, as shown by measurement of ATP levels and by direct cell counts, and increased the clonogenic and anchorage-independent growth capacity (Supplemental Figure 7B). However, no major effect was observed on tumor formation in vivo using mouse xenograft models (Supplemental Figure 7, C and D). We also did not observe any major effect on tumor formation after ectopic expression of HuR in immortalized plexiform neurofibroma–derived human Schwann cells (ipNF SC). These data suggest that while HuR overexpression promotes proliferation in Schwann cells in culture, it is not sufficient to induce Schwann cell–derived tumors in mice.

Similarly, we examined metastatic properties using tail vein injections of control or HuR-overexpressing normal and plexiform neurofibroma-derived human Schwann cell lines. We did not find any metastatic nodules in the controls of either cell line. Unexpectedly, we found the presence of small micrometastases in HuR-overexpressing normal Schwann cell lines (1 of 4) and plexiform human Schwann cell lines (3 of 4) (Supplemental Figure 7E), although these were far fewer and smaller than after injection of STS-26T MPNST cell line (Figure 4).

Taken together, our results showed that HuR overexpression in normal or plexiform neurofibroma Schwann cells was not sufficient to trigger oncogenic transformation but modestly increased their metastatic capacity.

### Pharmacological inhibition of HuR reduces MPNST growth and metastasis.

Our results demonstrate that elevated HuR levels in MPNST cells are required for cell growth and metastasis. We next ascertained whether the inhibition of HuR could be exploited as a therapeutic strategy using small-molecule HuR inhibitors.

**Figure 5:**
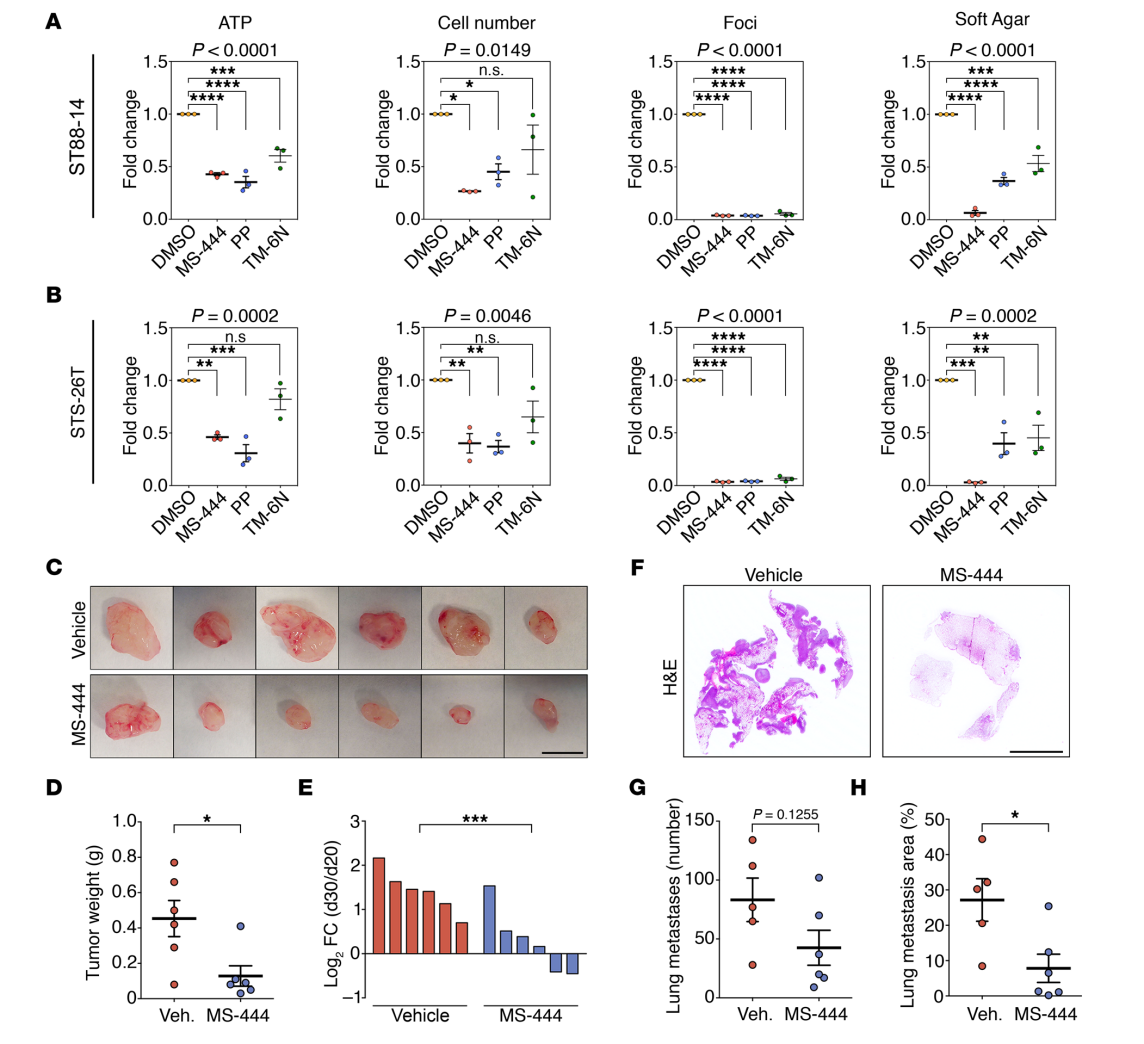
Pharmacological inhibition of HuR blocks MPNST cell growth and metastasis in vitro and in vivo. (**A** and **B**) Pharmacological inhibition of HuR activity leads to a reduction in cell growth in MPNST cell lines ST88-14 and STS-26T, as determined by ATP luminescence, counts of cell numbers, clonogenic assays (foci), and anchorage-independent growth using soft agar assays. Graphs represent absorbance of crystal violet–stained colonies for clonogenic assays and number of colonies in soft agar assays. Data are normalized to DMSO-treated and are presented as mean ± SEM. Each data point represents 1 independent experiment; 1-way ANOVA with Tukey’s multiple-comparisons test. (**C**–**E**) Pharmacological inhibition of HuR activity by MS-444 promotes tumor regression in vivo. (**C**) Pictures of tumors from nude mice injected with STS-26T MPNST cells after vehicle or MS-444 treatment. Scale bar: 10 mm. (**D**) Graph showing weight of excised tumors for both groups of mice. (**E**) Waterfall plot showing change in tumor volume (represented as log_2_ fold change) of individual tumors formed at 20 days after transplant, and after 10 days with pharmacological inhibition. Each data point represents 1 mouse. Data are presented as mean ± SEM; 2-tailed unpaired Mann-Whitney *U* test. (**F**–**H**) Pharmacological inhibition of HuR activity by MS-444 prevents growth of established lung metastatic nodules in vivo. (**F**) Representative pictures of lung histology from nude mice injected with STS-26T MPNST cells after vehicle or MS-444 treatment. Scale bar: 5 mm. (**G** and **H**) Number of lung metastases (**G**) and lung metastatic area, expressed as a percentage of total lung area (**H**), were quantified. Each data point represents 1 mouse. Data are presented as mean ± SEM; 2-tailed unpaired Mann-Whitney *U* test. *P < 0.05; **P < 0.01; ***P < 0.001; ****P < 0.0001.

Pharmacological inhibition of HuR with MS-444, which inhibits HuR homodimerization to prevent the binding of 3′-UTR AU-rich elements (29); pyrvinium pamoate, an FDA-approved anthelmintic drug that blocks HuR nucleocytoplasmic translocation (30); and tanshinone mimic 6N (TM-6N), which inhibits HuR-RNA complex formation (31), each strongly reduced MPNST cell growth in culture in ST88-14 and STS-26T cells (Figure 5, A and B), similarly to what we found through the genetic inhibition of HuR (Supplemental Figures 3 and 4).

Importantly, i.p. injection with MS-444 (25 mg/kg body weight; treatment every 48 hours for 10 days) of nude mice bearing already established tumors (formed 20 days after xenograft transplant of STS-26T cells in flanks) severely blunted tumor growth, and even led to tumor regression in some cases. In addition, MS-444–treated tumors weighed significantly less than control tumors (Figure 5, C–E). Remarkably, MS-444 treatment of nude mice (i.p. injection every 48 hours for 2 weeks; 25 mg/kg body weight) with established micrometastases that had formed for 2 weeks after i.v. injection of STS-26T cells also reduced the conversion of these micrometastases into the macroscopic neoplastic growth, and smaller metastatic areas were measured in MS-444–treated mice than in vehicle-treated mice (Figure 5, F–H).

Our results show that pharmacological inhibition of HuR reduces MPNST growth and metastasis, thus highlighting the relevance of HuR as a potential therapeutic target for MPNSTs.

### HuR regulates key oncogenic transcriptional programs.

**Figure 6:**
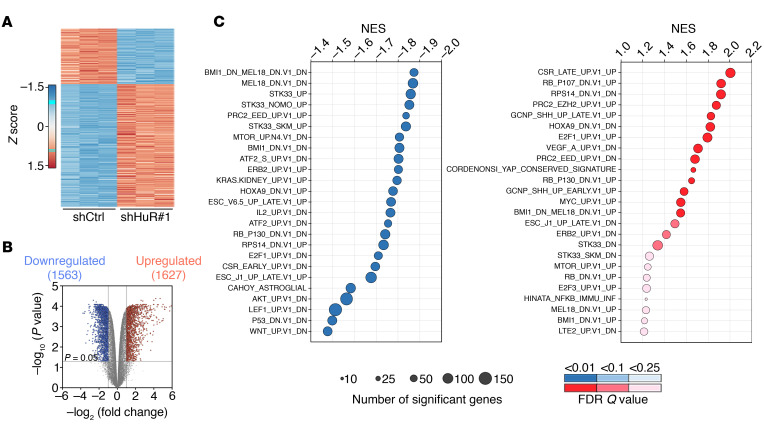
RNA-Seq reveals that HuR controls key oncogenic pathways in MPNSTs. (**A**) Heatmap representation of differentially expressed genes between shCtrl (*n* = 3) or shHuR#1-expressing (*n* = 3) ST88-14 MPNST cells (fold change >2; adjusted *P* value < 0.05). (**B**) Volcano plot of transcriptome profiles between shCtrl (*n* = 3) and shHuR#1-expressing (*n* = 3) ST88-14 MPNST cells. Red and blue dots represent genes significantly upregulated and downregulated, respectively, in shHuR#1-expressing cells (fold change >2; adjusted *P* value < 0.05). (**C**) GSEA analysis of shCtrl and shHuR#1-expressing ST88-14 MPNST cells for MSigDB oncogenic signatures. Gene sets with FDR *Q* values less than 0.25 are plotted relative to normalized enrichment score (NES). Categories with negative (left) and positive (right) NES are down- or upregulated, respectively, in shCtrl cells. Circles denote the number of enriched genes in each category, and colors represent FDR *Q* values as indicated.

Next, we sought to define the mechanisms by which HuR exerts such profound effects on MPNST cells. We silenced HuR expression using lentiviral vectors expressing constitutive shRNA directed at HuR in the ST88-14 cell line, and performed RNA-Seq profiling. We identified distinct transcriptomic profiles between the control and HuR-depleted cells (Figure 6A), and differential expression analysis revealed that a notable proportion of the transcriptome in MPNST cells changed significantly. There were similar numbers of significantly upregulated and downregulated transcripts exhibiting more than 2-fold changes (1563 and 1627, respectively) (Figure 6B and Supplemental Table 4).

GSEA analysis revealed an inhibition of several tumorigenic pathways in MPNST cells by HuR silencing, including the YAP/TAZ, Wnt/β-catenin, PI3K/AKT/mTOR, and RAS pathways, in addition to signatures for the key oncogenic TFs MYC and E2F (Figure 6C and Supplemental Tables 5 and 6). There was also significant overlap between the signature sets we identified by this RNA-Seq analysis and the RIP-Chip analysis for putative HuR mRNA targets (Figure 2B). These results strongly suggested that HuR could be affecting MPNST cells by directly regulating these pathways, several of which have already been established to play key roles in MPNST tumorigenesis (6, 7, 13, 32–34). We thus set out to examine the importance of HuR for these pathways.

**Figure 7:**
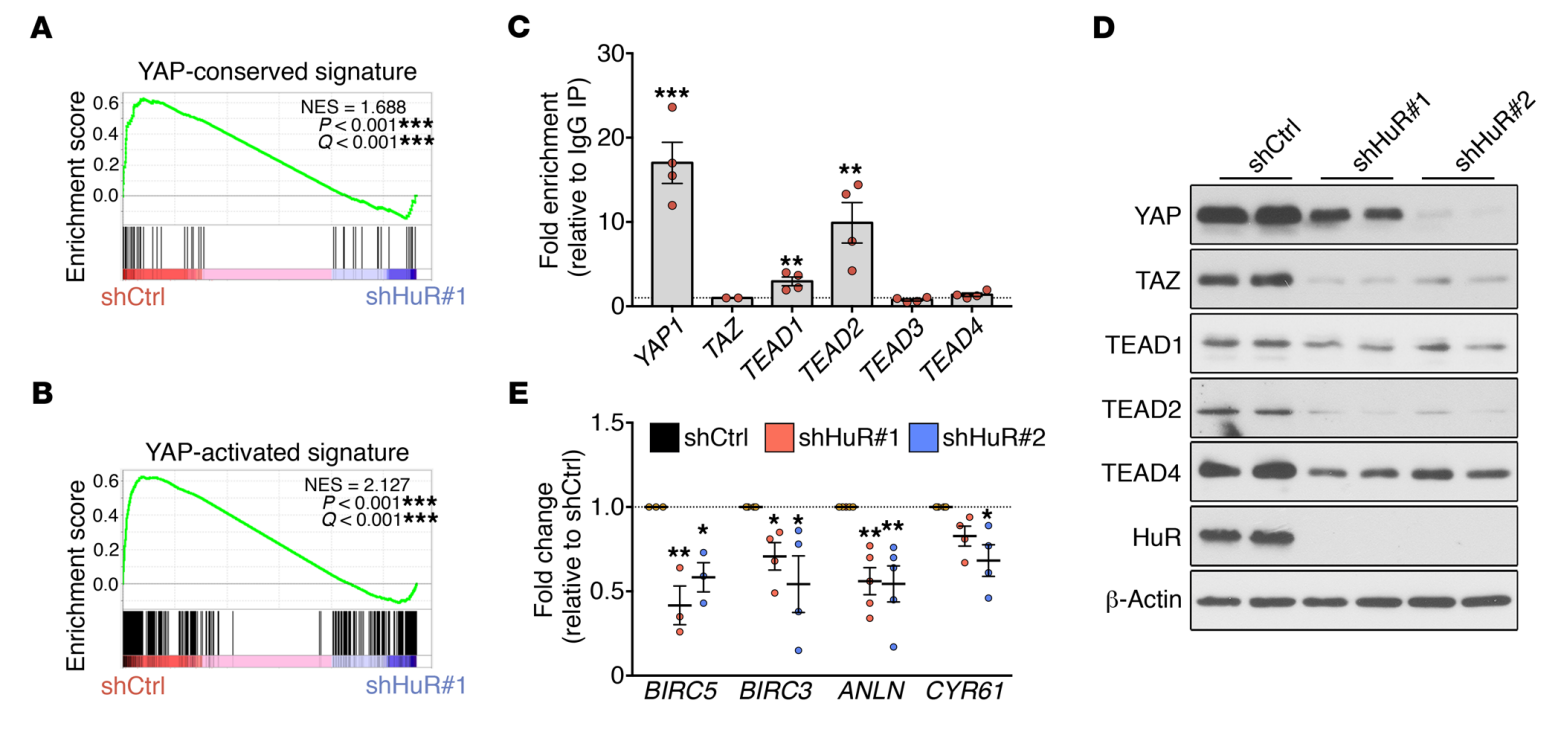
HuR regulates the YAP/TAZ pathway in MPNSTs. (**A** and **B**) GSEA plots showing enrichment of YAP-conserved signature from Figure 6C (**A**) and YAP-activated signature set (36) (**B**) in shCtrl-infected compared with shHuR#1-infected ST88-14 MPNST cells. (**C**) RIP-qPCR analysis showing binding of HuR to *YAP1*, *TEAD1*, and *TEAD2* in 4 MPNST cell lines (ST88-14, 90-8, S462, and STS-26T). Data are normalized to control IgG IPs and are presented as mean ± SEM, 2-tailed unpaired Student’s *t* test; *n* = 4 MPNST cell lines. (**D**) Representative Western blots showing a general downregulation of key YAP/TAZ pathway components after HuR silencing in ST88-14 MPNST cells. Technical duplicates are shown, and similar results were obtained in at least 3 independent experiments. (**E**) RT-qPCR analysis showing downregulation of YAP/TAZ pathway effector genes (6) after HuR silencing in ST88-14 MPNST cells. Data are normalized to shCtrl cells and are presented as mean ± SEM. Each data point represents 1 independent experiment; 1-way ANOVA with Tukey’s multiple-comparisons test. *P < 0.05; **P < 0.01; ***P < 0.001.

We first focused on the YAP/TAZ pathway. In an elegant study, it was recently shown that human MPNSTs exhibit elevated HIPPO-TAZ/YAP expression, and hyperactivation of YAP/TAZ in Schwann cells activated an oncogenic program with development of MPNSTs (6). GSEA analysis of RNA-Seq data showed that HuR silencing in MPNST cells led to a strong suppression of a YAP-conserved signature (35) as well as a functionally validated YAP-activated signature (ref. 36 and Figure 7, A and B). Notably, GSEA also showed a strong negative correlation of genes regulated by YAP/TAZ hyperactivation in Schwann cells (6) and genes regulated by HuR silencing in ST88-14 MPNST cells (Supplemental Figure 8A), suggesting that HuR could be responsible for regulating expression of YAP/TAZ pathway components.

To test this hypothesis, we examined whether HuR associated with mRNAs encoding key protein components of the YAP/TAZ pathway by performing RIP followed by reverse transcription and quantitative PCR analysis (RIP-qPCR) in MPNSTs. To avoid possible confounding effects of cell type heterogeneity in tumor samples, we used MPNST cell lines instead. We found that there was a strong enrichment of *YAP1* mRNA, encoding YAP1, and *TEAD1* and *TEAD2* mRNAs, encoding TEAD1 and TEAD2, two transcriptional partners of YAP1, in HuR RIP samples (Figure 7C). Notably, HuR depletion led to a striking reduction in the levels of these proteins in both the NF1-derived ST88-14 cell line (Figure 7D) and the sporadic cell line STS-26T (Supplemental Figure 8B). Suppressing the YAP/TAZ pathway by HuR silencing reduced the mRNA levels of downstream YAP/TAZ target genes in both cell lines (Figure 7E and Supplemental Figure 8C). Interestingly, although we did not find that *TAZ* or *TEAD4* mRNAs were direct targets of HuR, HuR depletion led to a strong reduction in their expression levels, likely due to an indirect effect on their transcription, mRNA stability, or translation.

Our results indicate that HuR can regulate expression of key YAP/TAZ pathway components in MPNST cells, which could lead to the aberrant elevated HIPPO-TAZ/YAP expression seen in mouse and human tumor tissue samples and the hyperactivation of this essential oncogenic pathway for MPNST pathogenesis.

### HuR regulates key cell cycle genes in MPNSTs via an RB/E2F axis.

**Figure 8:**
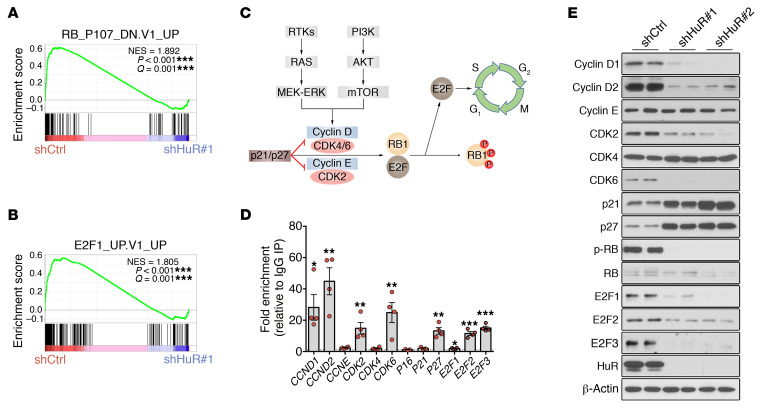
HuR regulates the RB/E2F pathway in MPNSTs. (**A** and **B**) GSEA plots showing enrichment of functionally defined RB (**A**) and E2F1 (**B**) signature set in shCtrl-infected compared with shHuR#1-infected ST88-14 MPNST cells from Figure 6C. (**C**) Cartoon depicting regulatory components of the RB/E2F pathway in cell cycle regulation. By interacting with CDKs, cyclins form complexes (cyclin D with CDK4/6 and cyclin E with CDK2) that phosphorylate RB1 (phosphorylated RB1 is inactive). When RB1 is phosphorylated, E2F is released and can transcriptionally activate its target genes, enabling the G_1_/S transition of cell cycle. Cyclins can be regulated at the transcription level by the RAS-MEK-ERK pathway and at the translation level by mTOR via S6K and 4EBP1. p21 and p27 can bind to and inhibit cyclin-CDK complexes. (**D**) RIP-qPCR analysis showing binding of HuR to multiple genes in the RB/E2F pathway in 4 MPNST cell lines (ST88-14, 90-8, S462, STS-26T). Data are normalized to control IgG IPs and are presented as mean ± SEM, 2-tailed unpaired Student’s *t* test. (**E**) Representative Western blots showing a downregulation of several key RB/E2F pathway components after HuR silencing in ST88-14 MPNST cells, in general concordance with RIP-qPCR data. Technical duplicates are shown, and similar results were obtained in 3 independent experiments. *P < 0.05; **P < 0.01; ***P < 0.001.

RAS/MEK/ERK and PI3K/AKT/mTOR are 2 other major signaling pathways that are upregulated in MPNSTs and have important roles in MPNST pathogenesis (1, 2). GSEA analysis revealed that many components of these pathways were significantly downregulated by HuR silencing, including activation of receptor tyrosine kinases such as PDGF (Supplemental Figure 9, A and B). Along the same lines, GSEA also showed a strong positive correlation among genes regulated by the MEK inhibitor PD0325901 in the MPNST cell line 90-8TL (13) and genes regulated by HuR silencing in ST88-14 MPNST cells (Supplemental Figure 9C). In addition, several signatures of downstream effectors of these pathways, including the RB and E2F TFs, were inhibited by HuR silencing (Figure 8, A and B). These effectors are frequently dysregulated in cancer and play an important role in cell cycle regulation (22).

To examine the specific function of HuR in controlling these tumorigenic pathways, we examined whether HuR was bound to and regulated expression of different components of these pathways (Figure 8C). By RIP-qPCR analysis, we found that HuR was bound to *CCND1*, *CCND2*, *CDK2*, *CDK6*, *p27*, *E2F1*, *E2F2*, and *E2F3* mRNAs (Figure 8D). In both the ST88-14 and STS-26T cell lines, we found that silencing HuR strongly reduced the levels of several proteins encoded by these mRNAs, accordingly to the role of HuR in promoting mRNA stability and translation (Figure 8E and Supplemental Figure 9D). However, in specific cases, HuR can also inhibit protein translation (16), as described for *p27* (37). Here, similarly, we found that HuR depletion led to an increase in p27 protein level. p21 protein levels were also increased, not as a direct target of HuR, but possibly as one of the key proteins increasing in abundance during cell senescence.

Collectively, our results suggest that HuR may play a direct role in driving cell cycle progression via E2F TFs in MPNST cells by acting at several levels: (a) by enhancing the expression of specific cyclins and CDKs to promote phosphorylation of RB1 to release E2F TFs, (b) by repressing production of p27, an inhibitor of CDKs, and (c) by directly regulating the levels of E2F TFs.

### HuR activates a key Wnt/β-catenin oncogenic program in MPNSTs.

Using the Sleeping Beauty forward genetic screen, signaling through the canonical Wnt/β-catenin pathway was recently identified as a key genetic driver of MPNST tumorigenesis (33, 34). The canonical Wnt pathway is activated after Wnt stimulation, leading to an inhibition in degradation of β-catenin, which then enters the nucleus and binds to a member of the lymphoid-enhancing factor 1/T cell factor (LEF1/TCF) family and other transcriptional cofactors including BCL9 and Pygopus to regulate the expression of target genes involved in diverse cellular processes (38). We found that HuR silencing led to a downregulation of *MYC*, *SOX9*, and *AURKA/B* mRNAs (Supplemental Table 4), which have previously been identified as downstream targets of the Wnt/β-catenin pathway in different cell systems (39–41). Here, we confirmed that these genes were also downstream target genes in MPNST cells by silencing β-catenin using specific shRNAs in the ST88-14 and STS-26T cell lines (Supplemental Figure 10, A and B).

**Figure 9:**
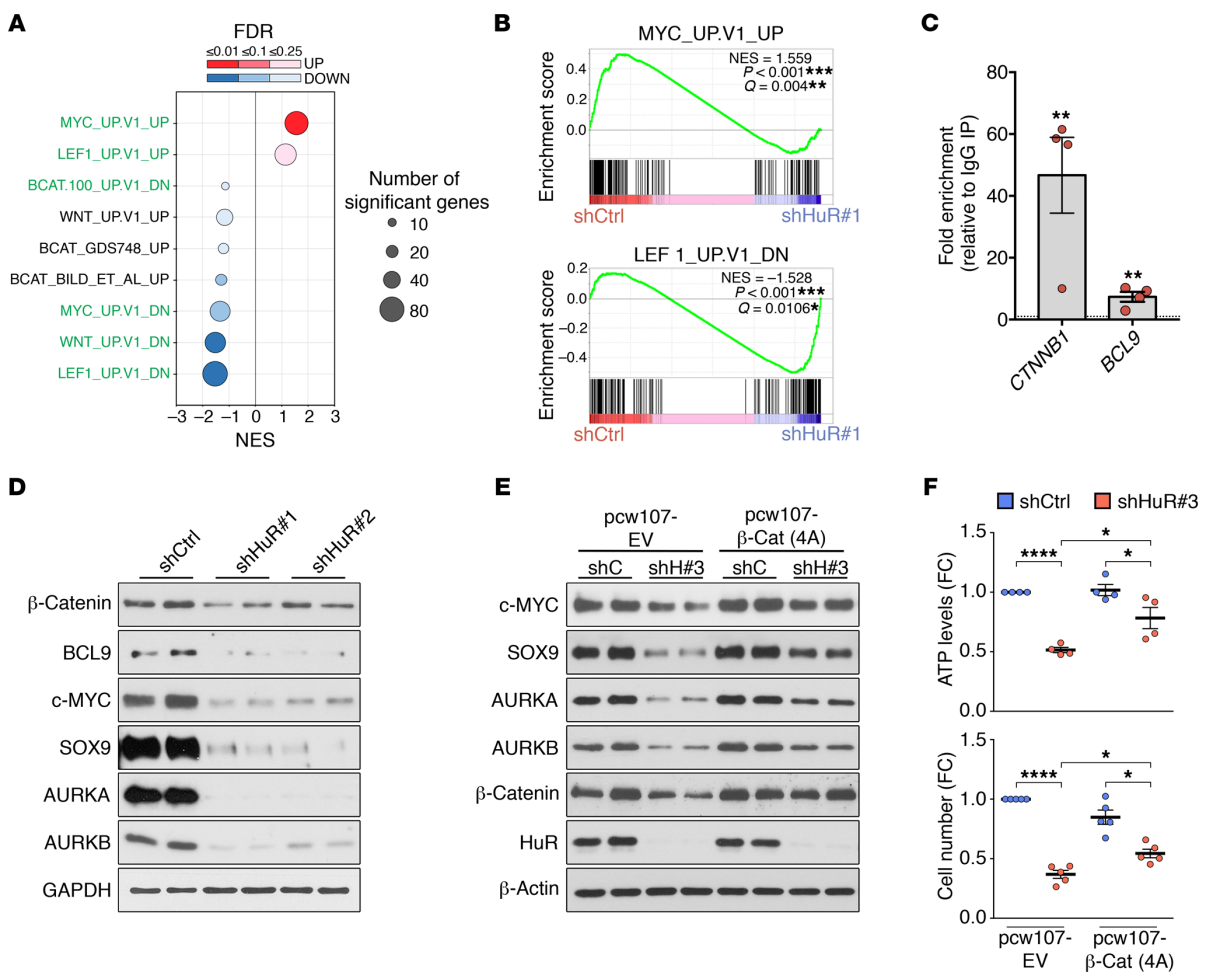
HuR activates key oncogenic programs by regulating the Wnt/β-catenin pathway. (**A**) Compendium of normalized enrichment scores (NES) of target gene sets associated with the Wnt/β-catenin pathway after GSEA analysis of HuR-silenced ST88-14 MPNST cells (Supplemental Tables 5 and 6). Notably, there is general positive correlation in the activation of the pathways (highlighted in green) in shCtrl-infected cells. Circles denotes the number of enriched genes in each category, and colors represent FDR *Q* values. (**B**) GSEA plots showing enrichment of a MYC-induced target gene set in shCtrl cells, and a LEF1-repressed target gene set in shHuR#1-infected cells. (**C**) RIP-qPCR analysis showing binding of HuR to *CTNNB1* and *BCL9* in 4 MPNST cell lines (ST88-14, 90-8, S462, STS-26T). Data are normalized to control IgG IPs and are presented as mean ± SEM, 2-tailed unpaired Student’s *t* test. (**D**) Representative Western blots showing a general downregulation of Wnt/β-catenin pathway components, including key oncogenic downstream regulators, after HuR silencing in ST88-14 MPNST cells. Technical duplicates are shown, and similar results were obtained in 3 independent experiments. (**E**) Representative Western blots showing that lentivirus-based expression of constitutively active β-catenin 4A mutant (harbors alanine substitutions at S33, S37, T41, and S45, preventing its degradation) [pcw107-β-Cat (4A)] partially blocks the downregulation of the key downstream regulators c-MYC, SOX9, AURKA, and AURKB by HuR silencing (shH#3) in ST88-14 MPNST cells. Technical duplicates are shown, and similar results were obtained in 3 independent experiments. (**F**) Ectopic expression of constitutively active β-catenin 4A mutant partially blocks the effects of HuR silencing on cell numbers and ATP levels in ST88-14 MPNST cells. Data are normalized to shCtrl + pcw107-EV cells and are presented as mean ± SEM. Each data point represents 1 independent experiment; 1-way ANOVA with Tukey’s multiple-comparisons test. *P < 0.05; **P < 0.01; ****P < 0.001.

In line with this set of targets, GSEA analysis showed that several signature data sets associated with this pathway were significantly downregulated by HuR silencing (Figure 9, A and B, and Supplemental Tables 5 and 6). RIP analysis showed that HuR associated with *CTNNB1* mRNA, which encodes β-catenin as well as *BCL9* mRNA, which we also identified as a target of HuR from our RIP-Chip analyses (Supplemental Table 2 and Figure 9C). A functional role of β-catenin in controlling MPNST cell growth and viability had already been shown using shRNA-mediated silencing of CTNNB1 (34).

HuR silencing led to a notable reduction in the levels of β-catenin and BCL9 in ST88-14 (Figure 9D) and STS-26T cells (Supplemental Figure 10C), showing that HuR controlled protein production from these mRNAs. HuR depletion also significantly lowered the levels of the key downstream targets, including c-MYC, SOX9, AURKA, and AURKB, further supporting a role for HuR in regulating Wnt/β-catenin–mediated gene transcription. To confirm these results, we examined the capacity of ectopically expressing a degradation-resistant form of β-catenin to abrogate the effects of HuR silencing in MPNST cells. We found that constitutive expression of the β-catenin mutant partially blocked the effects of HuR silencing on downregulation of the downstream Wnt pathway targets in ST88-14 MPNST cells (Figure 9E). In line with this, although silencing HuR still reduced ATP levels and cell number in cells overexpressing the β-catenin mutant, there was a significant recovery of these parameters in comparison with *HuR*-silenced cells expressing the empty vector plasmid (Figure 9F). Similar results were obtained in the sporadic MPNST STS-26T cell line (Supplemental Figure 10, D and E).

Conversely, overexpression of BCL9 using a lentiviral vector failed to rescue the suppression of MPNST growth induced by HuR silencing (data not shown), suggesting that regulation of BCL9 is not central to the biological consequences elicited by HuR in MPNST cells. Our results above also show that a number of key oncogenic TFs, including c-MYC, SOX9, or E2Fs, were downregulated by HuR silencing. Similarly, here, we found no major effect on MPNST growth when the expression of these TFs was restored using lentiviral overexpression constructs after HuR silencing (Supplemental Figures 11 and 12).

These data suggest that there is hierarchical prioritization for HuR function in MPNSTs: HuR primarily regulates the expression of key master regulators (e.g., β-catenin, YAP/TAZ), which in turn control the production of cell cycle proteins and TFs.

### HuR regulates a core transcriptional circuitry in MPNST cells by controlling expression of BRD proteins.

Deregulation of the epigenome has also emerged as an important component of the pathogenesis of MPNSTs. In particular, 2 recent studies pointed to a role of bromodomain and extraterminal domain (BET) proteins in the activation and maintenance of an aberrant transcriptional program (13, 32). BET proteins, including bromodomain-containing protein 2 (BRD2), BRD3, and BRD4, bind to hyperacetylated lysines in promoter/enhancer regions, and subsequently recruit cofactors to control transcription of oncogenic drivers, such as MYC and E2F (42).

**Figure 10:**
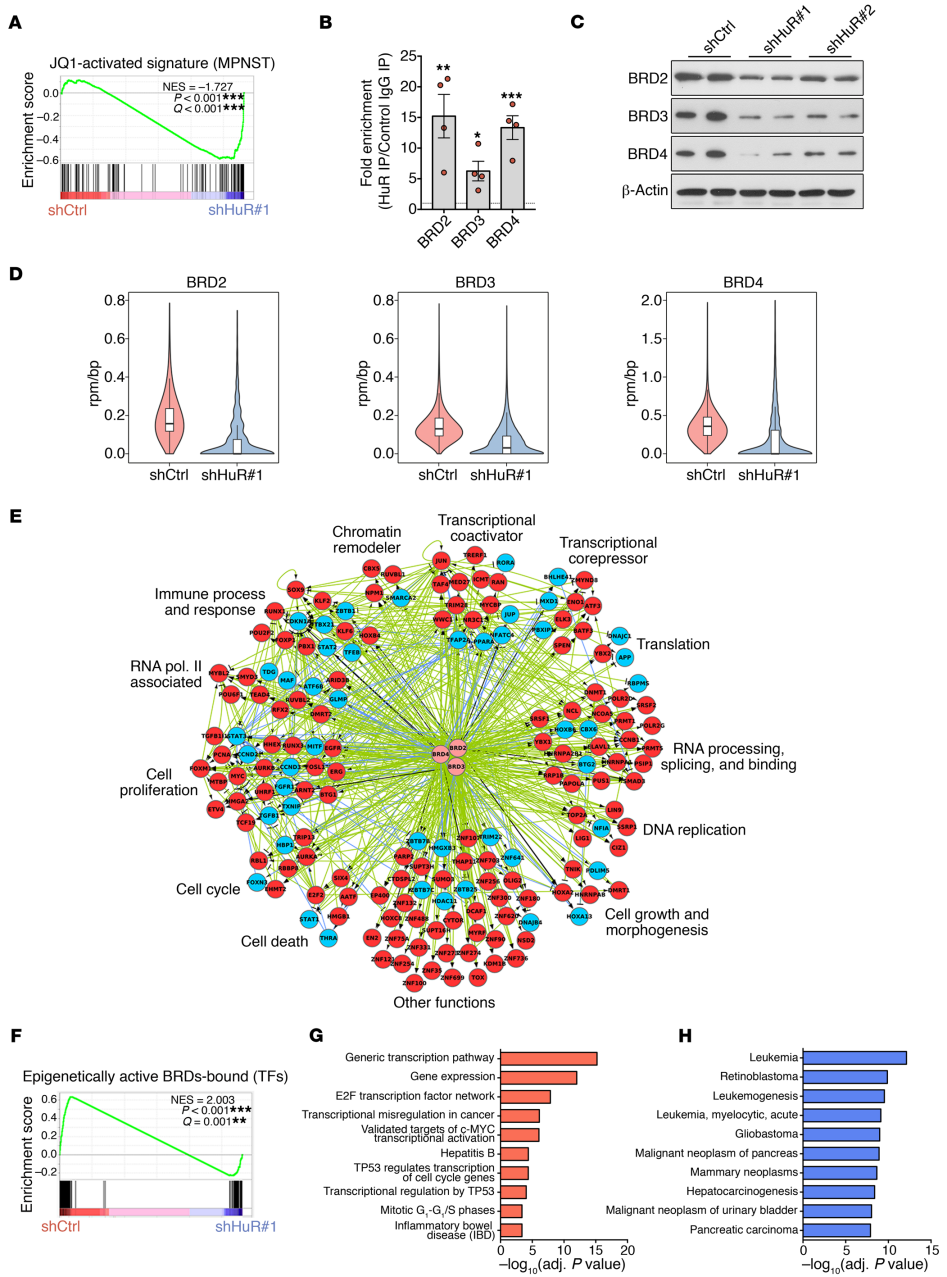
HuR regulates a core transcriptional circuitry in MPNST cells. (**A**) GSEA plots showing enrichment of genes upregulated in MPNST cells by JQ1 treatment (fold change >1.5; adjusted *P* value < 0.05) (13) and shHuR#1-infected ST88-14 MPNST cells. (**B**) RIP-qPCR analysis showing binding of HuR to *BRD2*, *BRD3*, and *BRD4* in 4 MPNST cell lines (ST88-14, 90-8, S462, STS-26T). Data are normalized to control IgG IPs and are presented as mean ± SEM, 2-tailed unpaired Student’s *t* test. (**C**) Representative Western blots showing a downregulation of BRD proteins after HuR silencing in ST88-14 MPNST cells. (**D**) Violin plot showing the distributions of BRD2, BRD3, and BRD4 ChIP-Seq signal at enriched regions in shCtrl-infected and shHuR#1-infected ST88-14 MPNST cells. The *y* axis shows BRD ChIP-Seq signal in units of reads per million (rpm)/bp. The loss of BRD occupancy at BRD-enriched regions after HuR silencing is highly significant (BRD2, *P* = 4.44 × 10^–16^; BRD3, *P* = 1.332 × 10^–15^; BRD4, *P* = 1.332 × 10^–15^; Welch’s *t* test). (**E**) Gene regulatory networks among differentially expressed, epigenetically active TFs that are either direct or indirect targets of BRD proteins. Red and blue circles represent differentially upregulated and downregulated TFs, respectively, in shCtrl-infected cells. Light green edges indicate regulatory interactions unique to shCtrl cells, light blue edges indicate those unique to shHuR#1-infected cells, and black edges indicate those present in both phenotypes. Pointed arrows indicate activation, and blunted arrows indicate inhibition. Functional categories are based on Gene Ontology Biological Processes. (**F**) GSEA plot showing strong enrichment of epigenetically active TFs that are either direct or indirect targets of BRD proteins in shCtrl-infected compared with shHuR#1-infected ST88-14 MPNST cells. (**G** and **H**) ToppGene analysis of HuR-regulated TF network in MPNSTs (red circles from **E**), as classified according to pathway categories (**G**) or disease (**H**).

In MPNST cells, a small-molecule inhibitor of the BET proteins, JQ1, blocks proliferation and can induce apoptosis in MPNST cells in vitro and in vivo, highlighting the importance of these proteins in MPNST pathogenesis (13, 32). Using GSEA analysis, we found a striking enrichment of genes activated by JQ1 treatment in MPNST cells (13), and genes upregulated by HuR silencing in our data set (Figure 10A). Furthermore, in line with our RIP-Chip analyses, where we found enrichment of *BRD2* mRNA in HuR-bound fractions (Supplemental Table 2), we found that mRNAs encoding all 3 BET family members, BRD2, BRD3, and BRD4, were highly enriched in HuR fractions in RIP-qPCR analyses of MPNST cell lines (Figure 10B). HuR silencing strongly reduced all 3 BET proteins in both the ST88-14 and STS-26T cell lines, suggesting a role for HuR in promoting the stability and/or translation of the corresponding mRNAs (Figure 10C and Supplemental Figure 13A). A functional role of BRD4 in controlling cell growth and viability in MPNST cells had already been shown using shRNA-mediated silencing (13, 32). Here, we found that silencing BRD2 using 2 distinct *BRD2*-specific shRNAs also led to a reduction in cell growth and viability (Supplemental Figure 13, B–D), underscoring the importance of this transcriptional regulator in MPNSTs.

Key recent studies have shown that JQ1 treatment in melanoma and multiple myeloma cells depletes BRD2 and BRD4 from promoter and enhancer regions of the genome, and this was associated with the disruption of transcriptional programs in these cells (43, 44). To gain mechanistic insight into the role of HuR in mediating the function of BET proteins in MPNST cells, we investigated changes in BET occupancy on a genome scale by ChIP-Seq in ST88-14 cells after HuR silencing. We found that HuR silencing significantly reduced genome-wide BRD2, BRD3, and BRD4 occupancy by approximately 60%, approximately 50%, and approximately 45%, respectively (Figure 10D).

Next, to examine the functional effects of the BETs’ occupancy on transcriptional regulation, we generated a chromatin landscape in ST88-14 cells using H3K4me3 to identify promoters, H3K4me1 to identify enhancers, and H3K27ac to identify active promoter/enhancer regions. Using this information, we performed network analysis to generate a network of transcriptional regulators that are targets of BRD proteins and regulated by HuR expression. In brief, we identified BRD-bound epigenetically active (active promoters and enhancers) and differentially regulated TFs in control and HuR-silenced cells (Figure 10E). It is apparent from this analysis that there is a considerable number of transcriptional regulators in MPNST cells that are associated with BRD proteins and that control key processes, such as proliferation. Importantly, we found that a substantial proportion of these transcriptional regulators were especially sensitive to HuR inhibition, which depleted BRD proteins from their promoter/enhancer regions and downregulated their expression (Figure 10E, red circles). Thus, GSEA analysis of BRD-bound transcriptional regulators demonstrated a significant enrichment of genes activated in control cells and repressed by HuR silencing (Figure 10F). Gene enrichment analysis by ToppGene suite (45) identified these repressed regulators as being overrepresented in different oncogenic pathways, including E2F and MYC TF networks, proliferation, and TP53-regulated pathways, and for different cancers (Figure 10, G and H).

Collectively, these data demonstrate that BRD proteins are enriched at promoter/enhancer regions of key transcriptional regulators in MPNST cells, and that a reduction in their levels and genome occupancy by HuR silencing suppresses this oncogenic transcriptional circuitry.

## Discussion

Cancer arises from multiple genetic lesions that lead to aberrant gene expression programs, which are increasingly being recognized as fundamental for the acquisition, development, and maintenance of cancer phenotypes. Compelling recent evidence shows that cancer cells can develop absolute dependencies on discrete molecular regulators — out of the thousands of human proteins that contribute to control of gene expression — that drive these dysregulated transcriptional programs. There is nowadays an intense search for these key regulators through focused mechanistic studies, since they represent attractive targets for effective and enduring therapies in cancer (9, 10).

In this study, we present evidence that MPNST cells exhibit an exceptional reliance on the RNA-binding protein HuR for their abilities to survive, proliferate, and disseminate. HuR inhibition prevented the formation of tumors in xenograft models, and even induced a striking regression of tumor volume in established tumors. Furthermore, HuR strongly promoted the metastatic capacity of MPNST cells, one of the worst prognostic features of this cancer. We propose that HuR-regulated transcriptomes promote the survival and adaptation strategies that allow the disseminated cancer cells to survive in the circulation and migrate to, extravasate, and thrive in incompatible foreign microenvironments of distant tissues. Consistent with these results, we found that HuR regulated essential biological capabilities of MPNST cells, including cell cycle progression and sustained proliferation, resisting cell death in stress conditions and enabling replicative immortality. Notably, the functional consequences of HuR inhibition were largely comparable in a representative panel of 4 MPNST cell lines obtained from patients with different NF1 status, even though these exhibit heterogeneous cellular growth rates and alterations in expression of a number of cell cycle proteins (24). This lack of major differences, functional or mechanistic, between NF1-derived and sporadic MPNSTs after HuR silencing supports the view that relatively similar molecular mechanisms are involved in the pathogenesis of MPNSTs, and that HuR can be a broad target for MPNSTs, irrespective of NF1 status (1).

Mechanistically, our RIP-Chip and transcriptomics data showed a global dysregulation of several signaling pathways and molecular regulators in these cancer cells, including the HIPPO-YAP/TAZ, PI3K/AKT/mTOR, RAS/RAF-MEK-ERK, and Wnt/β-catenin pathways and bromodomain regulation of gene transcription, while our focused analyses revealed that regulation by HuR of specific components of these pathways likely led to the aberrant signaling. Several of these pathways, including Wnt/β-catenin, seem to be specific to MPNSTs, since we find that HuR cannot regulate them in normal Schwann cells (data not shown). We posit that this capacity of HuR to simultaneously control several essential molecular regulators that operate in these cancer cells explains in large part the striking effects of HuR inhibition in MPNST cells. All these pathways have been shown to contribute to MPNST pathogenesis in seminal studies from several laboratories using various genetically engineered mouse models or culture systems (1, 2, 6, 13, 32–34, 46). However, genetic or pharmacological inhibition of these targets individually has, in general, been modestly effective and largely cytostatic. Instead, concurrent targeting of different pathways has proved more potent. Thus, combined inhibition of YAP/TAZ and PDGFR signaling activity (6), BRD proteins and MEK activity (13), or the mTOR and Wnt/β-catenin pathways (34) is strongly synergistic in blocking tumor growth, and can even induce apoptosis.

These observations strongly support the view that parallel and redundant pathways control the oncogenic traits in MPNST cells, a phenomenon that has been associated with the emergence of therapeutic resistance in cancer cells (10, 21). This is particularly true for targeted therapies against kinase-mediated signaling cascades, which are organized in a linear and hierarchical manner, with different receptor tyrosine kinases at the top, activating a reduced number of kinase signaling cascades, including RAS/RAF-ERK, PI3K/AKT, and JAK/STAT, that operate in parallel but that can crosstalk with one another (10). This linear and redundant architecture facilitates bypassing of a signaling pathway after its inhibition for another, thus blocking the therapeutic effects of the drugs. This paradigm is also consistent with the disappointingly poor results obtained in phase I and II clinical trials targeting individual components of kinase cascades in MPNST patients. Because of the superior results obtained from additive effects of cotargeting multiple pathways, current clinical trials are more focused on combination drug therapy (1, 25). Our results strongly argue that targeting HuR could be equally as effective as the combined treatments, as we find that HuR inhibition potently suppresses several of the oncogenic signals in MPNSTs, leading to profound cytostatic and cytotoxic effects on the growth, as well as the metastatic capacity, of tumors.

Strikingly, we find that targeting HuR can both prevent the formation of tumors and metastatic nodules, and lead to shrinkage of fully established tumors and metastases. Thus, therapeutic intervention could be equally effective in newly diagnosed or recurrent tumors, which has particularly appealing translational importance, since timing of intervention and stage of disease have been raised as possible causes of the negative results in clinical trials in MPNSTs. However, even though our promising data on pharmacological inhibition of HuR could represent a viable therapy for patients with MPNSTs, it is still unclear whether the preclinical efficacy observed in this study using cell lines and mice can be translated to the clinic. Whether HuR is a valid therapeutic target in MPNSTs warrants direct investigation.

Our ChIP-Seq data reveal a large network of transcriptional regulators operating in MPNST cells that are associated with key biological functions. Notably, we find that this network is highly sensitive to levels of HuR, which can regulate expression of key components of this network via modulation of BRD protein levels and/or signaling pathways. An essential role for BRD proteins in regulating gene expression has also been shown in several malignancies, including MPNSTs, and interestingly, we find that HuR silencing in MPNSTs phenocopies to a large extent treatment with the BET inhibitor JQ1, at least in terms of gene expression profiles. In addition, the displacement of BRD proteins on promoter/enhancer regions of active cancer-related genes by JQ1 in hematological malignancies and melanoma (42–44) is similar to the reduced promoter/enhancer occupancy on transcriptional regulators in MPNSTs that we found after HuR silencing. Thus, our data suggest that dysregulation of the transcriptional program in MPNSTs by HuR silencing could be in large part mediated by the influence of HuR on the MPNST transcriptional network.

Remarkably, we found that HuR depletion led to strong suppression of MYC and E2F levels that was accompanied by a highly significant reduction in the abundance of MYC- and E2F-transcribed gene sets, pointing to a general suppression of transcription at E2F- and MYC-driven targets by HuR inhibition. These proto-oncogenic TFs are among the most important drivers of tumorigenesis, as they regulate cell growth, proliferation, apoptosis, and metabolic pathways (22, 23), and represent some of the important targets for cancer therapy, although efforts to directly target these TFs have proved unsuccessful so far (9). Our work could thus have far-reaching implications for cancer therapy, since it suggests an alternative strategy to effectively target these TFs. MYC regulation also represents one of the best examples of the “multifunctional” capacity of HuR in driving expression of particular oncoproteins. MYC levels are regulated by major growth-regulatory and oncogenic signaling pathways, including the Wnt/β-catenin, JAK/STAT, and Notch pathways that induce *MYC* transcription and the mTOR pathway that increases the efficiency of *MYC* mRNA translation, together with function of PI3K and RAS signaling and AURKA/B that increase the stability of MYC protein (22, 47). The striking downregulation of MYC levels in MPNST cells by HuR inhibition could potentially be mediated by a combinatorial action at several of these nodes of regulation, which are themselves under strong HuR influence. Similarly, E2F levels/function in MPNST cells could be determined by HuR via its effect on multiple regulatory mechanisms, including cyclin-CDK expression, RB phosphorylation, and *E2F* transcription/translation.

RBPs play a central role in regulation of gene expression, and thus it is not surprising that their dysregulation has been linked to several human diseases, including neurological disorders and cancer (48, 49). These dynamic regulators can bind to and regulate thousands of functionally related genes, regulating every hallmark of cancer cells. Recent detailed studies have shown that RBPs are rapidly emerging as key oncogenic drivers in a variety of malignancies (48, 50–53). In this study, we show that the RBP HuR has pleiotropic functions in MPNSTs, driving tumor growth and metastasis by, quite strikingly, influencing almost all key signaling pathways and regulators discovered in MPNSTs so far, including the PI3K/AKT/mTOR, RAS/RAF-MEK-ERK, Wnt/β-catenin, and HIPPO-YAP/TAZ pathways, and transcriptional regulators including SOX9, AURKA/B, and BRD proteins, as well as the proto-oncogenic TFs MYC and E2Fs. Over 1500 RBPs have been described, and several of them are dysregulated in MPNSTs (data not shown). It would be interesting to examine their biological and mechanistic functions in MPNSTs, and whether they have broad functions similar to those of HuR or regulate distinct pathways.

This function of HuR in establishing the highly intricate regulatory networks operating in MPNSTs to coordinate multiple cancer hallmark traits supports a “master” regulatory function for HuR in MPNSTs. Thus, by elevating HuR levels, MPNST cells have elaborated an adaptive mechanism to amplify and regulate key oncogenic signals and modulate transcriptional programs to confer a competitive advantage to these cancer cells, promoting MPNST growth and metastatic spread (see Graphical Abstract).

## Methods

### Cell culture.

Human MPNST cell lines S462, STS-26T, ST88-14, and 90-8 and an immortalized normal human Schwann cell line (iHSCλ2) were obtained from Nancy Ratner (Cincinnati Children’s Hospital Medical Center) (24, 34). Immortalized plexiform neurofibroma–derived Schwann cells (ipNF SC) were purchased from ATCC (ATCC CRL 3390). The cells were maintained in DMEM (Thermo Fisher Scientific) supplemented with 10% (vol/vol) FBS (Thermo Fisher Scientific) and 1% (vol/vol) antibiotic/antimycotic (Thermo Fisher Scientific) under standard conditions of culture in 37°C and 5% CO_2_.

Human Schwann cells were isolated from the sural nerves of donors with informed consent (see below). Withdrawn nerves were stripped of their epineurium, and fascicles were separated from the remaining interfascicular epineurium. Fascicles were cut into 2-mm-long pieces and incubated in humidified conditions and 10% CO_2_ for 7–14 days in DMEM (Gibco) with 10% FBS (MilliporeSigma), 500 U/mL penicillin/streptomycin (Gibco), and 2.5 mg/mL amphotericin (Gibco). Fascicles were then digested in DMEM with 10% FBS, 500 U/mL penicillin/streptomycin, 0.8 U/mL dispase grade I (MilliporeSigma), and collagenase type 1A 160 U/mL (MilliporeSigma). The resulting Schwann cells were then amplified on plates coated with 0.1 mg/mL poly-l-lysine in DMEM with 20% FBS, 100 U/mL penicillin/streptomycin, 0.5 mM forskolin (MilliporeSigma), 2.5 mg/mL amphotericin, 2.5 mg/mL insulin (MilliporeSigma), 10 nM β1-heregulin (MilliporeSigma), and 0.5 mM IBMX (MilliporeSigma). Amplified Schwann cells were plated in DMEM with 10% FBS in the absence of β1-heregulin for at least 24 hours before analysis.

### Lentivirus preparation and infection of MPNST cells.

Lentiviral particles (Supplemental Table 7) were produced in HEK293FT cells. For single-infection experiments, MPNST cells were incubated with different lentiviral particles together with 8 μg/mL Polybrene (Merck) for enhancing infection efficiency. Selection of infected cells was started 48 hours after infection with puromycin (4 μg/mL) for 2 days in supplemented growth medium or with geneticin (G418 sulfate) (Thermo Fisher Scientific) at 50 μg/mL for 5 days in growth medium without antibiotic/antimycotic. For the inducible knockdown plasmids, after infection and selection, cell lines were treated with 0.5 μg/mL doxycycline every 24 hours for 3 days. Infection efficacy was tested by Western blot and quantitative reverse transcriptase PCR analyses. For rescue experiments, cells were first infected with control or β-catenin–overexpressing plasmids and selected with puromycin, and then further infected with shHuR#3 lentiviral particles followed by selection with geneticin, as above. After infection and selection, MPNST cells were replated at specific densities for the different functional assays. All assays were conducted in triplicate, with at least 3 independent experiments performed for each assay.

### Small-molecule HuR inhibitors.

For culture studies, MPNST cell lines were treated with the following small-molecule HuR inhibitors for 48 hours: (a) MS-444 (29), obtained from Philipp Krastel (Novartis Institutes for Biomedical Research), reconstituted in DMSO and used at a final concentration of 10 μM; (b) pyrvinium pamoate (MilliporeSigma), reconstituted in DMSO and used at a final concentration of 2 μM; and (c) tanshinone mimic 6N (TM-6N) (31), obtained from Alessandro Provenzani (University of Trento), reconstituted in DMSO and used at a final concentration of 10 μM. 0.1% DMSO was used in control cultures.

For in vivo xenograft studies, 1 × 10^6^ STS-26T cells mixed in 1:4 PBS/Matrigel were injected subcutaneously in the left and right back flanks of mice, respectively, under standard procedures and tumors allowed to grow up to 100 mm^3^ average volume, as described above. The mice then received i.p. injections of MS-444 (25 mg/kg) dissolved in PBS/5% *N*-methyl pyrrolidine (NMP) (MilliporeSigma) or vehicle control every 48 hours for a further 10 days.

For lung metastasis studies, 1 × 10^6^ STS-26T cells were injected in the lateral tail vein of mice and left for 2 weeks to allow basal formation of lung metastases, as described above. The mice then received i.p. injections of MS-444 (25 mg/kg) dissolved in PBS/5% NMP (MilliporeSigma) or vehicle control every 48 hours for a further 2 weeks.

All the microarray, RNA-Seq, and ChIP-Seq data were deposited in the NCBI Gene Expression Omnibus database (GEO GSE120687).

### Statistics.

All analyses were done using Microsoft Excel or GraphPad Prism 6.00 (www.graphpad.com). Data are shown in dot plots or histograms as mean ± SEM. A *P* value of less than 0.05 is deemed statistically significant. Statistical analysis was performed by 2-tailed unpaired Student’s *t* tests, Mann-Whitney *U* test, 1-way ANOVA with post hoc analysis by Tukey’s multiple-comparisons test, 2-way ANOVA with Tukey’s multiple-comparisons test, or as indicated. Quantifications were performed from a minimum of 3 experimental groups.

### Study approval.

For animal studies, xenograft or experimental lung metastasis experiments were approved by the Biosafety and Welfare Committee (BAWC) at CIC bioGUNE, and carried out following the ethical guidelines and recommendations from the Association for Assessment and Accreditation of Laboratory Animal Care International (AAALAC).

For human studies, patient samples were obtained in accordance with the ethical standards of the institutional and/or national research committee and with the 1964 Helsinki declaration and its later amendments or comparable ethical standard:

(a) Frozen human normal nerves (*n* = 5), neurofibromas (*n* = 12), and MPNSTs (*n* = 15) were obtained from the Stanmore Musculoskeletal Biobank, a satellite of the University College London (UCL)/University College London Hospitals (UCLH) Biobank (Human Tissue Authority License 12055), which was approved by the National Research Ethics Committee (NREC) (reference 15/YH/0311). This specific study was approved by the NREC-approved UCL/UCLH Biobank Ethical Review Committee (reference EC17.14).

(b) Peripheral nerves for Schwann cell culture were retrieved from donors after consent under the National Health Service Blood and Transplant (NHSBT) research study entitled “Collection of peripheral nerves for control cells for brain tumor treatment research,” NHSBT study 61. The full research project title was “Identifying and validating molecular targets in low grade brain tumors” (Research Ethics Committee [REC] number 14/SW/0119; IRAS reference 153351).

## Author contributions

MPI, EPA, MIL, ABM, MTC, DM, LVV, LMC, and NB carried out in vitro and in vivo analyses. AM Aransay, DMA, JJL, JLL, AM Ascensión, DG, and MJAB carried out sequencing and bioinformatics analysis. SO and ADS supervised network analysis. NMM, MGL, JDS, DFR, VGDJ, NMC, AC, RB, and MLMC provided technical support and discussion. LL, DBP, COH, EE, HI, AI, PP, ES, CL, AMF, RDC, MG, CWS, AP, PK, PS, and NR supplied human samples and reagents. MPI, DBP, MG, AC, AM Aransay, and MVR edited the manuscript. AW and MVR directed the study and contributed to data acquisition and analysis. AW wrote the manuscript. Order of authorship for first coauthors and co–senior authors was determined by relative contribution to data acquisition and design of the research study.

## Supplementary Material

Supplemental data

Supplemental Table 4

## Version 1 04/21/2020

In-Press Preview

## Version 2 06/15/2020

Electronic publication

## Version 3 07/01/2020

Print issue publication
