# Supplementary material for: HuR/ELAVL1 drives malignant peripheral nerve sheath tumor growth and metastasis
Source: J Clin Invest. 2020 Jun 15;130(7):3848–64. doi: 10.1172/JCI130379 (PMC7324187; doi:10.1172/JCI130379)
Supplement: Supplemental data [file jci-130-130379-s388.pdf]

HuR/ELAVL1 drives Malignant Peripheral Nerve Sheath Tumour growth and metastasis

Authors and affiliations.
Marta Palomo-Irigoyen[1#], Encarni Pérez-Andrés[1#], Marta Iruarrizaga-Lejarreta[1#], Adrián Barreira-Manrique[1], Miguel Tamayo-Caro[1], Laura Vila-Vecilla[1], Leire Moreno-Cugnon[1], Nagore Beitia[1], Daniela Medrano[1], David Fernandez-Ramos[1,2], Juan José Lozano[3], Satoshi Okawa[4,5], José L. Lavín[1], Natalia Martín-Martín[1,6], James D. Sutherland[1], Virginia Guitiérez de Juan[1,2], Monika González-Lopez[1,2,], Nuria Macías-Cámara[1,2], David Mosén-Ansorena[1], Liyam Laraba[7], C. Oliver Hanemann[7], Emanuela Ercolano[7], David B. Parkinson[7], Christopher W. Schultz[8], Marcos J. Araúzo-Bravo[9,10], Alex M. Ascensión[9], Daniela Gerovska[9], Haizea Iribar[11], Ander Izeta[11], Peter Pytel[12], Philipp Krastel[13], Alessandro Provenzani[14], Pierfausto Seneci[15], Ruben D. Carrasco[16], Antonio Del Sol[1,4,10,17], María Luz Martinez-Chantar[1,2], Rosa Barrio[1], Eduard Serra[6,18], Conxi Lazaro[6,19,20], Adrienne M. Flanagan[21,22], Myriam Gorospe[23], Nancy Ratner[24], Ana M. Aransay[1,2], Arkaitz Carracedo[1,6,10,25], Marta Varela-Rey[1,2*], Ashwin Woodhoo[1,10*]

[#]Contributed equally to this work
[*] Co-Senior authors

Corresponding author: Ashwin Woodhoo, Nerve Disorders Laboratory, Center for Cooperative Research in Biosciences (CIC bioGUNE), Basque Research and Technology Alliance (BRTA), Bizkaia Technology Park, Building 801A, 48160 Derio, Spain. awoodhoo@cicbiogune.es; Tel: +34-944-061312; Fax: +34-944-061301.

SUPPLEMENTAL DATA

1) Supplemental Materials and Methods

2) Supplemental Figures

3) Supplemental Tables

4) Supplemental References

SUPPLEMENTAL MATERIALS AND METHODS

ATP assay

Transduced cells were seeded in triplicate in 96-well plates (1,000 cells per well) and 2 days after plating, ATP levels were measured with the ATPlite Luminescence Assay System (Perkin Elmer) following the manufacturer' s instructions. Luminescence values were collected in a Veritas Microplate Luminometer (Turner Biosystems), quantification performed using a standard curve and values normalized with values obtained at 1 day after plating.

Colony formation assay

For colony formation analysis, transduced cells were seeded in triplicate in 12-well plates (150 cells per well) and cultured in supplemented growth medium for up to 10 days. Cells were then washed with PBS, fixed with 10% formalin solution (Merck) and stained with 0.1% (w/v) crystal violet (Merck) in 20% methanol. After gentle rinsing with water, the retained dye was extracted with 10% (v/v) acetic acid and the absorbance measured at 595 nm on a Synergy HT spectrophotometer (Biotek).

Anchorage-independent growth assay

A total of 45,000 MPNST cells were mixed in culture medium (37 ^oC) containing 0.3% low-melting-point agar (Fermentas) and then seeded in triplicate in a 6-well plate on top of a layer of 0.6% agar (Thermo Fisher Scientific). Cells were allowed to grow for 3-4 weeks in culture, with supplementation of complete growth medium every week until colonies were visible by eye. The soft agar plates were scanned (HP Scanjet G4050, Hewlett Packard) and colonies (composed of at least 20 cells) quantified using the ImageJ software (https://imagej.nih.gov/ij/).

Bromodeoxyuridine (BrdU) incorporation assay

To assess DNA synthesis and proliferation, 5,000 transduced cells were seeded in triplicate onto glass coverslips in 24-well plates, and cultured in supplemented growth medium for 2 days. Cells were incubated with 10 µM BrdU (Merck) for the last 2 h of culture before fixation with 2 M hydrochloric acid and immunolabeling with anti-BrdU antibody (Roche) and nuclear staining with 50 ng/ml of DAPI (Merck). The number of BrdU+ cells were counted and expressed as a percentage of total number of DAPI+ cells.

Cell growth assay

Transduced cells were seeded in triplicate in 24-well plates (10,000 cells per well) and cultured in supplemented growth medium for 2 days before trypsinization and counting using a haemocytometer. Growth was determined relative to the number of cells initially plated (10,000). For culture under growth-limiting conditions, cells were cultured in 2% FBS for 3 days before counting. Log2 of fold growth was determined (negative values indicate cell death).

Cell cycle analysis

Transduced cells were seeded in triplicate on six-well plates (150,000 cells per well) and 2 days after seeding, adherent and non-adherent cells were collected, washed with PBS, and fixed and permeabilized with cold 75% ethanol at –20 ºC overnight. DNA staining was performed with 50 µg/ml propidium iodide (PI) solution (Thermo Fisher Scientific) in the presence of 50 µg/ml RNase A (Macherey-Nagel). Cell cycle was analysed using a FACS CANTO (BD Biosciences) flow cytometer. Data were analyzed using FACSDiva software (BD Biosciences).

Flow cytometer and apoptosis

Transduced cells were seeded in triplicate on six-well plates (150,000 cells per well) and 2 days after seeding, adherent and non-adherent cells were collected and stained with Annexin V–FITC and PI (Immunostep) following the manufacturer's instructions. Cells were fixed with 1% formaldehyde before analysis using a FACS CANTO (BD Biosciences) flow cytometer. Data were analyzed using FACSDiva software (BD Biosciences).

Senescence associated-X-gal staining.

To assess senescence, 10,000 transduced cells were seeded in triplicate onto glass coverslips in 24-well plates, and cultured in supplemented growth medium for 2 days. Cells were fixed and analyzed for SA-β-Gal activity using the Senescence Detection Kit (Calbiochem) following the manufacturer's instructions at 37 ºC overnight or until blue colour developed. Cells were rinsed with PBS and mounted using fluorescent mounting medium (Dako). Pictures were acquired using an optical microscope and β-Gal+ cells were counted and expressed as a percentage of total number of cells.

RNA Isolation and reverse transcription (RT) followed by quantitative (q)PCR analysis

Transduced cells were seeded in triplicate in 6-well plates (150,000 cells per well) and 2 days after seeding (unless otherwise specified) cells were rinsed with PBS followed by total RNA isolation with TRIzol Reagent (Thermo Fisher Scientific) following the manufacturer's instructions. Human cancer samples were extracted with TRIzol Reagent (Thermo Fisher Scientific) following manufacturer's instructions. Total RNA was treated with DNase I (Invitrogen) and cleaned with Genejet RNA Cleanup and Concentration Kit (Thermo Scientific). Up to 1 µg of total RNA was used for cDNA synthesis with M-MLV Reverse Transcriptase (Thermo Fisher Scientific) using random hexamer primers in the presence of RNase out inhibitor (Thermo Fisher Scientific). Amplifications of cDNA were run in a Viia7 Real-Time PCR System (Applied Biosystems) with PerfeCTa® SYBR® Green SuperMixes and FastMixes™ with low ROX reference dye (Quanta Biosciences). Quantification was performed using the $\Delta\Delta C_T$ method. Normalization was performed using *GAPDH* mRNA as a standard. See Supplemental Table 7 for primer sequences.

Western blot analysis

For western blot analysis, transduced cells were seeded in duplicate in 6-well plates (150,000 cells per well) and 2 days after seeding, total proteins were extracted with RIPA buffer [500 ml stock solution: 1.6 mM NaH_2PO_4 (Merck), 8.4 mM Na_2HPO_4 (Merck), 0.1% Triton X-100 (VWR), 0.1 M NaCl (Ambion), 0.1% SDS (Fisher Scientific) and ddH_2O] containing sodium deoxycholate (Merck), 1 mM sodium fluoride, and 1X protease and phosphatase inhibitor cocktail (Roche). For xenograft tumours and human cancer samples, a small piece of tissue was excised and homogenized in supplemented RIPA buffer using the Precellys Homogenizer (Bertin technologies). Protein aliquots (8–20 μg each) were denaturalized in 5X loading buffer [0.25 M Tris, pH 6.8 (VWR), 5% SDS, 2-mercaptoethanol (Merck), 50% glycerol and bromophenol blue powder (Merck)] for 5 min at 95 ^{o}C. Samples were separated on 8%, 11% or 15% tris-glycine SDS-polyacrylamide gels with 1X Tris-Glycine-SDS buffer (Bio-Rad) and transferred to a 0.2-μm nitrocellulose membranes (Amersham) with transfer buffer [Glycine (VWR), Tris (Trizma base, VWR), 20% Methanol (Panreac AppliChem) and ddH_2O]. Membranes were then incubated with relevant primary and secondary antibodies (See Supplemental Table 7), and blots were developed with ECL substrate (BioRad).

Histologic preparation and Immunohistochemistry (IHC)

Tumour xenografts and lungs were dissected out, fixed in 10% formalin solution overnight and embedded in paraffin. Sections (5-μm thick) were dried, deparaffinized, rehydrated and subjected to antigen retrieval and then incubated overnight with primary antibodies. After three washes with PBS, sections were incubated for 1 h with secondary antibodies, followed by EnVision+ System HRP system (Dako) and incubated with peroxidase/diaminobenzidine (DAB) for colour development. The slides were washed in distilled water, counterstained with Haematoxylin, dehydrated and mounted with permanent media. Standard haematoxylin and eosin staining was also performed. For analysis of HuR expression in patient samples, a tissue microarray panel consisting of 7 normal nerve, 76 neurofibromas and 109 MPNSTs (1) was subjected to HuR IHC. Digital images were then acquired with an AXIO Imager A1 microscope (Carl Zeiss AG). The sections were scored in a blinded manner for staining intensity (0–2).

RNA Immunoprecipitation

Immunoprecipitation (IP) protocol of endogenous mRNA-HuR complexes was performed as described by (2,3). For RIP-chip analyses, frozen tissue samples from human cancer panel (n=8 neurofibroma and n=12 for MPNST) were homogenized in polysome lysis buffer [100 mM KCl, 5 mM $MgCl_2$, 10 mM HEPES pH 7.0, 0.5% NP-40, 1 mM DTT, 100 units/ml RNase OUT, 1X Protease Inhibitor Cocktail), incubated for 30 min on ice, and centrifuged at 13 000 rpm, 4 ^{o}C for 30 minutes. 500 µl of lysates were pre-cleared by incubating with 25 µl of protein A-sepharose 4B beads (Merck) and anti-mouse IgG (BD Biosciences) in 1 ml of NT2 buffer [50 mM Tris pH 7.4, 150 mM NaCl, 1

mM $MgCl_2$, 0.05% NP-40] and incubating under rotation for 30 min. The pre-cleared extracts were then divided and incubated with 50 µl of protein A-sepharose 4B beads, pre-coated with anti-HuR or anti-mouse IgG antibodies. After incubation, beads were washed 5 times with 1 ml NT2 buffer and bound RNA recovered after proteinase K digestion (Roche) and phenol chloroform extraction. RNAs were then submitted to the Genomics Analysis Platform at CIC bioGUNE for analysis on HUMAN HT-12 V4 arrays (Illumina). For RIP-qPCR analyses, 4 MPNST cell lines (S-462, STS-26T, ST88-14 and 90-8) were cultured in 15-cm plates until they were 80% confluent. IP was performed essentially as above, and an equal volume of extracted RNA from each sample was then used for cDNA synthesis and analysed by quantitative PCR.

RNA Sequencing and Data Analysis

ST88-14 cells were infected with shControl or shHuR#1 lentivirus, selected with puromycin for 2 days, replated and then RNA isolated 2 days later with TRIzol Reagent (Thermo Fisher Scientific) following manufacturer's instructions. The quantity and quality of the RNAs were evaluated using Qubit dsDNA Assay Kit (Thermo Fisher Scientific) and Agilent RNA Nano Chips (Agilent Technologies), respectively. TruSeq RNA Sample Preparation v2 kit (Illumina Inc.) was used following the TruSeq® RNA Sample Preparation v2 Guide (Part # 15026495 Rev. F). In brief, starting from 500 ng of total RNA, mRNA was purified, fragmented and primed for cDNA synthesis. cDNA first strand was synthesized with SuperScript-II Reverse Transcriptase (Thermo Fisher Scientific) for 10 min at 25 ºC, 15 min at 42 ºC, 15 min at 72 ºC and pause at 4 ºC. cDNA second strand was synthesized with Illumina reagents at 16 ºC for 1 hour. Then, A-tailing and adaptor ligation were performed. Finally, enrichment of libraries was achieved by PCR (30 sec at 98 ºC; 15 cycles of 10 sec at 98 ºC, 30 sec at 60 ºC, 30 sec at 72 ºC; 5 min at 72 ºC and pause at 10 ºC). Afterwards, libraries were visualized on an Agilent 2100 Bioanalyzer using Agilent High Sensitivity DNA kit (Agilent Technologies) and quantified using quantitative PCR with Kapa Library Quantification Kit (Master Mix and DNA Standards, KAPA – Biosystems) and Qubit dsDNA HS DNA Kit (Thermo Fisher Scientific). RNAseq-libraries single-Read sequencing of 50 nucleotides was carried out in a HiScanSQ platform (Illumina Inc.). Reads were trimmed for adapters using cutadapt (4) and aligned to hg38 genome using STAR (5). Quantification in expected counts from genes and isoforms were computed by RSEM (6) using genecode annotation v.26 [https://www.gencodegenes.org/]. We use TMM method to estimate scale factors between samples followed by the voom function in *limma* to convert them into $\log_2$counts per million (logCPM). Finally, differential expression between shControl and shHuR-infected cells were evaluated by LIMMA bioconductor package (7). Genes with a fold change of 2 and FDR < 0.05 were considered as significantly different.

ChIP-Seq and Data Analysis

Chromatin immunoprecipitation was performed essentially as described (8). In brief, ST88-14 cells were infected with shControl or shHuR#1 lentivirus, selected with puromycin for 2 days, replated and then 2 days later cross-linked with 1% formaldehyde for 10 min at RT and reaction quenched with 125 mM glycine for 5 min. The isolated nuclei were resuspended in nuclei lysis buffer and sonicated using a Bioruptor Sonicator (Diagenode). The samples were immunoprecipitated with the appropriate antibodies overnight at 4 ^{o}C. Protein G beads (Thermo Fisher Scientific) were added and incubated for 1 h, and the immunoprecipitates were washed twice, each with low-salt, high-salt and LiCl buffer. The eluted DNA was reverse-crosslinked and purified using PCR purification kit (Qiagen). The quantity and quality of the DNAs were evaluated with Qubit dsDNA HS DNA Kit (Thermo Fisher Scientific) and Agilent High Sensitivity DNA kit (Agilent Technologies), respectively. Sequencing libraries were prepared following TruSeq® ChIP Sample Preparation Guide with the corresponding kit (Illumina Inc.). Input ChIP DNA (5–10 ng) was blunt–ended and phosphorylated. A single 'A' nucleotide was added to the 3' ends of the fragments in preparation for ligation to an adapter that has a single-base 'T' overhang. The ligation products were purified and accurately size-selected by agarose gel electrophoresis. Size-selected DNA was purified and PCR-amplified to enrich for fragments that have adapters on both ends. Resulting libraries were visualized on an Agilent 2100 Bioanalyzer using Agilent High Sensitivity DNA kit (Agilent Technologies) and quantified using Qubit dsDNA HS DNA Kit (Thermo Fisher Scientific, Cat). ChIPseq libraries were single-read sequenced for 51 nucleotides in a HiSeq2500 (Illumina Inc.)

The sequencing data were mapped to the hg38 genome assembly, biological replicates merged and peak calling was performed using Model-based analysis of ChIP-seq (MACS) 2 (9) to identify regions of ChIP-Seq enrichment over background (input) with an enrichment threshold of adjusted p-value < 0.01.

BRD proteins occupancy

The BRD samples were processed using NaviSE (10) with default parameters (bowtie2 aligner with --very-sensitive parameter). In order to find the signal at enriched regions, occupancy was calculated using a sliding window of 50 nucleotides, and counting the number of reads within each window. Values were adjusted to reads per million (rpm)/bp units. Enriched regions were determined as the set of peaks obtained in MACS2 in shControl cell with q-value < 10^-5. The windows lying within the enriched regions were selected and used for the violin plots. The difference in the distributions of BRD4 was compared using a Welch's t test.

Gene Ontology Analyses

Gene ontology analyses for RNA-Seq dataset were performed using Gene Set Enrichment v3.0 (GSEA, http://www.broadinstitute.org/gsea/index.jsp) (11). Gene sets

used were obtained from the Molecular Signatures Database v6.0 (MSigDB, http://www.broadinstitute.org/gsea/msigdb/index.jsp, C1 hallmark gene sets or C6 oncogenic signatures) or were manually curated from published data set. Normalized enrichment score (NES) denotes the degree to which the gene-set is overrepresented at the top or bottom of a ranked list of genes. Genes categorized with negative or positive NES are downregulated or upregulated, respectively. The nominal P value describes the statistical significance of the enrichment score. The FDR q value is the estimated probability that a gene set with a given NES represents a false positive finding. The GSEA summary plots in Figure 2B, 6C and 9A were plotted with Microsoft Excel software, and show upregulated and downregulated gene sets. Circle size is proportional to the core enriched genes, i.e. the subset of members within a gene set that shows statistically significant, concordant differences between two biological states and contribute most to the NES. Gene sets with FDR q values < 0.25 are plotted as a function of NES. Circle colours represent FDR q values. Gene enrichment analysis for transcriptional network was performed using ToppGene suite (https://toppgene.cchmc.org/enrichment.jsp) (12).

Gene Expression datasets

We collected the microarray expression profiles of human normal nerves, neurofibromas and MPNSTs from the GEO public resource (http://www.ncbi.nlm.nih.gov/geo/) and the accession numbers are GSE41747 (13) and GSE14038 (14). RNA-Seq data of control and Lats1/2-deficient Schwann cells were from GSE99040 (15). The normalized values from these datasets were analyzed for gene expression scores. YAP activated signature was according to the list of activated genes provided in (16), and PD901 activated genes and JQ1 activated/repressed genes (FC of 1.5 and FDR values < 0.05) in MPNST cells were obtained from (17).

Supervised network analysis

– Assignment of active promoters and enhancers bound by BRD proteins

Hg19 TSS coordinates were obtained from (ftp://ccg.vitalit.ch/epdnew/H_sapiens/005/db/promoter_ucsc.txt) and active promoter regions were defined as H3K4me3 peaks lying within the +/-5kb range of TSSs. If one peak falls within more than one promoter region, the one whose TSS coordinate was the closest to the peak midpoint was assigned to that peak. Human enhancer regions and their target genes were obtained from the GeneHancer database (18). H3K4me1 and H3K27ac peaks were defined as overlapping if the midpoint of either peak was falling within the peak range of the other and only the overlapping region was considered as the range. If H3K4me1 + H3K27ac overlapping peaks resided within an enhancer region, this enhancer was considered active. If more than one enhancer contained a H3K4me1 + H3K27ac overlapping peak, the one whose midpoint was the closest to the midpoint of the overlapping peak was assigned to that peak. Binding peaks

of BRD2, BRD3 and BRD4 were assigned to active promoters and enhancers if the peak midpoint fell within their regions. If a peak fell within more than one active promoter/enhancer region, the one whose midpoint was the closest to the midpoint of the peak was considered the target of the peak.

Gene Regulatory Network (GRN) inference and analysis

GRNs for the shControl and shHuR phenotypes were inferred from RNA-seq data, BRD-bound active promoters and enhancers, and literature knowledge. First, differentially expressed genes between the two phenotypes were identified with the p-value cutoff ≤ 0.001 and the absolute fold change ≥ 1.5. In addition, genes were not considered differentially up-regulated if logCPM was below 10 in at least one of the three RNA-seq replicates. Since not only BRD proteins but also TFs targeted by BRD proteins could contribute to the gene expression changes between shControl and shHuR samples, we retrieved from MetaCore (Clarivate Analytics) (19) experimentally validated transcriptional regulatory interactions among differentially expressed, direct and indirect BRD target TFs whose promoters and/or enhancers are active (download date: May 2018). Then, these retrieved interactions were merged with BRD-target binding interactions to form the prior knowledge network (PKN) and this PKN was "contextualized" to each of the two phenotypes using an algorithm developed in (20). Briefly, this algorithm assumes that each cellular phenotype is a Boolean stable steady state attractor of a given network, and removes interactions that are inconsistent with the Booleanized gene expression states. This gene expression Booleanization was performed by treating differentially up-regulated genes as "1" and down-regulated genes as "0". The GRN was clustered based on Gene Ontology categories, and was visualized in Cytoscape version 2.7.0 (21).

In vivo HuR overexpression studies

All inoculations were carried out in female Hsd:Athymic Nude-*Foxn1*$^{nu/nu}$ mice of 8–12 weeks of age. For HuR overexpression studies, immortalized normal human Schwann cell line (iHSCλ2) or immortalized plexiform neurofibroma-derived Schwann cells (ipNFSC) [hTERT NF1 ipNF95.11b (ATCC® CRL3390™)] were infected with control (TRIPZ-HA) or HA-tagged (TRIPZ-HuR) (22) lentivirus and selected with puromycin for 2 days. For xenograft experiments, 1 X 10^6 cells mixed in 1:4 PBS:Matrigel were injected subcutaneously in the right back flank of mice under standard procedures. Mice were fed with doxycycline diet (Open Standard Diet with 2,000 ppm Dox, Research Diets Inc), and then sacrificed 5 weeks later. For experimental lung metastasis experiment, 1 X 10^6 cells were resuspended in PBS and injected in the lateral tail vein of mice. Mice were fed with doxycycline diet, and then euthanized 5 weeks later and lungs were processed for histology upon perfusion with 10% formalin through the trachea.

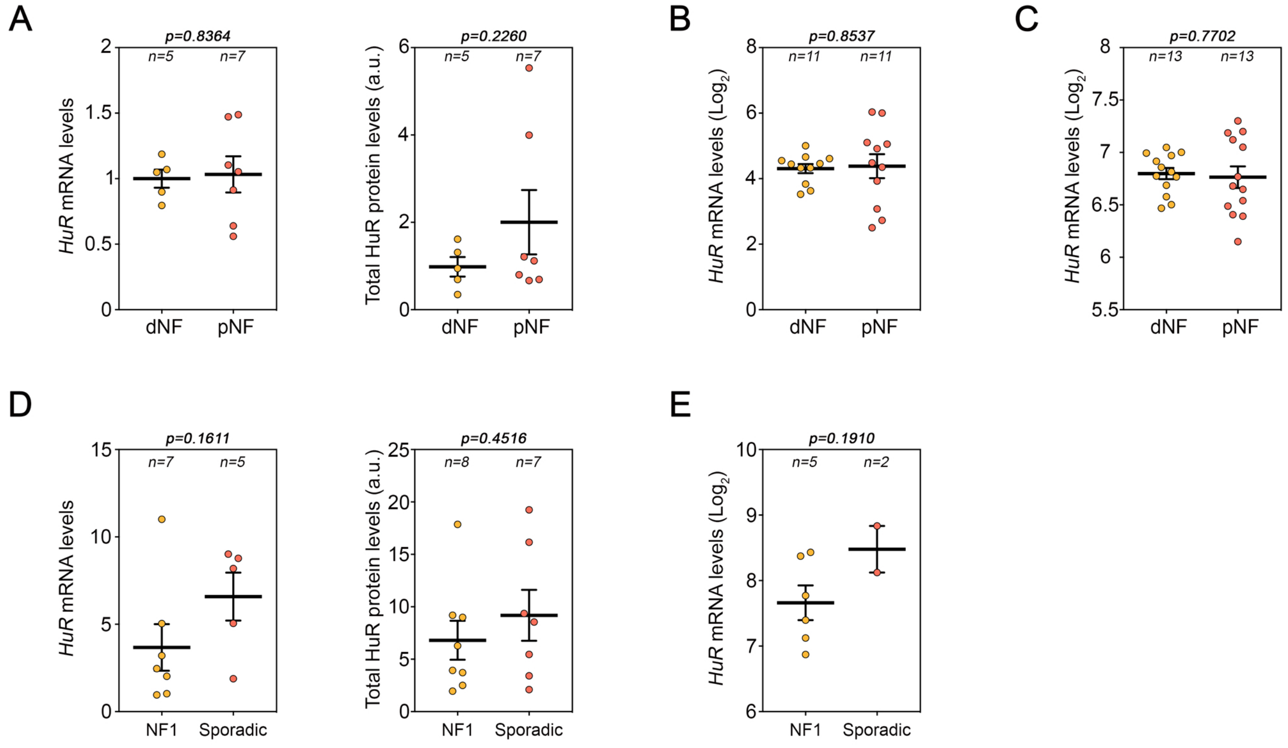

Supplemental Figure 1: HuR is upregulated in human MPNSTs

(A–C) HuR abundance in dermal neurofibromas (dNF) and plexiform neurofibromas (pNF) was assessed by measuring **(A)** the levels of *HuR* mRNA and HuR protein in a human frozen cancer panel (Figure 1D–G), and **(B, C)** the average *HuR* mRNA levels in the **(B)** Miller cohort (GSE 14038) (8) and **(C)** Jessen cohort (GSE 41747) (7).

(D, E) Assessment of HuR expression levels in NF1-MPNST (NF1) tumours and sporadic MPNST by measuring **(D)** the levels of *HuR* mRNA and HuR protein from a human frozen cancer panel (Figure 1D–G), and **(E)** the average mRNA levels in MPNST cell lines in Miller cohort (GSE 14038)(8).

Data are presented as mean ± SEM, two-tailed unpaired Student's t-test; Individual P-values and number of samples (n) per group are shown.

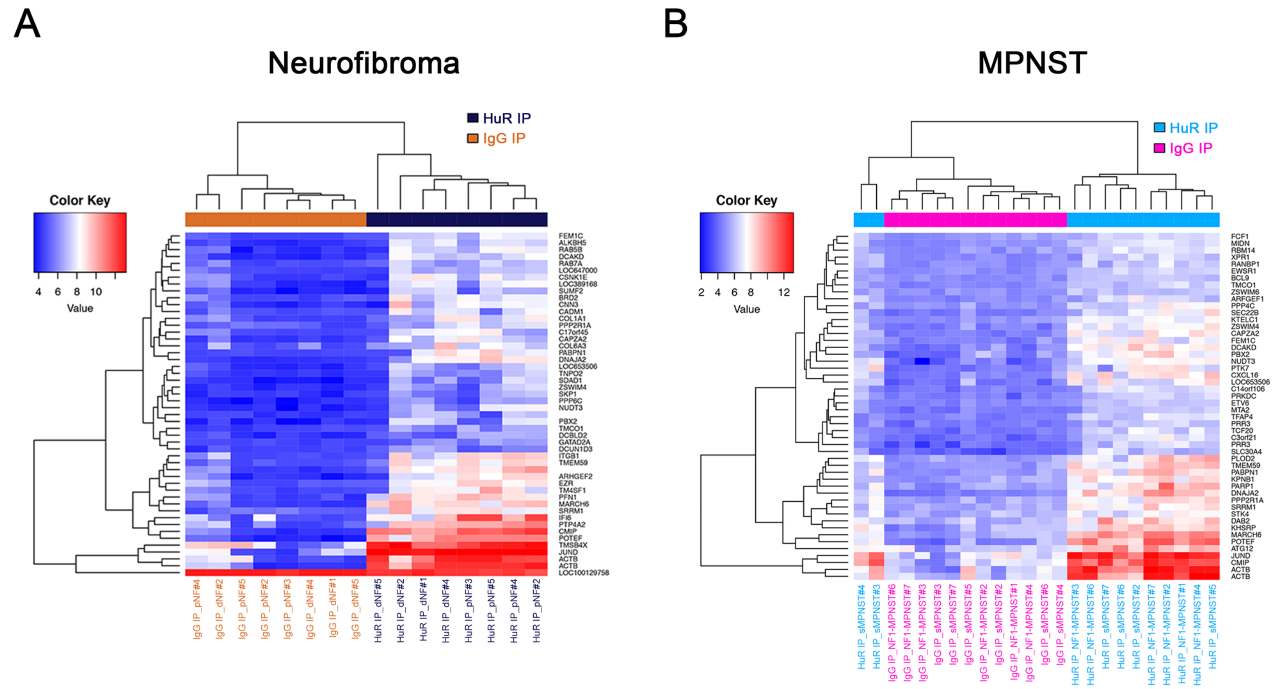

Supplemental Figure 2: HuR is bound to key targets in human MPNSTs

(A, B) Heatmap showing expression of the top transcripts most significantly bound to HuR (HuR IP) compared with control IgG (IgG IP) in **(A)** Neurofibroma samples (n=8) and **(B)** MPNST samples (n=12), obtained from Stanmore Musculoskeletal Biobank. The colour scales indicate the degree of enrichment (red-blue ratio scale). dNF = dermal neurofibroma; pNF = plexiform neurofibroma; sMPNST = sporadic MPNST; NF1-MPNST = NF1-derived MPNST.

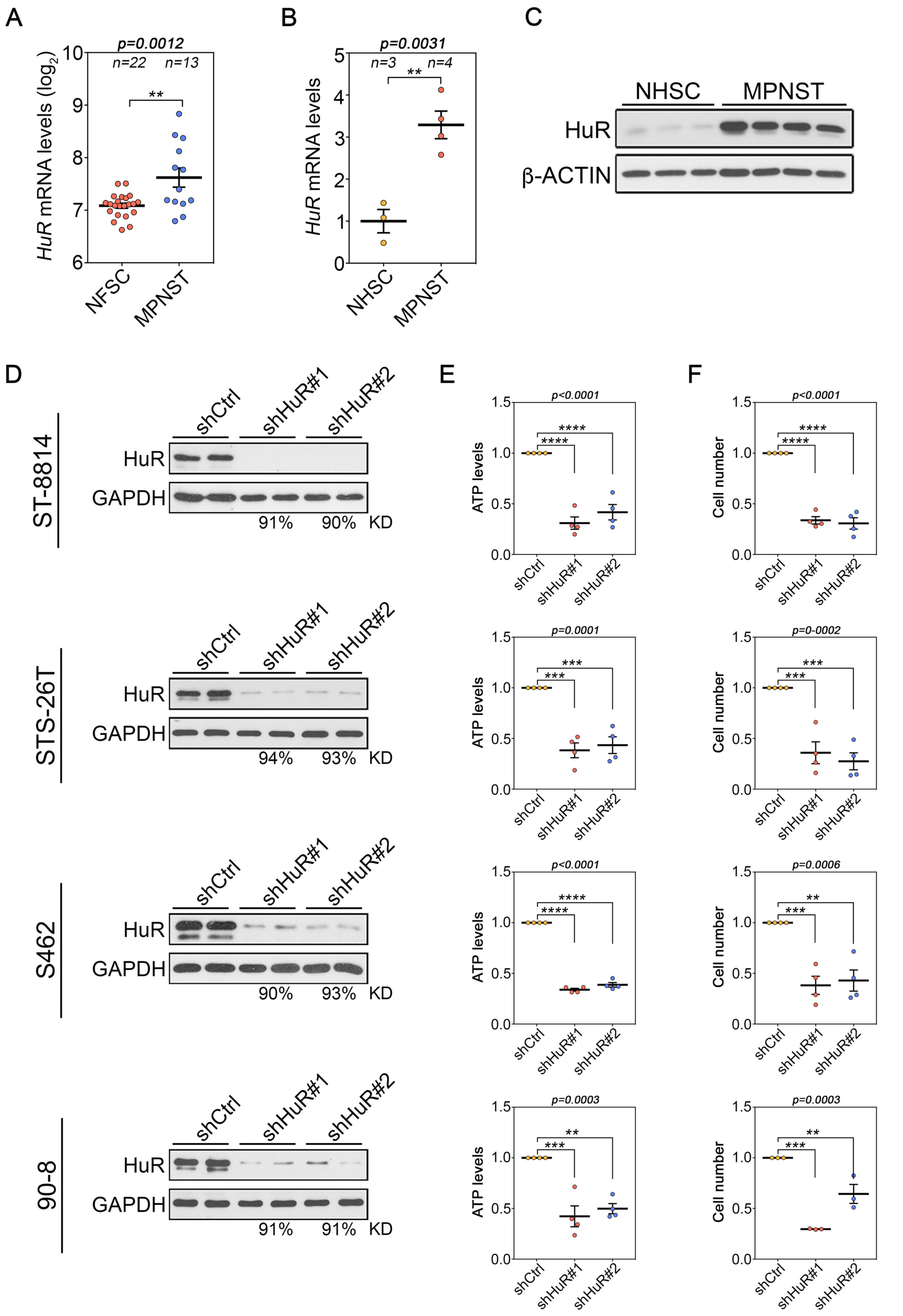

A
p=0.0012
n=22 n=13
**
HuR mRNA levels (log2)
NFSC
MPNST
B
p=0.0031
n=3 n=4
**
HuR mRNA levels
NHSC
MPNST
C
NHSC
MPNST
HuR
β-ACTIN
D
shCtrl
shHuR#1
shHuR#2
ST-8814
HuR
GAPDH
91% 90% KD
STS-26T
HuR
GAPDH
94% 93% KD
S462
HuR
GAPDH
90% 93% KD
90-8
HuR
GAPDH
91% 91% KD
E
p<0.0001
p=0.0001
p<0.0001
p=0.0003
ATP levels
F
p<0.0001
p=0-0002
p=0.0006
p=0.0003
Cell number

Supplemental Figure 3: HuR promotes MPNST cell growth in vitro
(A) *HuR* mRNA expression levels in Neurofibroma-derived Schwann cells (NFSC) and MPNST cell lines from Miller cohort (GSE14038)(8).
Data are presented as mean ± SEM, two-tailed unpaired Student's t-test. The number of samples (n) per group is indicated.
(B) RT-qPCR analysis of *HuR* mRNA levels and **(C)** Western blot analysis of HuR levels, in normal human Schwann cells (NHSC) (n=3), obtained from NHSBT study no. 61 and MPNST cell lines (n=4).
Data are presented as mean ± SEM, two-tailed unpaired Student's t-test. The number of samples (n) per group is indicated.
(D) Growth of MPNST cell lines ST88-14, STS-26T, S462 and 90-8 are sensitive to constitutive HuR silencing in vitro. **(D)** Representative immunoblots of HuR expression after shRNA-mediated knockdown with two distinct HuR-specific sh RNAs (sh HuR#1 and sh HuR#2). β-ACTIN expression was used as a loading control. The percentage of HuR knockdown (KD) was quantified by densitometry. Technical duplicates are shown, and similar results were obtained in at least 3 independent experiments.
(E, F) HuR silencing leads to a reduction in cell growth in MPNST cell lines, as determined by **(E)** ATP luminescence assays and **(F)** counts of cell numbers, 5 days after puromycin selection.
*Data are normalized to shCtrl cells and are presented as mean ± SEM; Each data point represents 1 independent experiment; one-way ANOVA with Tukey's multiple-comparisons test.*p<0.05; **p<0.01, ***p<0.001; ****p<0.0001.*

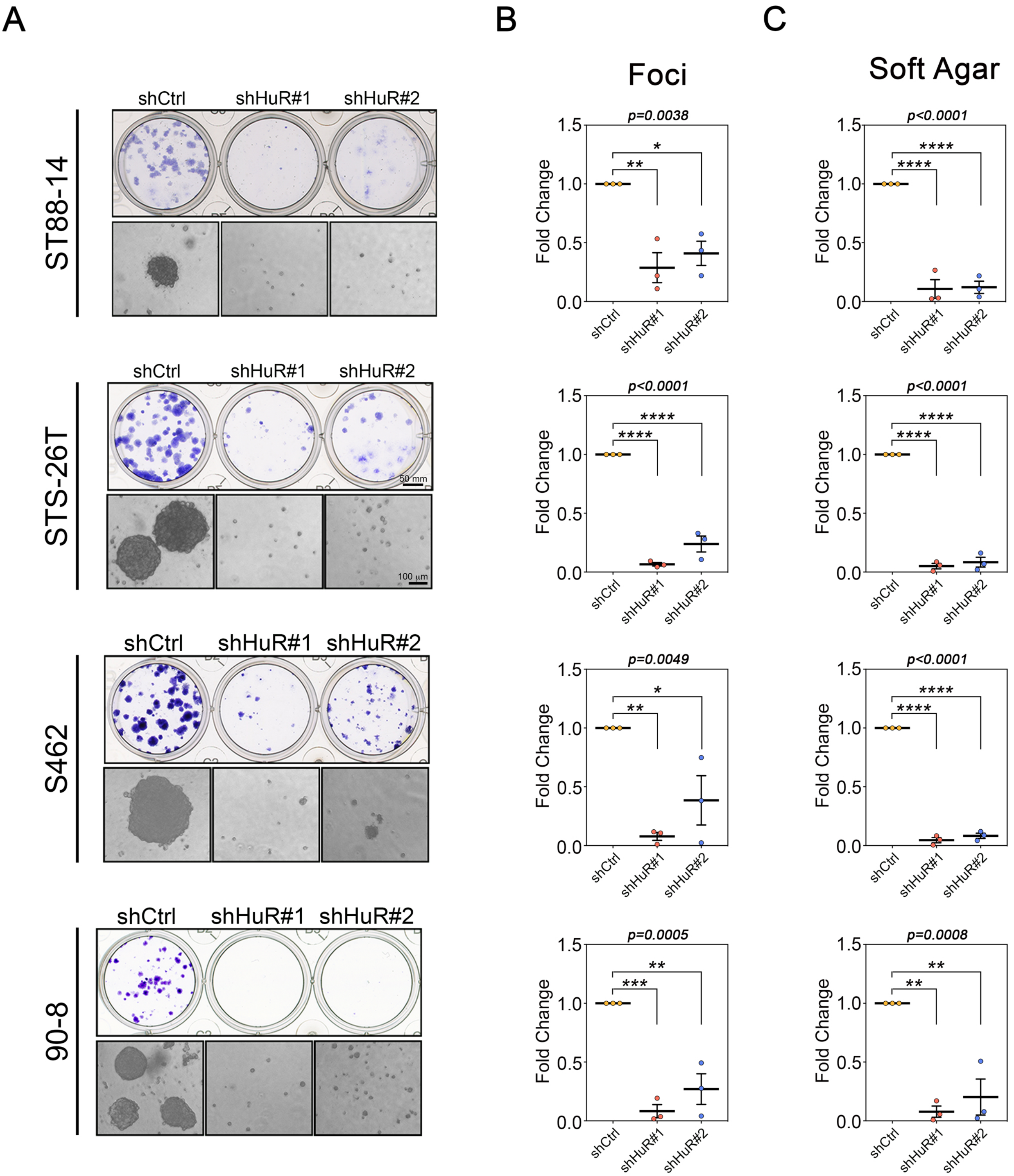

Supplemental Figure 4: HuR promotes MPNST cell growth in vitro
(A–C) HuR silencing leads to a reduction in cell growth in 4 MPNST cell lines, as determined by clonogenic assays, and anchorage-independent growth using soft agar assays. **(A)** Representative pictures of crystal-violet stained colonies in clonogenic assays (top panels), and colonies in soft agar assays (bottom panels) are shown for each cell line. Graphs represent **(B)** absorbance of crystal-violet stained colonies for clonogenic assays, and **(C)** number of colonies in soft agar assays.
Data are normalized to shCtrl cells and are presented as mean ± SEM; Each data point represents 1 independent experiment; one-way ANOVA with Tukey's multiple-comparisons test.$p<0.05$; **$p<0.01$, ***$p<0.001$; ****$p<0.0001$.*

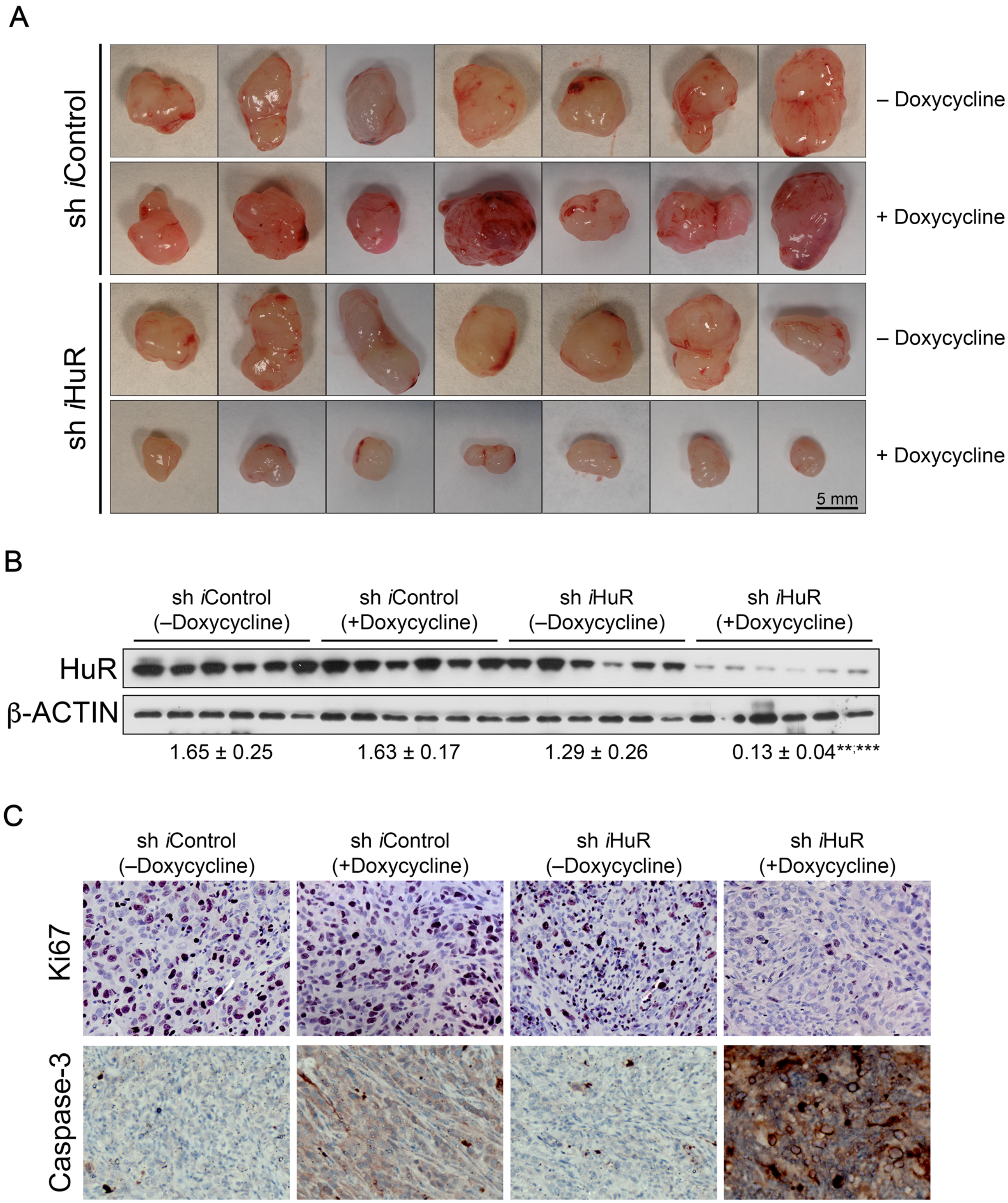

Supplemental Figure 5: HuR silencing in vivo blocks proliferation and induces apoptosis in MPNST tumours

(A) Pictures of all tumours extracted from nude mice for 4 groups of mice: sh *i*Control (–Doxycycline) (n=7), sh *i*Control (+Doxycycline) (n=7), sh *i*HuR (–Doxycycline) (n=7) and sh *i*HuR (+Doxycycline) (n=7), as per experiment described in Figure 3C.

(B) Representative Western blot of total HuR levels from tumours in (A). Densitometry analysis of HuR levels corrected for β-ACTIN was performed for panel of tumours.

*Statistical significance was calculated by one-way ANOVA with Tukey's multiple-comparisons test. **p<0.01 [sh iHuR (*+Doxycycline*) v sh iHuR (*–Doxycycline*)]; ***p<0.001 [sh iHuR (*+Doxycycline *v sh i*Control (+Doxycycline)*].*

(C) Representative immunohistochemistry images of ki67-positive proliferative cells (violet) and apoptotic active Caspase-3 positive (brown) from tumours from Supplemental Figure 5A.

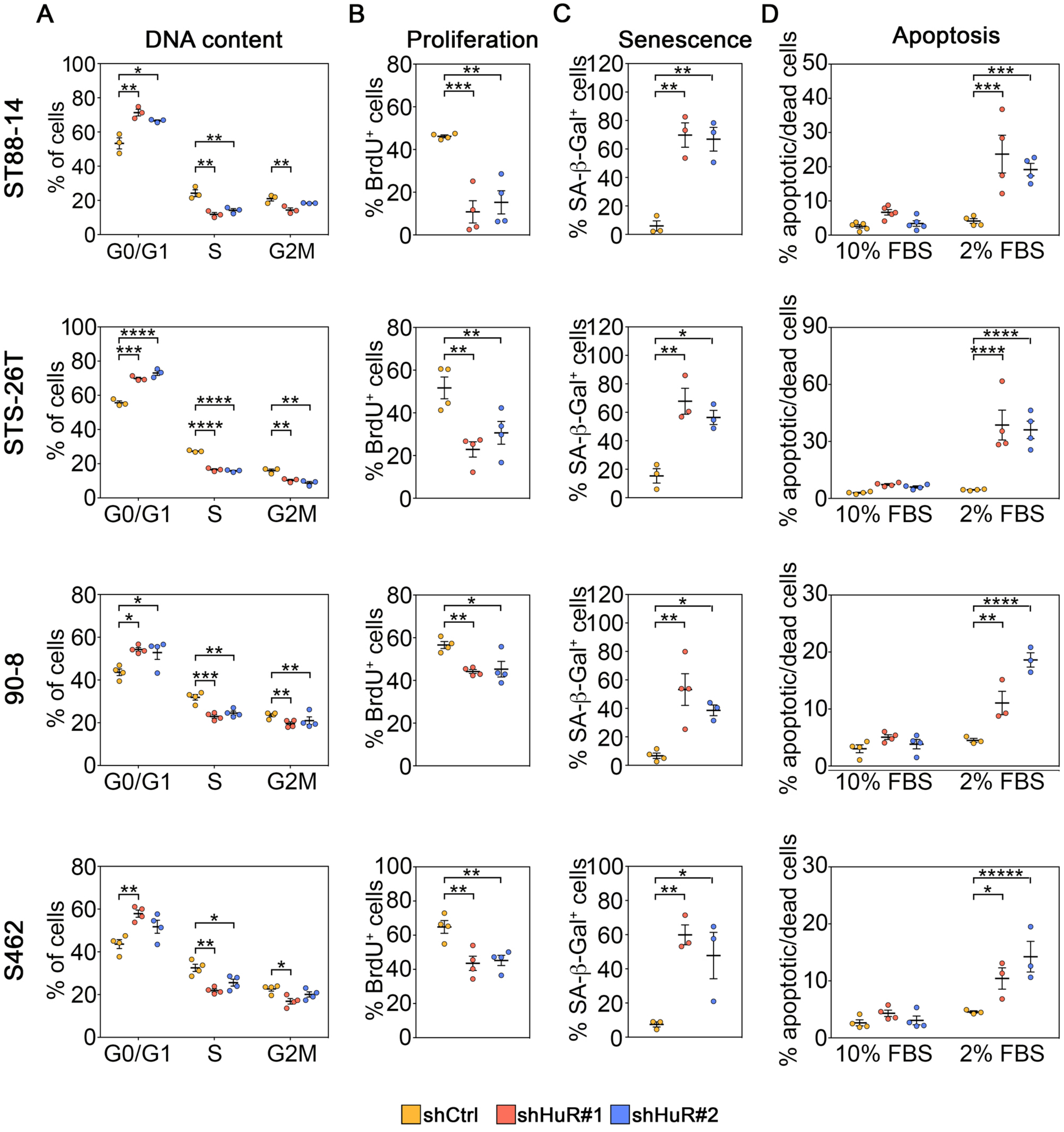

Supplemental Figure 6: HuR depletion induces cell cycle arrest, apoptosis and senescence in MPNST cells

(A) Cell cycle analysis of Propidium Iodide-stained nuclei of MPNST cells after constitutive HuR silencing in vitro with two distinct HuR-specific sh RNAs (sh HuR#1 and sh HuR#2) in 4 cell lines.

Data are presented as mean ± SEM; Each data point represents 1 independent experiment; one-way ANOVA with Tukey's multiple-comparisons test.

(B) Percentage of BrdU positive proliferative cells after HuR silencing.

Data are presented as mean ± SEM; Each data point represents 1 independent experiment; one-way ANOVA with Tukey's multiple-comparisons test.

(C) Percentage of SA-β-Gal-positive cells after HuR silencing.

Data are presented as mean ± SEM; Each data point represents 1 independent experiment; one-way ANOVA with Tukey's multiple-comparisons test.

(D) Apoptosis induction as measured by flow cytometry analysis for Annexin V (+) cells after HuR silencing in growth-promoting (10% FBS) and growth-limiting conditions (2% FBS).

*Data are presented as mean ± SEM; Each data point represents 1 independent experiment; two-way ANOVA with Tukey's multiple-comparisons test. *p<0.05; **p<0.01, ***p<0.001; ****p<0.0001.*

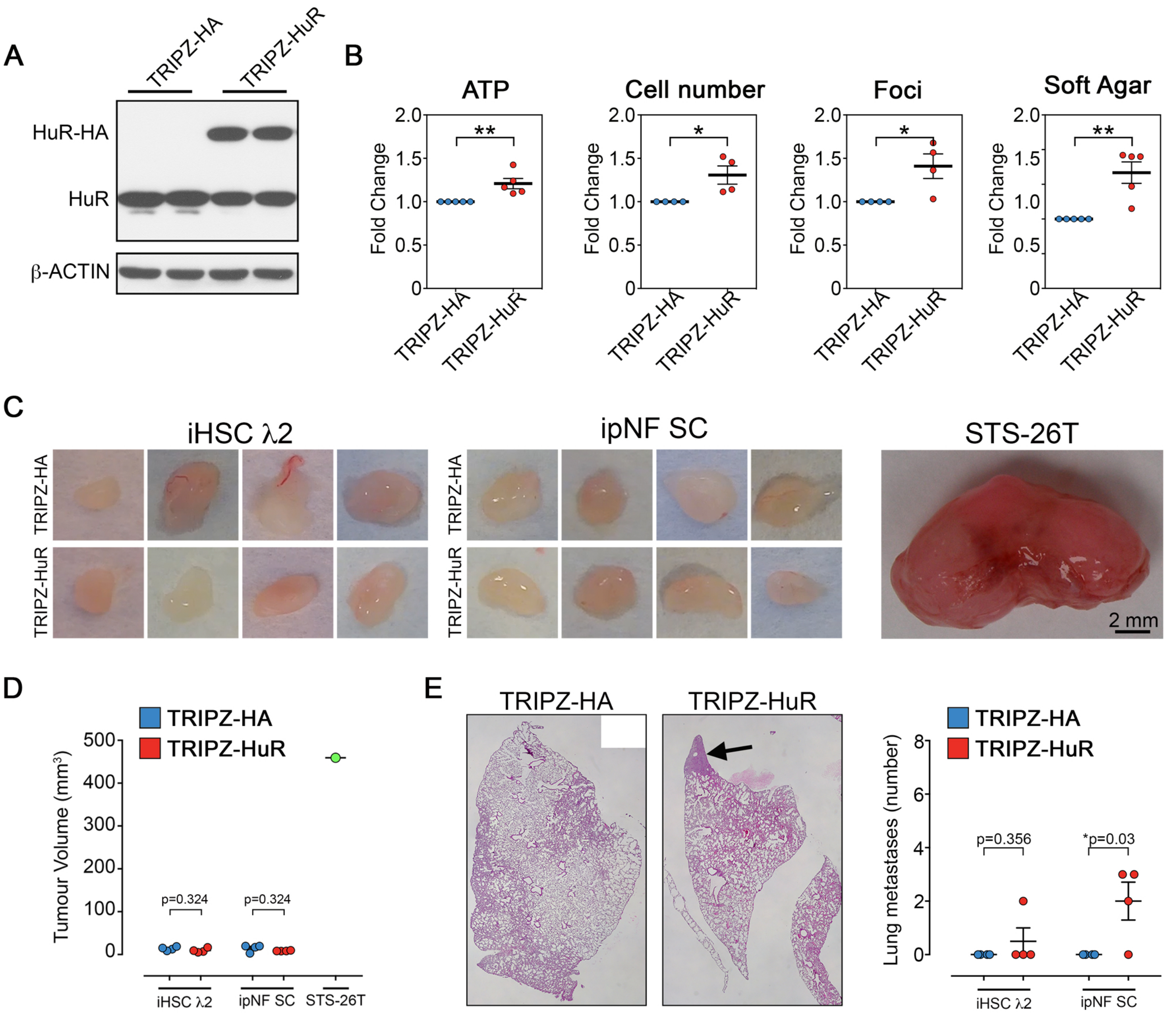

Supplemental Figure 7: HuR overexpression does not lead to tumour formation and overt metastasis in normal or plexiform neurofibroma Schwann cells.

(A) Representative immunoblots showing increased expression of HuR tagged with HA after infection with a lentiviral vector (TRIPZ-HuR) in immortalized normal human Schwann cell line. β-ACTIN expression was used as a loading control. Technical duplicates are shown, and similar results were obtained in at least 3 independent experiments.

(B) HuR overexpression leads to a slight increase in cell growth in immortalized normal human Schwann cell line, as determined by ATP luminescence assays, counts of cell numbers, clonogenic assays (foci), and anchorage-independent growth using soft agar assays.

Data are normalized to control TRIPZ-HA infected cells and are presented as mean ± SEM; Each data point represents 1 independent experiment; two-tailed unpaired Student's t tests.

(C–D) HuR overexpression does not induce an increase in tumour size in immortalized normal human Schwann cell line (iHSCλ2) or immortalized plexiform neurofibroma Schwann cell line (ipNF SC). In comparison, tumour formed by MPNST cell line STS-26T is comparatively much larger. (**C**) Pictures of tumours extracted from nude mice **(D)** Graph showing volume of tumours at extraction.

(E) HuR overexpression dos not lead to overt formation of metastatic nodules in immortalized normal human Schwann cell line (iHSCλ2) or immortalized plexiform neurofibroma Schwann cell line (ipNF SC). Pictures shows formation of a small metastatic nodule in HuR-overexpressing ipNF SC (arrow). A small but significant increase in formation of metastatic nodules was observed only in the case of HuR-overexpressing ipNF SC, but not in HuR-overexpressing iHSCλ2 cell lines.

*Data are normalized to control TRIPZ-HA infected cells and are presented as mean ± SEM; n=4 mice; two-tailed unpaired Student's t tests. *p<0.05; **p<0.01.*

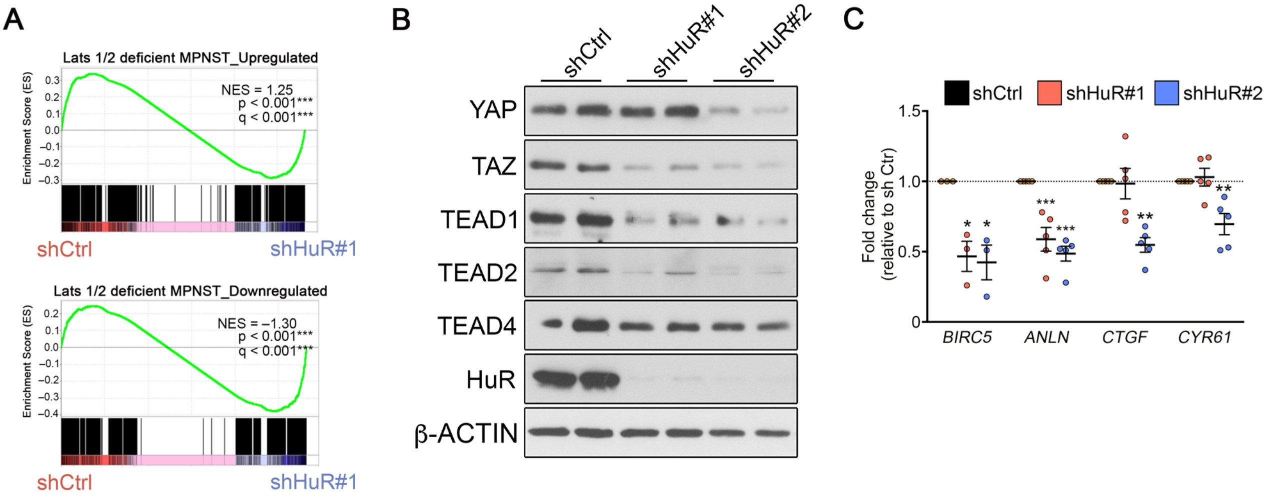

Supplemental Figure 8: HuR regulates the YAP/TAZ pathway in MPNST line STS-26T

(A) GSEA plots showing enrichment of genes upregulated and downregulated by YAP/TAZ activation in mouse Schwann cells (after ablation of their negative regulators LATS1/2), and shCtrl and sh HuR#1 ST88-14 MPNST cells respectively.

(B) Representative Western blot showing a general downregulation of key YAP/TAZ pathway components after HuR silencing in STS-26T MPNST cells. Technical duplicates are shown, and similar results were obtained in at least 3 independent experiments.

(C) RT-qPCR analysis showing downregulation of YAP/TAZ pathway effector genes after HuR silencing in STS-26T MPNST cells (n= 3–5 independent experiments).

*Data are normalized to shCtrl cells and are presented as mean ± SEM; n=3–5 independent experiments; one-way ANOVA with Tukey's multiple-comparisons test. *p<0.05; **p<0.01, ***p<0.001.*

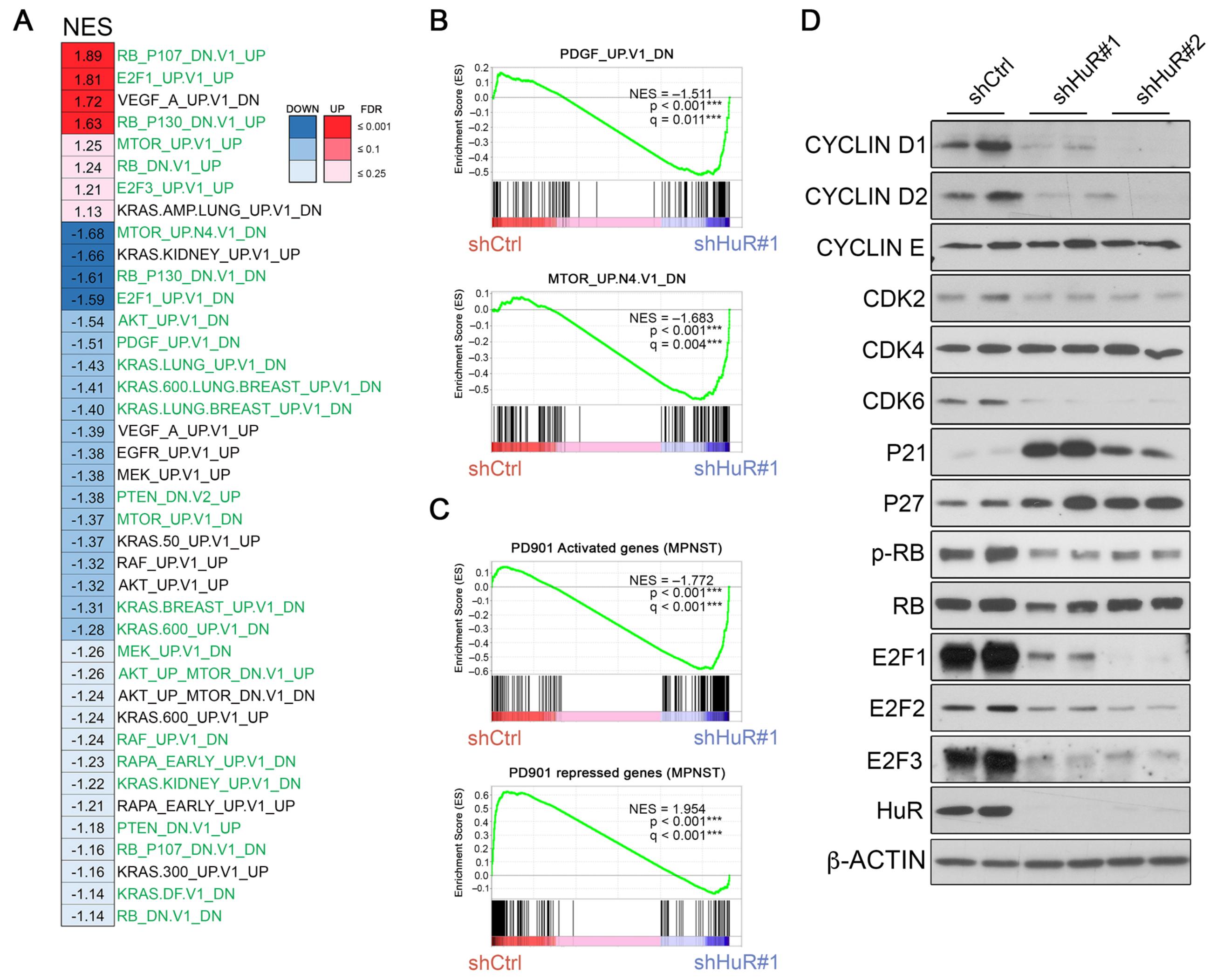

Supplemental Figure 9: HuR regulates the RB-E2F pathway in MPNST line STS-26T

(A) Compendium of normalized enrichment scores (NES) of all target gene sets associated with RAS-MEK-ERK, PI3K-AKT-mTOR and RB-E2F pathways after GSEA analysis of HuR–silenced ST88-14 MPNST cells after RNA-sequencing (Supplemental Table 4). Notably, there is general positive correlation in the activation of the pathways (highlighted in green) in sh Control-infected compared to sh HuR#1-infected ST88-14 MPNST cells. The colour codes represent FDR q values (red-blue ratio scale).

(B) GSEA plots showing enrichment of genes downregulated by PDGF receptor activation and mTOR pathway activation in sh HuR#1-infected compared to sh Control-infected ST88-14 MPNST cells from Supplemental Figure 9A.

(C) GSEA plots showing enrichment of genes activated by PD901 treatment of MPNST cells (FC >2 and adjusted p-value<0.05)(13) in sh HuR#1-infected compared to sh Control-infected ST88-14 MPNST cells, and conversely, enrichment of genes repressed by PD901 treatment in sh Control-infected compared to sh HuR#1-infected ST88-14 MPNST cells.

(D) Representative Western blot showing a downregulation of several key RB-E2F pathway components after HuR silencing in STS-26T MPNST cells. Technical duplicates are shown, and similar results were obtained in 3 independent experiments.

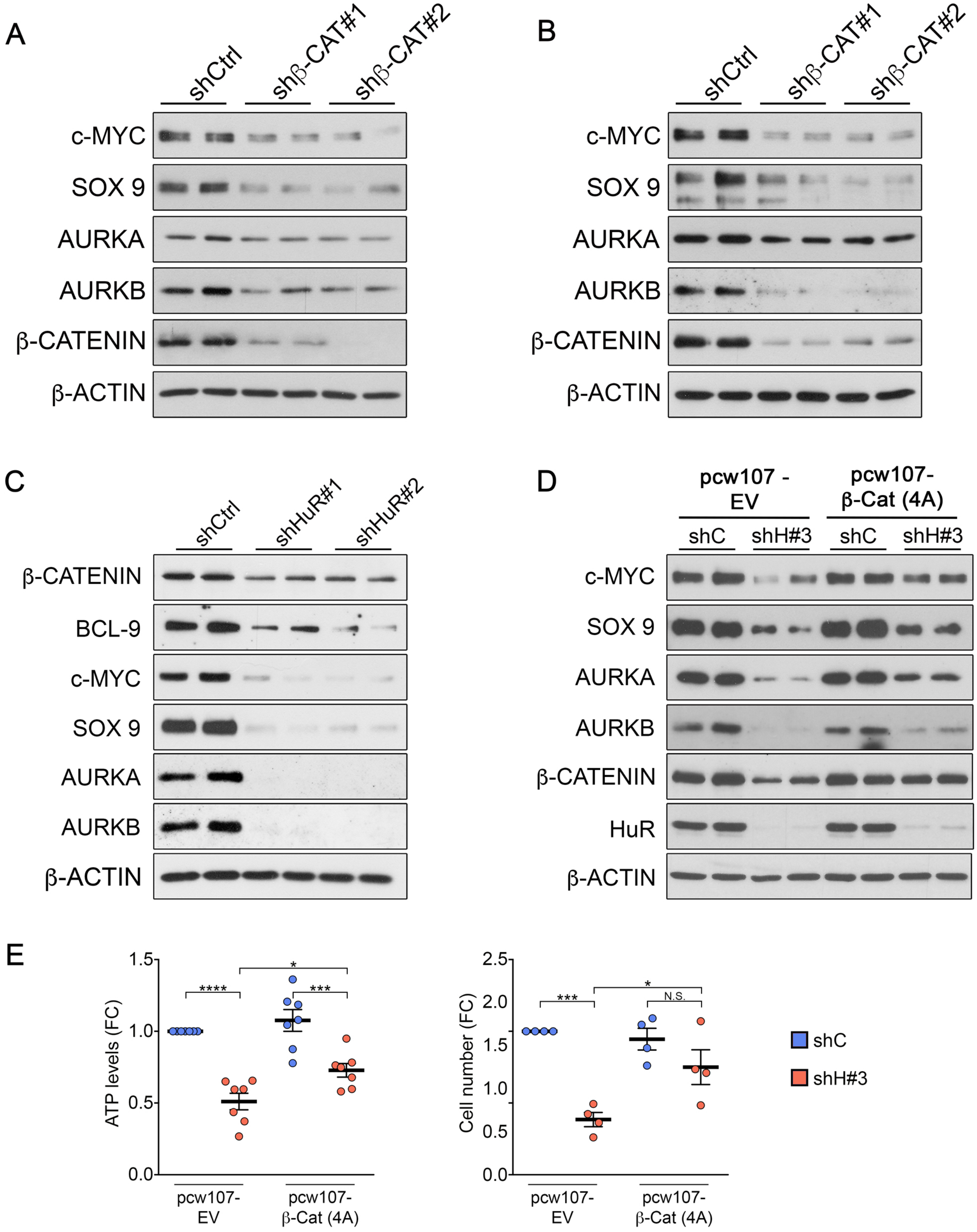

Supplemental Figure 10: HuR activates key Wnt/β-Catenin-mediated oncogenic programs in ST88-14 and STS-26T cells

(A, B) Representative Western blots showing a general downregulation of Wnt/β-catenin pathway components, including key oncogenic downstream regulators, after β-Catenin silencing in **(A)** ST88-14 and **(B)** STS-26T MPNST cells. Technical duplicates are shown, and similar results were obtained in 3 independent experiments.

(C) Representative Western blot showing a general downregulation of Wnt/β-catenin pathway components, including key oncogenic downstream regulation, after HuR silencing in STS-26T MPNST cells. Technical duplicates are shown, and similar results were obtained in 3 independent experiments.

(D) Representative Western blot showing that lentivirus-based expression of constitutively active β-catenin 4A mutant (harbours alanine substitutions at S33, S37, T41, and S45, preventing its degradation)[pcw107–β-Cat (A)], partially blocks the downregulation of the key downstream regulators c-MYC, SOX9, AURKA and AURKB by HuR silencing (shH#3) in STS-26T MPNST cells. Technical duplicates are shown, and similar results were obtained in 3 independent experiments.

(E) Ectopic expression of constitutively active β-catenin 4A mutant partially blocks the effects of HuR silencing on cell numbers and ATP levels in STS-26T MPNST cells.

*Data are normalized to shC + pcw107-EV cells and are presented as mean ± SEM; Each data point represents 1independent experiment; one-way ANOVA with Tukey's multiple-comparisons test. *p<0.05; **p<0.01, ***p<0.001, ****p<0.001.*

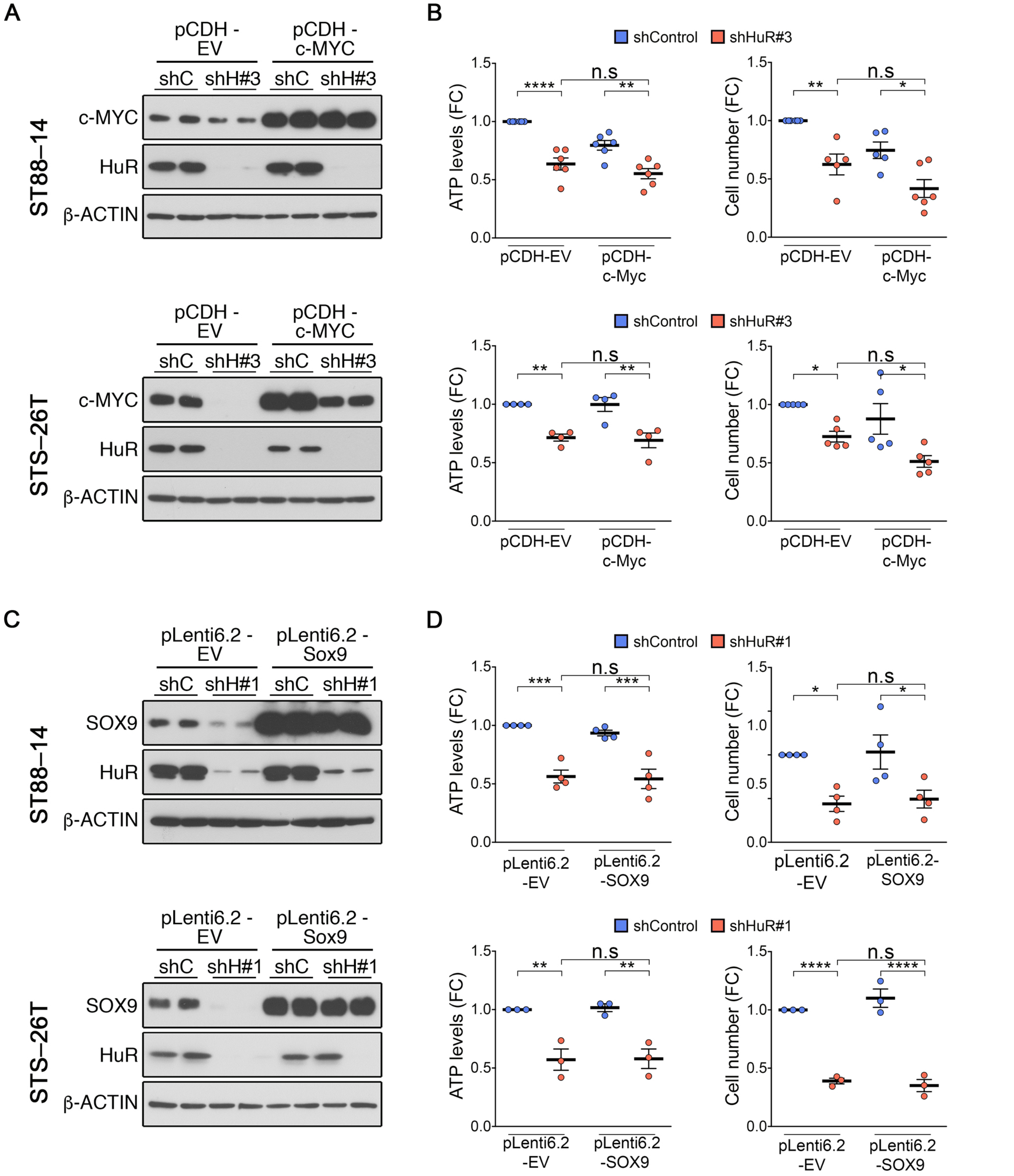

Supplemental Figure 11: HuR silencing-mediated effects on cell growth cannot be rescued by c-MYC or SOX9 overexpression.

(A) Representative Western blot showing that expression of c-MYC and HuR after lentivirus-based expression of c-MYC (p-CDH-c-MYC) or empty vector (pCDH-EV) and HuR silencing (shH#3) in ST88-14 and STS-26T MPNST cells. Technical duplicates are shown, and similar results were obtained in 3 independent experiments.

(B) Ectopic expression of c-MYC cannot rescue effects of HuR silencing on cell numbers and ATP levels in ST88-14 and STS-26T MPNST cells.

Data are normalized to shControl + pCDH-EV cells and are presented as mean ± SEM; Each data point represents 1 independent experiment; one-way ANOVA with Tukey's multiple-comparisons test.

(C) Representative Western blot showing that expression of SOX9 and HuR after lentivirus-based expression of SOX9 (pLenti6.2-SOX9) or empty vector (pLenti6.2-EV) and HuR silencing (shH#1) in ST88-14 and STS-26T MPNST cells. Technical duplicates are shown, and similar results were obtained in 3 independent experiments.

(B) Ectopic expression of Sox9 cannot rescue effects of HuR silencing on cell numbers and ATP levels in ST88-14 and STS-26T MPNST cells.

*Data are normalized to shControl + pLenti6.2-EV cells and are presented as mean ± SEM; Each data point represents 1 independent experiment; one-way ANOVA with Tukey's multiple-comparisons test. *p<0.05; **p<0.01, ***p<0.001, ****p<0.001*

A

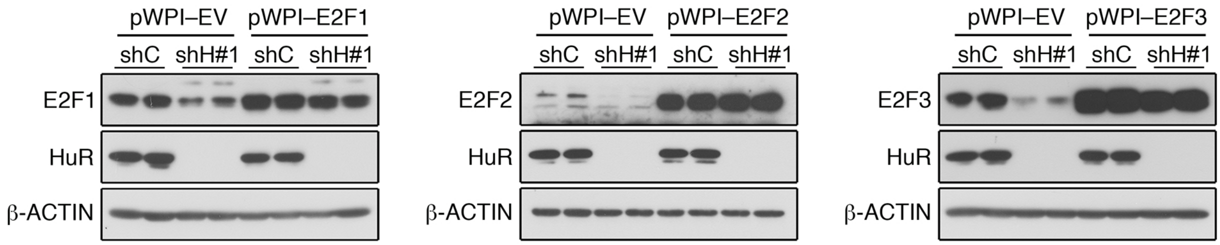

B

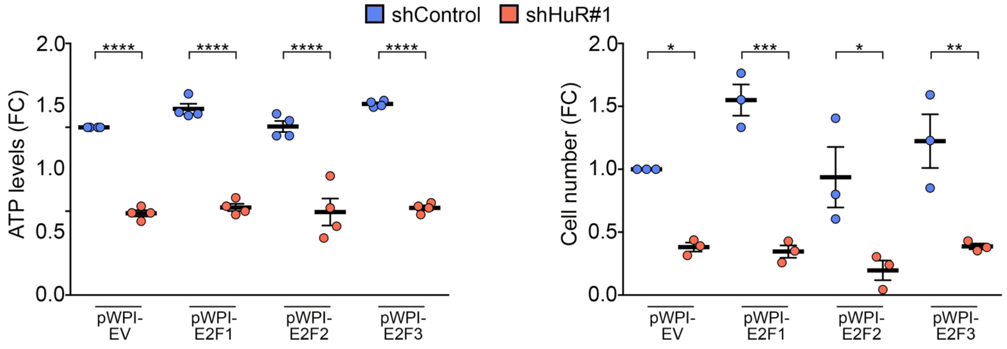

C

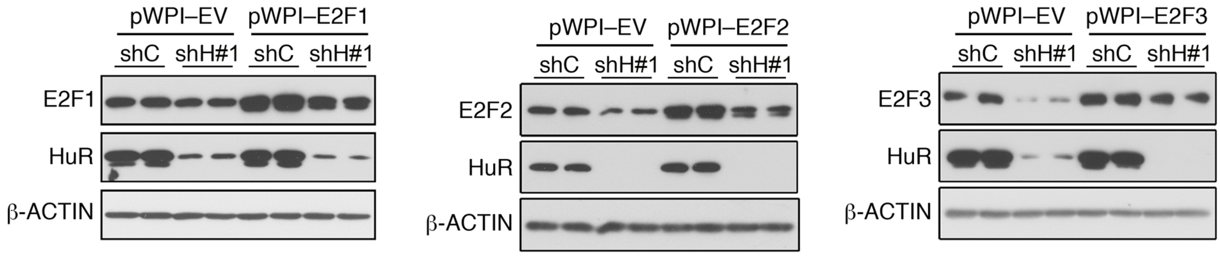

D

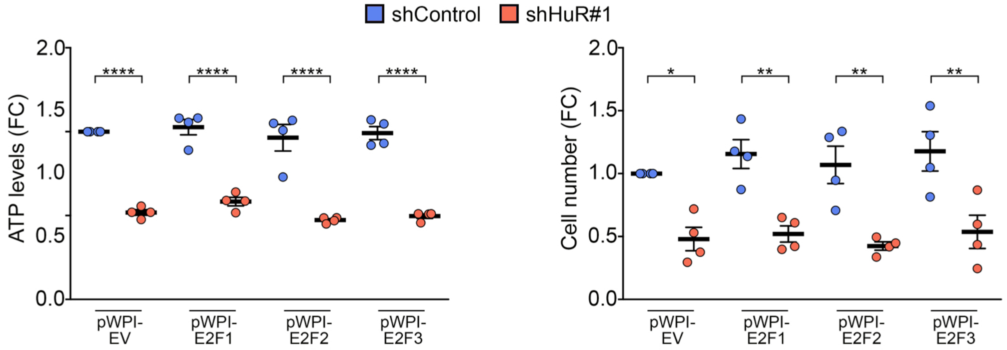

Supplemental Figure 12: HuR silencing-mediated effects on cell growth cannot be rescued by E2Fs overexpression.

(A) Representative Western blot showing expression of E2Fs and HuR after lentivirus-based expression of E2Fs (pWPI-E2Fs) or empty vector (pWPI-EV), and HuR silencing (shHuR#1) in ST88-14 MPNST cells. Technical duplicates are shown, and similar results were obtained in 3 independent experiments.

(B) Ectopic expression of E2Fs cannot rescue effects of HuR silencing on cell numbers and ATP levels in ST88-14 MPNST cells.

Data are normalized to shControl + pWPI-EV cells and are presented as mean ± SEM; Each data point represents 1 independent experiment; one-way ANOVA with Tukey's multiple-comparisons test.

(C) Representative Western blot showing expression of E2Fs and HuR after lentivirus-based expression of E2Fs (pWPI-E2Fs) or empty vector (pWPI-EV), and HuR silencing (shHuR#1) in STS-26T MPNST cells. Technical duplicates are shown, and similar results were obtained in 3 independent experiments.

(D) Ectopic expression of E2Fs cannot rescue effects of HuR silencing on cell numbers and ATP levels in STS-26T MPNST cells.

Data are normalized to shControl + pWPI-EV cells and are presented as mean ± SEM; Each data point represents 1 independent experiment; one-way ANOVA with Tukey's multiple-comparisons test.

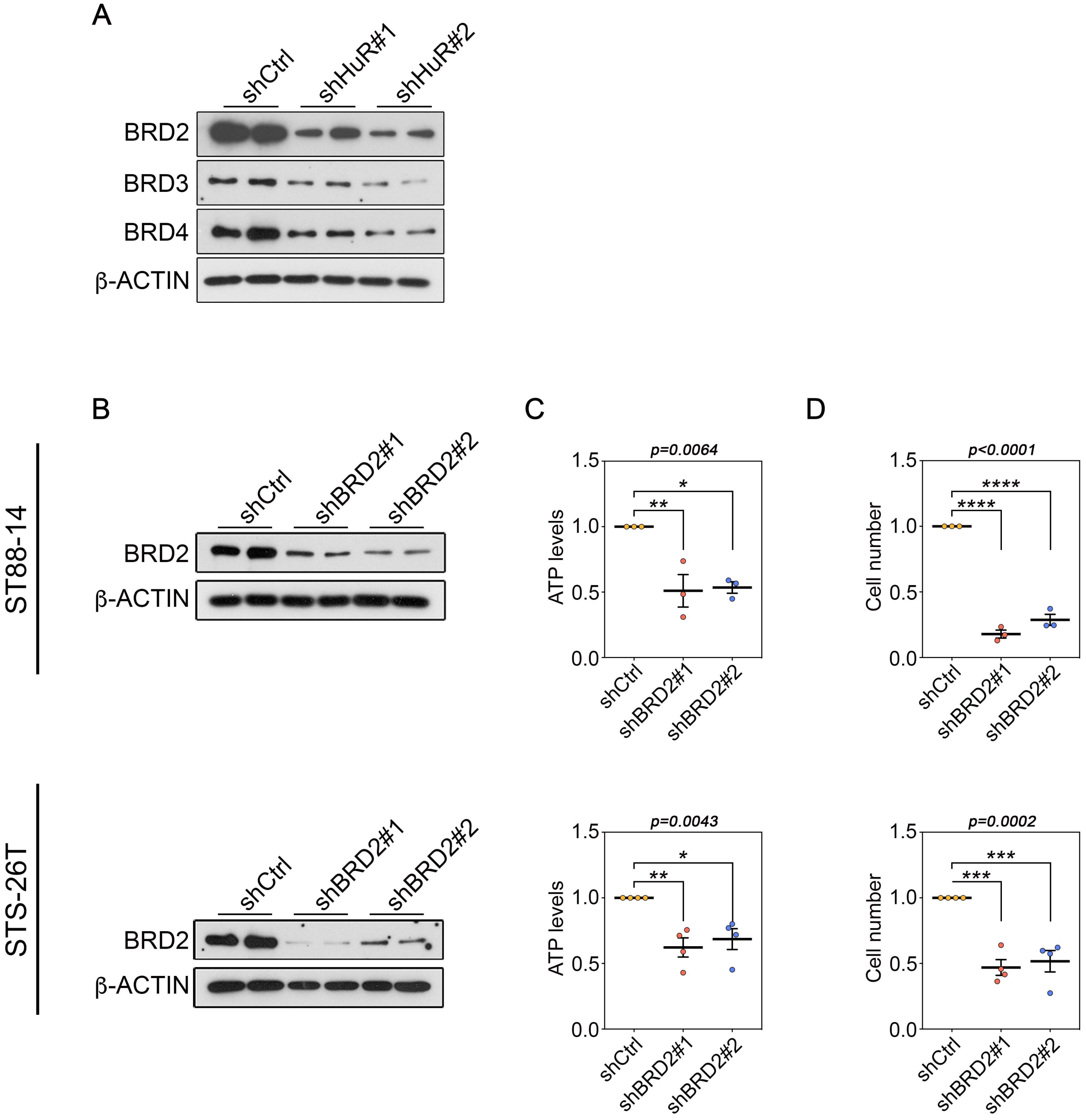

Supplemental Figure 13: HuR regulates expression of BRD proteins in STS-26T cells.
(A) Representative Western blot showing a downregulation of BRD proteins after HuR silencing in STS-26T MPNST cells. Technical duplicates are shown, and similar results were obtained in 3 independent experiments.
(B–D) Growth of MPNST cell lines ST88-14 and STS-26T are sensitive to constitutive BRD2 silencing in vitro.
(B) Representative immunoblots of BRD2 expression after shRNA-mediated knockdown with two distinct BRD2-specific sh RNAs (sh BRD2#1 and sh BRD2#2). β-ACTIN expression was used as a loading control. Technical duplicates are shown, and similar results were obtained in at least 3 independent experiments.
(C, D) BRD2 silencing leads to a reduction in cell growth in both cell lines, as determined by **(C)** ATP luminescence assays and **(D)** counts of cell numbers, 5 days after selection.
*Data are normalized to shCtrl cells and are presented as mean ± SEM; Each data point represents 1 independent experiment; one-way ANOVA with Tukey's multiple-comparisons test. *p<0.05; **p<0.01, ***p<0.001, ****p<0.001.*

Supplementary Table 1:

List of List of HuR targets (fold-change >1.5; adjusted p-value<0.05) identified after RIP-chip in neurofibroma samples

ProbeID	SYMBOL	Acc_No	Fold Change	P-Value	Adjusted P-Value	Mean Signal (HuR IP)	Mean Signal (IgG IP)
ILMN_1869109		AK095855	11.89	1.91E-06	0.000660274	8.68454	5.11286
ILMN_2152131	ACTB	NM_001101.2	81.2304	4.50E-10	8.53E-07	11.73842	5.39448
ILMN_1657283	ALKBH5	NM_017758.3	6.2082	4.89E-07	0.000206134	7.24846	4.6143
ILMN_1703477	ARHGEF2	NM_004723.2	8.6818	1.81E-05	0.00339241	7.93748	4.81948
ILMN_1758918	BRD2	NM_005104.2	4.9547	3.15E-05	0.00488611	7.53158	5.22277
ILMN_1768870	CAPZA2	NM_006136.2	6.2161	3.18E-06	0.000949275	7.41389	4.77788
ILMN_1738075	CMIP	NM_030629.1	61.698	7.70E-13	1.02E-08	10.96292	5.01576
ILMN_1811648	DCAKD	NM_024819.3	7.2111	4.98E-07	0.000206561	7.36123	4.51101
ILMN_1770127	DNAJA2	NM_005880.2	10.8066	2.07E-06	0.000695643	7.98767	4.55383
ILMN_3272378	EZR	NM_003379.4	6.0972	4.71E-05	0.00669565	8.21747	5.60933
ILMN_1703330	FEM1C	NM_020177.2	4.9192	1.33E-06	0.000482913	7.2957	4.99727
ILMN_1687384	IFI6	NM_022873.2	15.7898	1.81E-05	0.00339241	9.82157	5.84065
ILMN_1723467	ITGB1	NM_002211.2	7.4209	5.81E-05	0.00771731	8.2881	5.39652
ILMN_1810214	JUND	NM_005354.3	93.4441	1.40E-10	3.10E-07	13.03501	6.48898
ILMN_3210917	LOC389168	XR_019471.2	5.6767	7.85E-05	0.00946314	7.35711	4.85205
ILMN_1780861	LOC653506	XM_927769.1	4.1868	1.53E-05	0.00294781	6.62863	4.5628
ILMN_1757106	Mar-06	NM_005885.2	18.1587	1.34E-11	7.12E-08	9.13837	4.95578
ILMN_1724907	NUDT3	NM_006703.2	5.6172	4.63E-05	0.00660764	6.78017	4.29033
ILMN_1682699	PBX2	NM_002586.4	5.4453	6.75E-05	0.00862275	6.70092	4.25592
ILMN_2151817	PFN1	NM_005022.2	12.5648	8.90E-09	7.88E-06	8.88084	5.22953
ILMN_3241758	POTEF	NM_001099771.1	48.0562	3.36E-11	1.27E-07	10.12707	4.54042
ILMN_1810467	PPP2R1A	NM_014225.3	3.3428	5.90E-05	0.00779865	7.05536	5.31431
ILMN_1664921	PPP6C	NM_002721.3	3.3444	1.88E-05	0.00348282	5.97299	4.23126
ILMN_2338997	PTP4A2	NM_080392.2	13.7479	1.16E-06	0.000437019	9.52729	5.74615
ILMN_1752582	RAB5B	NM_002868.2	6.366	2.57E-05	0.00437656	7.39325	4.72288
ILMN_1716524	RAB7A	NM_004637.5	3.0189	3.77E-05	0.00552441	6.8136	5.21959
ILMN_1735360	SDAD1	NM_018115.2	4.1722	3.71E-05	0.00550918	6.28254	4.22173
ILMN_1697670	SRRM1	NM_005839.3	7.9294	7.60E-09	7.21E-06	8.51041	5.5232
ILMN_1770338	TM4SF1	NM_014220.2	7.2972	1.48E-05	0.00289129	8.07903	5.21168
ILMN_1793829	TMCO1	NM_019026.2	2.6558	2.89E-05	0.00470884	6.25253	4.8434
ILMN_1792508	TMEM59	NM_004872.3	8.5536	1.51E-07	8.90E-05	8.35455	5.25801
ILMN_1683271	TMSB4X	NM_021109.2	22.91	1.40E-06	0.000501058	12.16864	7.65074
ILMN_1656066	TNPO2	NM_013433.3	3.4409	3.01E-05	0.00480487	6.44281	4.66001
ILMN_2150654	ZSWIM4	NM_023072.1	3.7871	5.71E-05	0.0076602	6.37791	4.45681
ILMN_1730995	AFAP1L2	NM_001001936.1	1.8923	0.000555549	0.0386667	5.31942	4.39931
ILMN_2188204	ATG12	NM_004707.2	4.0786	0.000331547	0.0273434	6.94318	4.9151
ILMN_1719224	C17orf45	NM_152350.2	4.2921	0.000150019	0.014921	7.38229	5.2806
ILMN_1680132	CADM1	NM_014333.3	4.0821	0.000150947	0.0149573	7.24278	5.21348
ILMN_1803429	CD44	NM_001001391.1	4.1078	0.000582276	0.0399558	6.5288	4.49042
ILMN_1693014	CEBPB	NM_005194.2	4.817	0.000476726	0.0349722	7.24914	4.98101
ILMN_1782439	CNN3	NM_001839.2	7.0266	8.87E-05	0.0102888	7.56387	4.75104
ILMN_1701308	COL1A1	NM_000088.3	4.7808	0.000100305	0.0111452	7.48556	5.2283
ILMN_1706643	COL6A3	NM_057165.2	4.1235	0.000231804	0.0208671	7.53606	5.49219
ILMN_2415235	CSNK1E	NM_152221.2	4.9261	0.000226554	0.0204638	7.47532	5.17488
ILMN_1728478	CXCL16	NM_022059.1	5.7475	0.000277603	0.0238577	7.06276	4.53984
ILMN_2174127	DCBLD2	NM_080927.3	2.8138	0.000110035	0.0119856	6.12446	4.63193
ILMN_1665455	DCUN1D3	NM_173475.1	2.2786	0.000135472	0.0136791	6.01645	4.82833
ILMN_1706502	EIF2AK2	NM_002759.1	4.3353	0.000288333	0.0244631	6.89688	4.78075
ILMN_1673682	GATAD2A	NM_017660.2	2.1831	0.000138441	0.0138733	6.04054	4.91416
ILMN_2130411	KDELR1	NM_006801.2	2.4628	0.000484897	0.0351465	5.802	4.50169
ILMN_1673936	KHSRP	NM_003685.2	4.0095	0.0004071	0.0312455	6.94906	4.94563
ILMN_1703949	KPNB1	NM_002265.4	3.0771	0.00026875	0.0233233	6.6344	5.01282
ILMN_1739325	LOC284023	XM_941810.2	1.7397	0.000782212	0.0491074	5.42118	4.62231
ILMN_1704750	LOC647000	XM_929980.2	3.1257	8.78E-05	0.0102644	6.76619	5.12202
ILMN_1735180	NCSTN	NM_015331.2	4.172	0.000285658	0.0243714	6.13039	4.06964
ILMN_1759154	PABPN1	NM_004643.1	6.1835	9.20E-05	0.0104813	7.59528	4.96686
ILMN_2312296	PCBP2	NM_005016.3	5.8208	0.000381486	0.0299817	7.41217	4.87096
ILMN_2342695	PDGFA	NM_033023.3	1.9677	0.000684442	0.0454401	5.83078	4.85425
ILMN_1745329	PRR14	NM_024031.2	2.3519	0.000756519	0.0481105	5.77851	4.54467
ILMN_1659206	RARA	NM_000964.2	2.1821	0.000749922	0.0481037	5.613	4.48731
ILMN_1666739	RBM15	NM_022768.4	1.9553	0.000301504	0.0253378	5.10071	4.13334
ILMN_1808157	RUNDC3B	NM_138290.1	2.0568	0.000499884	0.0356852	5.8283	4.78793
ILMN_1655595	SERPINE2	NM_006216.2	9.9343	0.000283066	0.0242487	8.37039	5.05798
ILMN_1697469	SFRS6	NM_006275.4	2.8006	0.000599738	0.0409426	6.54814	5.06239
ILMN_3229770	SKP1	NM_006930.3	2.8525	0.000133466	0.0135798	6.10648	4.59425
ILMN_1676010	SP1	NM_138473.2	2.1447	0.000556209	0.0386667	6.43591	5.33512
ILMN_2345872	SUMF2	NM_001042470.1	4.6119	0.000153533	0.0150451	6.69027	4.4849
ILMN_1714623	TOMM22	NM_020243.4	1.6265	0.000275435	0.0238256	4.96276	4.26099
ILMN_2383693	UPF2	NM_080599.1	2.9799	0.000327265	0.0270743	5.85966	4.28439
ILMN_2307903	VCAM1	NM_001078.2	8.1288	0.000572422	0.0394915	7.1527	4.12966
ILMN_1728512	YWHAH	NM_003405.3	1.8359	0.000744399	0.0480979	5.80743	4.93096

Supplementary Table 2:

List of List of HuR targets (fold-change >1.5; adjusted p-value<0.05) identified after RIP-chip in MPNST samples

ProbeID	SYMBOL	Acc_No	Fold Change	P-Value	Adjusted P-Value	Mean Signal (HuR IP)	Mean Signal (IgG IP)
ILMN_1856634		BX537823	3.3464	3.65E-06	0.00102439	6.47719	4.73456
ILMN_2152131	ACTB	NM_001101.2	33.5269	1.28E-09	2.00E-06	11.25032	6.18307
ILMN_1657283	ALKBH5	NM_017758.3	5.3254	2.93E-06	0.000893711	7.36541	4.95253
ILMN_2226304	ANKRD50	NM_020337.1	2.3485	2.44E-05	0.00423377	5.51789	4.28613
ILMN_1728471	ARFGEF1	NM_006421.3	2.7533	2.70E-07	0.000134985	6.43034	4.9692
ILMN_1703477	ARHGEF2	NM_004723.2	5.0208	1.16E-05	0.00232117	7.78286	5.45495
ILMN_1759915	ARPC1A	NM_006409.2	2.1163	8.43E-06	0.0018391	6.02477	4.94321
ILMN_2059505	ARPP19	NM_006628.4	2.1087	3.46E-05	0.0052232	5.99567	4.91929
ILMN_3245057	ASAP1	NM_018482.2	3.2869	2.82E-06	0.000871204	7.0227	5.30596
ILMN_2358783	ASB3	NM_145863.1	2.0208	3.11E-05	0.00485799	6.02832	5.01339
ILMN_2188204	ATG12	NM_004707.2	5.2892	4.23E-07	0.00019029	8.76965	6.36661
ILMN_1704452	BCL9	NM_004326.2	2.0724	4.94E-10	8.75E-07	6.11812	5.06681
ILMN_1758918	BRD2	NM_005104.2	2.8365	1.46E-06	0.000517486	7.00414	5.50005
ILMN_1666208	C14orf106	NM_018353.3	3.3895	1.13E-08	9.62E-06	6.38886	4.62778
ILMN_1708906	C2orf29	NM_017546.3	2.5643	8.32E-06	0.0018391	6.41047	5.05193
ILMN_1671116	C3orf21	NM_152531.3	4.2401	4.33E-09	4.60E-06	6.41207	4.32796
ILMN_1777318	C9orf64	NM_032307.3	3.0706	5.88E-06	0.00148677	6.88354	5.26502
ILMN_1736256	CALR	NM_004343.2	2.3752	2.88E-05	0.00470884	5.9734	4.72535
ILMN_1768870	CAPZA2	NM_006136.2	5.6467	8.88E-11	2.14E-07	7.25615	4.75875
ILMN_1803429	CD44	NM_001001391.1	3.1172	1.12E-05	0.00228567	6.24041	4.60016
ILMN_1693014	CEBPB	NM_005194.2	2.5667	2.88E-05	0.00470884	6.18436	4.82447
ILMN_1738075	CMIP	NM_030629.1	27.095	1.53E-13	4.06E-09	11.12037	6.36042
ILMN_1782439	CNN3	NM_001839.2	2.9213	4.55E-05	0.00653817	6.52734	4.98071
ILMN_1706643	COL6A3	NM_057165.2	4.4282	5.60E-05	0.00755051	7.90028	5.75358
ILMN_1782788	CSDA	NM_003651.3	8.6287	1.28E-06	0.000470953	8.0511	4.94196
ILMN_1785988	CSNK1A1	NM_001025105.1	2.4856	3.17E-05	0.00488742	5.75708	4.44347
ILMN_2415235	CSNK1E	NM_152221.2	3.161	8.16E-05	0.00970999	7.39674	5.73635
ILMN_1728478	CXCL16	NM_022059.1	7.3705	1.67E-10	3.41E-07	7.67744	4.79568
ILMN_1684321	CYB5B	NM_030579.2	2.1029	7.47E-06	0.0017111	6.22329	5.15089
ILMN_1658411	CHD4	NM_001273.2	2.3766	2.59E-06	0.000811862	5.6679	4.41901
ILMN_1679428	CHIC2	NM_012110.2	2.03	5.81E-05	0.00771731	5.67924	4.65778
ILMN_1791576	CHSY1	NM_014918.3	2.5091	3.02E-05	0.00480487	5.9864	4.65921
ILMN_2128428	DAB2	NM_001343.1	4.75	7.60E-08	5.17E-05	8.15316	5.90522
ILMN_1811648	DCAKD	NM_024819.3	6.7993	2.41E-09	3.05E-06	7.51731	4.75192
ILMN_1777340	DDX6	NM_004397.3	2.6962	3.00E-05	0.00480487	5.84962	4.4187
ILMN_1770127	DNAJA2	NM_005880.2	13.0177	3.93E-12	3.48E-08	8.40965	4.70725
ILMN_1672503	DPYSL2	NM_001386.4	2.415	5.26E-06	0.00135718	6.04739	4.77535
ILMN_1783448	DYNC1LI2	NM_006141.2	2.2771	6.83E-06	0.0016193	6.3212	5.13397
ILMN_1706502	EIF2AK2	NM_002759.1	4.4334	9.21E-07	0.000364922	7.49883	5.35042
ILMN_1789596	ETV6	NM_001987.4	2.1071	3.09E-07	0.000143819	6.01276	4.93751
ILMN_1727041	EWSR1	NM_005243.2	2.3056	1.84E-08	1.36E-05	6.27691	5.07178
ILMN_3272378	EZR	NM_003379.4	5.6588	1.96E-06	0.000666425	8.5461	6.04561
ILMN_2189870	FCF1	NM_015962.4	2.2018	2.51E-07	0.000128541	6.41618	5.27746
ILMN_1703330	FEM1C	NM_020177.2	4.762	2.68E-09	3.23E-06	7.10035	4.84878
ILMN_1673682	GATAD2A	NM_017660.2	2.4396	2.15E-06	0.000703007	6.31002	5.02339
ILMN_1754912	GLE1	NM_001003722.1	3.154	1.70E-05	0.00323	6.55115	4.89395
ILMN_1690268	HNRPUL1	NM_144732.1	3.6088	6.98E-06	0.00164054	6.39163	4.54009
ILMN_1687384	IFI6	NM_022873.2	4.28	7.65E-05	0.00941662	8.18502	6.0874
ILMN_1723467	ITGB1	NM_002211.2	7.7939	6.82E-06	0.0016193	8.75759	5.79525
ILMN_1810214	JUND	NM_005354.3	31.2964	6.12E-12	4.06E-08	11.68119	6.71327
ILMN_2130411	KDELR1	NM_006801.2	2.2904	5.98E-06	0.00149933	5.96425	4.76862
ILMN_1673936	KHSRP	NM_003685.2	6.0881	1.42E-09	2.10E-06	8.20917	5.60317
ILMN_1703949	KPNB1	NM_002265.4	4.4417	2.26E-08	1.62E-05	7.64208	5.49098
ILMN_1811104	KTELC1	NM_020231.3	3.3128	7.11E-09	7.00E-06	6.94049	5.21242
ILMN_3176090	LOC100130919	XM_001722872.1	2.0934	7.66E-05	0.00941662	6.56501	5.49916
ILMN_3285198	LOC389168	XR_039278.1	2.2366	1.24E-05	0.00245199	6.20904	5.04773
ILMN_3235221	LOC644936	NR_004845.1	2.8016	7.27E-06	0.00167942	6.15759	4.67133
ILMN_1704750	LOC647000	XM_929980.2	3.2227	8.19E-05	0.00970999	7.47598	5.78771
ILMN_1660775	LOC650152	XR_018707.1	2.5312	3.24E-06	0.000954982	6.17069	4.83085
ILMN_1780861	LOC653506	XM_927769.1	5.9352	2.19E-09	2.91E-06	7.13196	4.56268
ILMN_1757106	Mar-06	NM_005885.2	12.7956	3.35E-11	1.27E-07	9.30957	5.63199
ILMN_2224143	MCM3	NM_002388.3	2.1254	2.01E-05	0.00360552	5.89972	4.81197
ILMN_1777526	MED20	NM_004275.3	2.8373	2.10E-06	0.000696406	6.27638	4.77187
ILMN_1746408	MIDN	NM_177401.4	2.7654	7.90E-09	7.24E-06	6.54423	5.07674
ILMN_1773763	MTA2	NM_004739.2	2.5607	6.93E-08	4.84E-05	5.93684	4.58032

ILMN_1814230	MTCP1	NM_014221.3	2.1598	4.69E-07	0.000204393	5.74411	4.63321
ILMN_1735180	NCSTN	NM_015331.2	4.6159	3.70E-06	0.00102439	6.53824	4.33164
ILMN_1724907	NUDT3	NM_006703.2	8.2794	4.53E-11	1.50E-07	7.345	4.29546
ILMN_2330495	OCIAD1	NM_001079842.1	2.2521	1.06E-06	0.000408703	8.89082	7.71957
ILMN_1759154	PABPN1	NM_004643.1	7.1603	6.05E-11	1.62E-07	8.09356	5.25354
ILMN_1686871	PARP1	NM_001618.2	6.4372	1.27E-07	7.86E-05	8.07222	5.38578
ILMN_1682699	PBX2	NM_002586.4	5.4135	2.05E-07	0.000109063	7.23138	4.79481
ILMN_1673215	PCBP1	NM_006196.2	3.4026	2.79E-05	0.00465267	6.53557	4.76893
ILMN_2312296	PCBP2	NM_005016.3	5.8435	1.58E-05	0.00302052	7.86208	5.31524
ILMN_2151817	PFN1	NM_005022.2	7.7159	2.60E-06	0.000811862	9.04984	6.102
ILMN_1802905	PIAS4	NM_015897.2	2.2836	7.86E-05	0.00946314	6.844	5.65271
ILMN_1771599	PLOD2	NM_000935.2	5.7946	1.30E-08	1.04E-05	7.89239	5.35768
ILMN_3241758	POTEF	NM_001099771.1	23.9473	6.10E-11	1.62E-07	9.55731	4.97552
ILMN_1810467	PPP2R1A	NM_014225.3	4.4114	9.64E-10	1.60E-06	7.70878	5.56754
ILMN_3248975	PPP4C	NM_002720.1	2.9817	3.58E-07	0.000163715	6.77989	5.20376
ILMN_1664921	PPP6C	NM_002721.3	3.5373	9.37E-07	0.000365971	6.16062	4.33796
ILMN_1769517	PRKDC	NM_001081640.1	2.8993	1.16E-08	9.62E-06	6.19921	4.66349
ILMN_1745329	PRR14	NM_024031.2	2.4214	2.01E-05	0.00360552	6.23207	4.95624
ILMN_2392674	PRR3	NM_001077497.1	2.6228	1.26E-07	7.86E-05	5.96881	4.57769
ILMN_1720926	PSMD5	NM_005047.2	2.1094	6.18E-06	0.001534	5.17558	4.09872
ILMN_2353202	PTK7	NM_152880.2	6.2426	3.57E-09	3.96E-06	7.20658	4.56443
ILMN_2338997	PTP4A2	NM_080392.2	7.0424	7.12E-06	0.00165933	10.4136	7.59754
ILMN_1752582	RAB5B	NM_002868.2	4.8978	4.77E-07	0.000204423	7.02964	4.73752
ILMN_1760858	RAB8A	NM_005370.4	2.7263	8.48E-06	0.0018391	5.5911	4.14417
ILMN_2109156	RANBP1	NM_002882.2	2.3426	2.52E-07	0.000128541	6.25827	5.03016
ILMN_1700604	RBM14	NM_006328.2	2.5966	1.87E-07	0.000101163	6.52936	5.15275
ILMN_1666739	RBM15	NM_022768.4	2.0417	5.13E-05	0.00713255	5.53003	4.50024
ILMN_1743104	RBM4B	NM_031492.2	2.4582	3.34E-06	0.000968841	5.86935	4.57173
ILMN_1720124	RCC2	NM_018715.1	2.4271	4.49E-05	0.00647915	6.45306	5.1738
ILMN_1661002	RFWD2	NM_022457.5	2.5053	6.72E-06	0.0016193	5.90019	4.57518
ILMN_1665877	RNF149	NM_173647.2	2.2438	3.04E-05	0.00480755	6.14723	4.98125
ILMN_1721842	RYBP	NM_012234.4	2.5308	7.83E-05	0.00946314	5.90721	4.56762
ILMN_1735360	SDAD1	NM_018115.2	4.1656	4.56E-07	0.000201862	6.71387	4.65535
ILMN_1784238	SEC22B	NM_004892.4	4.1283	1.40E-07	8.48E-05	6.72886	4.68332
ILMN_1751028	SERPINH1	NM_001235.2	2.749	3.37E-05	0.00511413	6.42455	4.96566
ILMN_1697469	SFRS6	NM_006275.4	2.7127	3.90E-06	0.00106823	6.74365	5.3039
ILMN_1808501	SH3KBP1	NM_031892.1	2.1703	6.98E-05	0.00874215	6.14177	5.02385
ILMN_3229770	SKP1	NM_006930.3	2.3636	7.98E-06	0.00179635	6.00723	4.76621
ILMN_2191167	SLC30A4	NM_013309.4	3.5357	1.72E-07	9.74E-05	5.88118	4.05916
ILMN_1789999	SLC30A7	NM_133496.3	2.2806	2.19E-05	0.00385532	6.39097	5.20153
ILMN_1676010	SP1	NM_138473.2	2.5408	3.36E-06	0.000968841	6.65313	5.30783
ILMN_2181432	SPC24	NM_182513.1	2.9727	1.16E-05	0.00232117	7.0389	5.46714
ILMN_1804277	SPRED1	NM_152594.1	2.407	8.52E-06	0.0018391	5.96026	4.69303
ILMN_2089329	SPRY2	NM_005842.2	3.1434	7.98E-06	0.00179635	5.72165	4.06934
ILMN_1697670	SRRM1	NM_005839.3	4.1051	3.16E-09	3.65E-06	7.62499	5.58759
ILMN_1711383	STK4	NM_006282.2	2.6749	2.83E-07	0.00013683	7.14158	5.72209
ILMN_1663002	STOML2	NM_013442.1	2.3391	3.26E-05	0.00498087	5.42767	4.20172
ILMN_2345872	SUMF2	NM_001042470.1	3.0667	1.14E-05	0.00231789	6.4459	4.82919
ILMN_1656399	TCEAL8	NM_001006684.1	2.7689	8.92E-06	0.00191051	5.79541	4.32612
ILMN_2368068	TCF20	NM_181492.1	3.3175	9.95E-08	6.61E-05	6.24922	4.51913
ILMN_1814657	TFAP4	NM_003223.1	2.9978	2.90E-07	0.00013759	6.34641	4.7625
ILMN_1793829	TMCO1	NM_019026.2	2.432	1.75E-08	1.36E-05	6.22805	4.94591
ILMN_1792508	TMEM59	NM_004872.3	5.9435	6.70E-09	6.85E-06	7.88695	5.31563
ILMN_1710962	TMEM97	NM_014573.2	2.6591	7.63E-05	0.00941662	6.10159	4.69065
ILMN_1683271	TMSB4X	NM_021109.2	8.9789	2.17E-06	0.000703007	11.99719	8.83064
ILMN_1656066	TNPO2	NM_013433.3	3.3247	1.52E-05	0.00294192	6.94333	5.2101
ILMN_1692731	TTYH3	NM_025250.2	2.6397	5.17E-06	0.00134681	6.52891	5.12853
ILMN_1814789	UBAP2L	NM_014847.2	3.4089	4.46E-06	0.00119671	6.57599	4.8067
ILMN_2383693	UPF2	NM_080599.1	3.0929	5.36E-07	0.000219058	6.04757	4.41862
ILMN_3236765	UPLP	NM_001114403.1	2.8968	1.33E-05	0.00261526	7.13617	5.60172
ILMN_2307903	VCAM1	NM_001078.2	6.8835	4.84E-05	0.00683717	7.2229	4.43976
ILMN_1777220	VCP	NM_007126.2	2.4673	1.05E-05	0.00219701	5.59033	4.28741
ILMN_1795937	VIL2	NM_003379.3	4.361	2.61E-05	0.00441995	7.28471	5.16003
ILMN_2104106	XPR1	NM_004736.2	2.557	1.84E-08	1.36E-05	6.3242	4.96974
ILMN_2252136	YWHAE	NM_006761.3	2.0795	6.67E-05	0.00857697	4.8367	3.78048
ILMN_1728512	YWHAH	NM_003405.3	2.1658	1.17E-06	0.000437019	6.16302	5.04815
ILMN_1656413	ZMPSTE24	NM_005857.3	2.4151	6.70E-07	0.0002697	5.97797	4.70588
ILMN_2150654	ZSWIM4	NM_023072.1	5.066	1.75E-09	2.45E-06	7.14876	4.80791
ILMN_1656676	ZYG11B	NM_024646.1	2.7707	5.40E-05	0.00737348	6.45742	4.98716
ILMN_3237396	AAGAB	NM_024666.3	1.9754	3.69E-06	0.00102439	6.18282	5.20069
ILMN_1665945	ACBD3	NM_022735.3	1.8804	8.04E-05	0.00962108	6.05433	5.14329
ILMN_2095653	AFMID	NM_001010982.1	1.7222	2.92E-05	0.00472192	5.66558	4.8813

ILMN_1703791	ANXA7	NM_004034.1	2.4138	0.000306208	0.0255712	5.45635	4.18503
ILMN_3307651	APOBEC3D	NM_152426.3	1.6163	0.00026119	0.0229675	5.2977	4.60497
ILMN_1768394	ARPC5	NM_005717.2	2.05	0.000345984	0.028012	6.15208	5.11646
ILMN_1658071	ATP1B1	NM_001677.3	2.0572	0.000738762	0.0478502	5.17125	4.13058
ILMN_2140207	ATPBD4	NM_080650.2	1.949	3.18E-05	0.00488742	5.20043	4.23772
ILMN_1725696	ATXN3	NM_004993.4	1.7031	0.000799199	0.0496695	4.97492	4.20675
ILMN_1651826	BASP1	NM_006317.3	2.81	0.000334357	0.0274049	7.29015	5.79961
ILMN_2255133	BCL11A	NM_022893.2	3.2343	0.000111785	0.0120401	5.70649	4.01304
ILMN_1711543	C14orf169	NM_024644.2	1.5877	6.69E-05	0.00857697	4.84686	4.17993
ILMN_1690442	C18orf45	NM_032933.4	2.3366	0.000257846	0.0228245	5.69744	4.47304
ILMN_1812688	C2orf18	NM_017877.3	1.6913	0.000416179	0.0316153	4.95532	4.19716
ILMN_1695917	C5orf15	NM_020199.1	2.2463	0.000128759	0.0133686	5.81768	4.65015
ILMN_1669831	C6orf192	NM_052831.2	1.9409	0.000392953	0.0304235	5.64608	4.68934
ILMN_1680132	CADM1	NM_014333.3	2.583	0.000223381	0.0202461	6.59016	5.22113
ILMN_1685580	CBLB	NM_170662.3	1.583	0.000104132	0.0114744	5.44285	4.78017
ILMN_1667081	CCND2	NM_001759.2	1.6192	0.000533855	0.0374065	5.55065	4.85537
ILMN_2261784	CCNY	NM_145012.3	1.978	7.88E-05	0.00946314	6.18876	5.20469
ILMN_1799688	CDC23	NM_004661.3	1.6676	7.74E-05	0.00946314	5.48688	4.74915
ILMN_1778557	CDC2L5	NM_003718.3	1.8146	4.77E-06	0.00126759	5.38015	4.5205
ILMN_1710326	CLDND1	NM_001040181.1	1.8927	0.000666195	0.0443395	5.28488	4.36441
ILMN_1662328	CNNM3	NM_017623.4	1.7968	0.000296495	0.0250605	5.52088	4.67544
ILMN_1701308	COL1A1	NM_000088.3	5.6806	0.000525879	0.0370431	9.13747	6.63143
ILMN_1729117	COL5A2	NM_000393.3	3.0516	0.000210326	0.0194614	6.3581	4.74854
ILMN_1751615	COQ10B	NM_025147.3	1.8031	0.000132444	0.0135736	5.80014	4.94966
ILMN_2385161	CUL4B	NM_001079872.1	1.5584	0.000263602	0.023027	5.36635	4.7263
ILMN_2106902	CHES1	NM_005197.2	2.1465	0.000575735	0.0396094	5.18279	4.08082
ILMN_1666503	DENND2A	NM_015689.2	2.0371	9.16E-05	0.0104813	5.88458	4.85804
ILMN_1785356	DENND5A	NM_015213.2	2.2698	0.000485364	0.0351465	5.86382	4.68126
ILMN_1768595	DLG4	NM_001365.2	1.8926	0.000365515	0.0292368	5.75716	4.83677
ILMN_1753243	DNAJB11	NM_016306.4	1.9595	0.000491929	0.0354222	6.373	5.40249
ILMN_2374244	DYRK2	NM_003583.2	2.0731	0.000173817	0.0166639	6.48791	5.4361
ILMN_1761463	EFHD2	NM_024329.4	2.2101	8.81E-05	0.0102644	5.82825	4.68413
ILMN_1665717	EIF2S3	NM_001415.3	2.2639	0.000390243	0.030373	5.63673	4.45793
ILMN_1794522	EIF5A	NM_001970.3	3.355	0.000220513	0.0201929	6.54578	4.79946
ILMN_1764873	ELAVL1	NM_001419.2	1.7215	0.000381601	0.0299817	5.18477	4.40114
ILMN_1784320	ELMO1	NM_014800.9	1.7674	4.97E-05	0.00696679	5.65634	4.83472
ILMN_2214910	EPHB4	NM_004444.4	1.8265	3.07E-05	0.00483122	4.85768	3.98859
ILMN_2352131	ERBB2	NM_004448.2	1.7866	5.58E-05	0.00755051	5.2911	4.45386
ILMN_1739222	ETV5	NM_004454.1	1.7066	1.08E-05	0.00224636	5.09009	4.319
ILMN_1746314	EVI5	NM_005665.4	1.8749	0.000177682	0.0168519	6.64986	5.74301
ILMN_1719985	FEM1A	NM_018708.2	1.7904	2.00E-05	0.00360552	5.85613	5.01582
ILMN_1764314	FGD1	NM_004463.2	2.2121	0.000103039	0.0114012	5.7305	4.58505
ILMN_1805796	FLYWCH2	NM_138439.1	1.5417	0.000733816	0.0477628	5.04484	4.42036
ILMN_1736510	FOXN2	NM_002158.3	1.9942	5.06E-06	0.00133103	5.29099	4.29515
ILMN_1748473	GIMAP4	NM_018326.2	1.7518	5.83E-06	0.00148677	5.97391	5.16507
ILMN_1652631	GLIPR2	NM_022343.2	2.6341	0.000802389	0.0496695	5.98038	4.58309
ILMN_1750130	GSPT1	NM_002094.2	1.5547	0.00012962	0.0133686	5.56077	4.92412
ILMN_1705570	H2AFY2	NM_018649.2	2.5363	0.000801881	0.0496695	5.36617	4.02345
ILMN_1767747	HDAC2	NM_001527.2	3.0816	0.000175373	0.0167525	6.43941	4.81574
ILMN_1804150	HIBADH	NM_152740.2	2.0674	0.000185946	0.0175106	5.63362	4.58582
ILMN_2087646	HLX	NM_021958.2	1.7672	0.000596551	0.0408299	4.31531	3.49384
ILMN_2321451	HNRNPD	NM_031369.2	1.9852	2.70E-05	0.00454353	6.46674	5.47745
ILMN_3246409	HNRNPH1	NM_005520.2	1.5753	0.000100228	0.0111452	4.96976	4.31414
ILMN_1719975	HOXC4	NM_014620.4	1.7493	0.000467872	0.0346095	4.89402	4.08721
ILMN_1709882	ICK	NM_016513.3	1.7047	1.11E-05	0.0022805	5.43407	4.66456
ILMN_1752283	ITCH	NM_031483.3	1.6161	6.83E-05	0.00864998	5.58659	4.89408
ILMN_1668535	JOSD1	NM_014876.3	1.6355	0.000772996	0.0487593	4.95892	4.24916
ILMN_1682572	KIAA0528	NM_014802.1	1.9169	0.000164889	0.0160396	5.65687	4.71806
ILMN_1743034	KIF1B	NM_183416.2	1.8192	1.90E-05	0.00351093	6.06065	5.19738
ILMN_1712452	KIF20B	NM_016195.2	1.5432	8.99E-05	0.0103814	5.74343	5.11751
ILMN_1702279	KIF3B	NM_004798.2	1.7393	1.51E-06	0.000528153	5.3195	4.52098
ILMN_1735930	KLF2	NM_016270.2	1.8642	0.000387125	0.0303259	5.73992	4.84137
ILMN_2400448	L3MBTL3	NM_001007102.1	1.8815	0.00012988	0.0133686	4.34049	3.42858
ILMN_1782292	LAMP1	NM_005561.2	1.7038	0.000124012	0.0131731	5.85564	5.0869
ILMN_1733390	LARP1B	NM_032239.2	1.9107	3.75E-05	0.00552441	5.02262	4.08853
ILMN_1774890	LAS1L	NM_031206.3	1.8911	2.23E-06	0.000714537	5.78276	4.86351
ILMN_2129563	LDLRAD3	NM_174902.2	1.6715	0.00080194	0.0496695	4.87865	4.13751
ILMN_3282321	LOC643336	XM_001718563.1	1.5902	0.00064245	0.0431922	5.13678	4.46761
ILMN_1697024	LOC730432	XM_001125680.1	1.7285	0.000110577	0.0119856	5.63018	4.84067
ILMN_2216265	LONP2	NM_031490.2	1.9847	0.000572534	0.0394915	5.29719	4.30825
ILMN_2218450	LSM1	NM_014462.1	1.7208	0.000601984	0.0409904	5.93575	5.15267
ILMN_1702698	LSM11	NM_173491.2	1.5285	0.000413899	0.0316153	5.70175	5.08964

ILMN_2092693	LSM12	NM_152344.1	2.3464	0.00024035	0.0214907	5.59457	4.36409
ILMN_2079803	LSM14A	NM_015578.1	2.4643	0.000716404	0.047229	6.14122	4.84002
ILMN_2162972	LYZ	NM_000239.1	1.6158	5.41E-05	0.00737348	5.31995	4.62766
ILMN_1723020	MAP3K1	NM_005921.1	1.9026	2.81E-05	0.00466499	5.81422	4.88628
ILMN_1807042	MARCKS	NM_002356.5	2.9885	0.00015578	0.0152092	6.61465	5.03522
ILMN_1745699	METTL2A	NM_181725.2	1.686	0.000514724	0.0364507	5.14229	4.3887
ILMN_2347068	MKNK2	NM_017572.2	1.7554	3.63E-05	0.00544914	5.60153	4.78975
ILMN_1775744	MRPS16	NM_016065.3	1.9301	2.13E-05	0.00376827	5.59491	4.64626
ILMN_1716678	NPC2	NM_006432.3	2.3474	0.000451273	0.0337578	6.18105	4.94997
ILMN_2079786	NUAK1	NM_014840.2	1.9743	6.84E-05	0.00864998	5.62453	4.64316
ILMN_1706376	OSBP	NM_002556.2	1.6657	0.000738487	0.0478502	5.74569	5.00956
ILMN_1746618	PAQR7	NM_178422.4	1.5914	0.000779676	0.0490642	4.6613	3.99099
ILMN_2216852	PGK1	NM_000291.2	2.2148	9.38E-05	0.0106405	5.56019	4.41303
ILMN_1733666	PLDN	NM_012388.2	1.5989	0.000438723	0.0330049	5.28068	4.60358
ILMN_2277252	PPFIBP1	NM_003622.2	1.6267	0.000276907	0.0238577	5.84656	5.1446
ILMN_2405018	PPP1CB	NM_206876.1	1.5367	0.000136249	0.0137055	4.78531	4.1655
ILMN_1722858	PPP2CA	NM_002715.2	1.8211	9.60E-06	0.00203912	6.42689	5.56208
ILMN_1759954	PTMA	NM_001099285.1	1.5232	0.000342204	0.027876	4.82149	4.21438
ILMN_1757552	PTRF	NM_012232.3	2.9793	0.000114465	0.012257	6.23824	4.66326
ILMN_1712312	RAB11A	NM_004663.3	2.3054	0.000555235	0.0386667	6.09486	4.88987
ILMN_1768117	RBM25	NM_021239.1	1.6394	3.67E-05	0.00547714	5.65248	4.93928
ILMN_1753008	REXO1	NM_020695.3	1.8602	0.000111986	0.0120401	5.4718	4.57635
ILMN_1801441	RFTN2	NM_144629.1	1.6485	0.000389694	0.030373	4.70658	3.98545
ILMN_1802205	RHOB	NM_004040.2	1.7173	6.08E-05	0.00797138	5.2034	4.42323
ILMN_1714809	RPIA	NM_144563.2	1.5697	0.00018861	0.0176987	5.58108	4.93058
ILMN_1660533	RPN1	NM_002950.3	1.8571	0.000761451	0.0482604	6.00175	5.1087
ILMN_1808157	RUNDC3B	NM_138290.1	1.8488	0.000391157	0.030373	5.64639	4.75978
ILMN_1674955	SCP2	NM_001007098.1	1.6088	0.000492198	0.0354222	4.62329	3.9373
ILMN_1655595	SERPINE2	NM_006216.2	6.2217	0.000239582	0.0214907	8.47029	5.83299
ILMN_1720513	SETBP1	NM_015559.1	1.562	0.000746989	0.0481037	5.94214	5.29871
ILMN_1795341	SFRS1	NM_001078166.1	2.0394	0.000769558	0.048658	6.20269	5.17458
ILMN_1665538	SKP2	NM_032637.2	1.7242	0.000121438	0.0129514	5.45624	4.67031
ILMN_2053103	SLC40A1	NM_014585.3	2.8943	0.000415269	0.0316153	6.01956	4.48635
ILMN_1706553	SMG7	NM_173156.1	1.7505	0.000168613	0.0163419	5.69741	4.88963
ILMN_2409078	SNHG10	NR_001459.2	1.7586	0.000461709	0.0344414	5.69691	4.88246
ILMN_3247064	SNRNP40	NM_004814.2	1.9202	9.68E-05	0.0109384	5.62079	4.67956
ILMN_1787415	SNX16	NM_022133.2	2.235	0.000132894	0.0135736	5.14876	3.98848
ILMN_1814165	SSBP3	NM_018070.3	1.7398	0.000223252	0.0202461	5.86113	5.06219
ILMN_1655163	STK24	NM_003576.3	2.1717	9.17E-05	0.0104813	5.77943	4.6606
ILMN_2172969	STXBP6	NM_014178.6	1.7616	0.000728097	0.047507	5.40134	4.58449
ILMN_1697793	SYNJ2BP	NM_018373.1	2.2474	0.000654724	0.0437956	6.34224	5.17396
ILMN_1790953	TBCB	NM_001281.2	1.6087	0.000755788	0.0481105	5.85929	5.17336
ILMN_1682781	TEAD2	NM_003598.1	1.518	0.000713382	0.047229	5.85104	5.24883
ILMN_1715661	TFAM	NM_003201.1	1.6526	0.000152328	0.015038	4.87343	4.14868
ILMN_1707124	TFPI	NM_006287.4	1.6988	0.000353295	0.0285171	5.67531	4.91076
ILMN_1651346	TICAM2	NM_021649.3	1.7251	0.000200236	0.0187235	4.89492	4.10822
ILMN_1692511	TMEM106C	NM_024056.2	1.9476	0.000264975	0.023071	4.94809	3.98639
ILMN_2042941	TMEM159	NM_020422.3	1.6557	0.000259596	0.0229031	5.85377	5.12634
ILMN_3240316	TMSL3	NM_183049.2	2.4561	0.000126011	0.0133321	5.96599	4.66962
ILMN_1685005	TNFRSF1A	NM_001065.2	1.6379	0.000181438	0.0171469	5.29888	4.58702
ILMN_1726786	TNRC6B	NM_015088.2	1.5231	0.000749146	0.0481037	5.71986	5.11284
ILMN_1672908	TWIST1	NM_000474.3	1.5097	0.000207432	0.0193283	5.67829	5.08402
ILMN_2368576	UBA52	NM_003333.3	2.4917	0.000797305	0.0496695	5.67055	4.35342
ILMN_2301083	UBE2C	NM_181800.1	2.0893	0.000563111	0.0390443	5.6873	4.62428
ILMN_1707475	UBE2E2	NM_152653.2	1.5778	0.000485102	0.0351465	5.78245	5.12456
ILMN_2360291	UGCGL1	NM_020120.2	2.0844	0.000324667	0.0269433	5.7295	4.66985
ILMN_1729563	UGDH	NM_003359.2	1.7317	3.05E-06	0.000920849	5.92471	5.13256
ILMN_2094587	USP8	NM_005154.2	1.5699	0.000416681	0.0316153	4.9028	4.25216
ILMN_2376625	VHL	NM_198156.1	1.7354	0.000304738	0.0255288	5.11757	4.32232
ILMN_1676448	WDFY1	NM_020830.3	2.4497	0.000171286	0.0165406	6.22699	4.93436
ILMN_1707506	YTHDC1	NM_001031732.2	2.4426	0.000323816	0.0269433	5.76133	4.47291
ILMN_1798533	ZNF22	NM_006963.3	1.7447	3.81E-05	0.00555413	5.94782	5.14484
ILMN_1686968	ZNF362	NM_152493.2	1.7579	0.000511308	0.0363056	5.01153	4.19764
ILMN_1672940	ZNF562	NM_017656.2	2.1707	0.000658986	0.0439699	5.83371	4.71554
ILMN_1702384	ZNF706	NM_016096.3	1.8759	0.000286333	0.0243714	5.6865	4.7789
ILMN_1812856	ZSWIM1	NM_080603.3	2.0535	0.000108729	0.0119315	5.51738	4.47926
ILMN_1777061	ZSWIM6	XM_035299.8	1.8745	1.76E-07	9.74E-05	6.05235	5.14587

Supplementary Table 3:

GSEA report for putative HuR targets identified after RIP-chip in MPNST samples

NAME	SIZE	ES	NES	NOM p-val	FDR q-val	FWER p-val	RANK AT MAX	LEADING EDGE
HALLMARK_G2M_CHECKPOINT	110	0.66091704	2.2007105	0	0	0	1642	tags=40%, list=15%, signal=47%
HALLMARK_WNT_BETA_CATENIN_SIGNALING	25	0.78778225	2.0969403	0	0	0	522	tags=32%, list=5%, signal=34%
HALLMARK_EPITHELIAL_MESENCHYMAL_TRANSITION	135	0.5895362	2.0109563	0	0	0	1836	tags=36%, list=17%, signal=42%
HALLMARK_MTORC1_SIGNALING	115	0.5973246	1.9983793	0	0	0	1675	tags=43%, list=16%, signal=51%
HALLMARK_UNFOLDED_PROTEIN_RESPONSE	66	0.6169456	1.9491183	0	0	0	1392	tags=38%, list=13%, signal=43%
HALLMARK_UV_RESPONSE_DN	86	0.586251	1.903825	0	7.66E-04	0.004	1199	tags=29%, list=11%, signal=32%
HALLMARK_MITOTIC_SPINDLE	110	0.5334939	1.7825989	0	0.006466026	0.04	1796	tags=32%, list=17%, signal=38%
HALLMARK_PI3K_AKT_MTOR_SIGNALING	61	0.5641523	1.7556522	0.00120919	0.006801055	0.046	1181	tags=30%, list=11%, signal=33%
HALLMARK_PEROXISOME	50	0.55628026	1.6787007	0.004968944	0.018492453	0.131	1263	tags=28%, list=12%, signal=32%
HALLMARK_APOPTOSIS	105	0.49836984	1.6635113	0.003340757	0.020388661	0.157	1374	tags=30%, list=13%, signal=35%
HALLMARK_GLYCOLYSIS	105	0.49525842	1.6402197	0.001119821	0.023897646	0.199	1499	tags=30%, list=14%, signal=35%
HALLMARK_TNFA_SIGNALING_VIA_NFKB	104	0.48929134	1.6331859	0.002252252	0.02339069	0.209	2112	tags=38%, list=20%, signal=46%
HALLMARK_APICAL_JUNCTION	104	0.49044627	1.6322346	0.001116072	0.021929914	0.212	1377	tags=21%, list=13%, signal=24%
HALLMARK_ANGIOGENESIS	24	0.6194995	1.6268386	0.015647227	0.021904958	0.23	800	tags=33%, list=7%, signal=36%
HALLMARK_PROTEIN_SECRETION	51	0.5395127	1.6265352	0.008760951	0.020524971	0.231	1441	tags=31%, list=13%, signal=36%
HALLMARK_KRAS_SIGNALING_UP	97	0.47857574	1.5667709	0.008	0.03583513	0.399	1305	tags=26%, list=12%, signal=29%
HALLMARK_IL2_STAT5_SIGNALING	110	0.45464563	1.5261422	0.012235818	0.051514674	0.533	1918	tags=30%, list=18%, signal=36%
HALLMARK_E2F_TARGETS	103	0.4570279	1.5065614	0.019296255	0.058281112	0.599	2424	tags=39%, list=23%, signal=50%
HALLMARK_MYC_TARGETS_V1	125	0.43638206	1.4668849	0.007608695	0.07842948	0.721	1460	tags=30%, list=14%, signal=34%
HALLMARK_COMPLEMENT	92	0.4429166	1.4631652	0.025287356	0.0766934	0.737	1308	tags=23%, list=12%, signal=26%
HALLMARK_TGF_BETA_SIGNALING	32	0.5188074	1.4178389	0.07493188	0.1104575	0.861	1992	tags=47%, list=19%, signal=57%
HALLMARK_HYPOXIA	121	0.40840784	1.3906022	0.043141592	0.12912361	0.913	1482	tags=26%, list=14%, signal=30%
HALLMARK_INFLAMMATORY_RESPONSE	87	0.41521847	1.3527392	0.06342016	0.16534793	0.962	1697	tags=29%, list=16%, signal=34%
HALLMARK_IL6_JAK_STAT3_SIGNALING	42	0.46002385	1.3494619	0.10602094	0.16181342	0.962	1892	tags=36%, list=18%, signal=43%
HALLMARK_MYOGENESIS	98	0.4137025	1.3434615	0.073578596	0.1631277	0.967	978	tags=16%, list=9%, signal=18%
HALLMARK_MYC_TARGETS_V2	29	0.478168	1.3149215	0.1401099	0.19196516	0.988	1266	tags=28%, list=12%, signal=31%
HALLMARK_ESTROGEN_RESPONSE_EARLY	81	0.4036203	1.3027929	0.08741259	0.19942532	0.992	1992	tags=35%, list=19%, signal=42%
HALLMARK_XENOBIOTIC_METABOLISM	75	0.4054729	1.2860401	0.12880562	0.21394835	0.997	497	tags=11%, list=5%, signal=11%
HALLMARK_UV_RESPONSE_UP	73	0.40425426	1.2719775	0.14285715	0.22587061	0.998	1327	tags=25%, list=12%, signal=28%
HALLMARK_NOTCH_SIGNALING	24	0.48087007	1.2595494	0.1988555	0.23480763	0.998	1854	tags=33%, list=17%, signal=40%
HALLMARK_ESTROGEN_RESPONSE_LATE	83	0.38209093	1.235678	0.16991964	0.25913045	0.999	1820	tags=28%, list=17%, signal=33%
HALLMARK_BILE_ACID_METABOLISM	39	0.42276612	1.2304164	0.20915033	0.25940308	0.999	639	tags=15%, list=6%, signal=16%
HALLMARK_P53_PATHWAY	107	0.36603296	1.2129018	0.17117117	0.27646855	1	1143	tags=17%, list=11%, signal=19%
HALLMARK_APICAL_SURFACE	19	0.4819313	1.2029262	0.27098674	0.282939	1	2122	tags=47%, list=20%, signal=59%
HALLMARK_CHOLESTEROL_HOMEOSTASIS	37	0.40666175	1.148678	0.26778784	0.3601674	1	1160	tags=14%, list=11%, signal=15%
HALLMARK_ANDROGEN_RESPONSE	56	0.37425527	1.1463497	0.28607595	0.35416937	1	2019	tags=32%, list=19%, signal=39%
HALLMARK_FATTY_ACID_METABOLISM	72	0.35421097	1.122274	0.3038741	0.38514337	1	1602	tags=22%, list=15%, signal=26%
HALLMARK_HEDGEHOG_SIGNALING	15	0.47174457	1.119419	0.33333334	0.37997162	1	1916	tags=40%, list=18%, signal=49%
HALLMARK_HEME_METABOLISM	86	0.3279731	1.0723225	0.3787529	0.4474676	1	1965	tags=28%, list=18%, signal=34%
HALLMARK_ADIPOGENESIS	114	0.31826487	1.0704817	0.36574584	0.43867436	1	1848	tags=26%, list=17%, signal=31%
HALLMARK_INTERFERON_GAMMA_RESPONSE	101	0.3143803	1.0297027	0.43249428	0.49463367	1	1439	tags=19%, list=13%, signal=22%
HALLMARK_KRAS_SIGNALING_DN	61	0.2778541	0.87647456	0.6658625	0.7462542	1	2550	tags=26%, list=24%, signal=34%
HALLMARK_COAGULATION	63	0.2773195	0.86546314	0.6816525	0.74789935	1	879	tags=13%, list=8%, signal=14%
HALLMARK_ALLOGRAFT_REJECTION	105	0.24082597	0.79747456	0.8103638	0.8350867	1	1693	tags=19%, list=16%, signal=22%
HALLMARK_REACTIVE_OXIGEN_SPECIES_PATHWAY	27	0.27147785	0.73852396	0.818705	0.8931917	1	902	tags=15%, list=8%, signal=16%
HALLMARK_DNA_REPAIR	84	0.22432286	0.7244512	0.87283236	0.89092064	1	2210	tags=25%, list=21%, signal=31%
HALLMARK_SPERMATOGENESIS	47	0.23561779	0.69741	0.8802548	0.90089965	1	1588	tags=17%, list=15%, signal=20%

Supplementary Table 5:

GSEA report for genes associated with ShCtr-infected cells

NAME	SIZE	ES	NES	NOM p-val	FDR q-val	FWER p-val	RANK AT MAX	LEADING EDGE
CSR_LATE_UP.V1_UP	161	0.6463217	2.026948	0	0	0	7554	tags=58%, list=13%, signal=67%
RPS14_DN.V1_DN	179	0.60663754	1.9231466	0	0	0	5608	tags=47%, list=10%, signal=52%
RB_P107_DN.V1_UP	135	0.6183999	1.8919705	0	0	0	6012	tags=56%, list=11%, signal=62%
PRC2_EZH2_UP.V1_UP	182	0.59619594	1.8788438	0	0	0	3123	tags=38%, list=6%, signal=41%
GCNP_SHH_UP_LATE.V1_UP	173	0.58318806	1.8233163	0	0	0	3248	tags=34%, list=6%, signal=36%
E2F1_UP.V1_UP	179	0.57013875	1.805654	0	0	0	6918	tags=47%, list=12%, signal=53%
HOXA9_DN.V1_DN	183	0.57443297	1.8028847	0	0	0	6728	tags=44%, list=12%, signal=50%
VEGF_A_UP.V1_DN	189	0.5392788	1.7186297	0	7.17E-04	0.009	5838	tags=42%, list=10%, signal=46%
CORDENONSI_YAP_CONSERVED_SIGNATURE	57	0.625357	1.6881859	0	7.86E-04	0.011	3980	tags=47%, list=7%, signal=51%
PRC2_EED_UP.V1_DN	187	0.5278528	1.6847965	0	8.31E-04	0.013	7405	tags=42%, list=13%, signal=48%
RB_P130_DN.V1_UP	126	0.5470712	1.634776	0	0.00160963	0.027	3464	tags=33%, list=6%, signal=35%
GCNP_SHH_UP_EARLY.V1_UP	163	0.5051882	1.5741572	0	0.003955163	0.069	7276	tags=40%, list=13%, signal=46%
MYC_UP.V1_UP	163	0.49637333	1.5591592	0	0.004583201	0.086	7203	tags=45%, list=13%, signal=51%
BMI1_DN_MEL18_DN.V1_UP	140	0.5100883	1.5514046	0	0.004670326	0.095	7496	tags=51%, list=13%, signal=58%
ESC_J1_UP_LATE.V1_DN	179	0.4785535	1.5299935	0	0.006708479	0.136	7240	tags=40%, list=13%, signal=46%
ERB2_UP.V1_DN	185	0.44723895	1.4340094	0	0.018547483	0.358	6705	tags=36%, list=12%, signal=40%
STK33_DN	263	0.4047926	1.3371545	0.002873563	0.05775106	0.765	6610	tags=36%, list=12%, signal=41%
STK33_SKM_DN	257	0.3825717	1.2545364	0.01907357	0.13202493	0.974	3143	tags=27%, list=6%, signal=28%
BMI1_DN.V1_UP	142	0.40230384	1.246761	0.033254158	0.13612205	0.984	3771	tags=29%, list=7%, signal=31%
MTOR_UP.V1_UP	161	0.40020773	1.2454468	0.05707196	0.13068776	0.984	5268	tags=29%, list=9%, signal=31%
RB_DN.V1_UP	130	0.4052772	1.2372407	0.0530504	0.1349215	0.991	7074	tags=39%, list=13%, signal=45%
MEL18_DN.V1_UP	136	0.4008568	1.2277946	0.07263923	0.14207262	0.994	6674	tags=39%, list=12%, signal=44%
LTE2_UP.V1_DN	190	0.3812306	1.2164211	0.036458332	0.15159744	0.996	5911	tags=31%, list=10%, signal=35%
HINATA_NFKB_IMMU_INF	16	0.604309	1.2118691	0.1971831	0.15200374	0.997	5674	tags=50%, list=10%, signal=56%
E2F3_UP.V1_UP	175	0.38684732	1.2116122	0.06806283	0.14621256	0.997	6742	tags=37%, list=12%, signal=42%
SRC_UP.V1_DN	160	0.3809992	1.195038	0.078085646	0.16436355	1	7583	tags=34%, list=13%, signal=39%
TGFB_UP.V1_UP	184	0.37824148	1.1847157	0.06818182	0.17407961	1	6971	tags=30%, list=12%, signal=34%
JNK_DN.V1_DN	184	0.3669385	1.1626757	0.08163265	0.20641711	1	6133	tags=28%, list=11%, signal=32%
STK33_NOMO_DN	260	0.35340944	1.1574258	0.07520892	0.20890447	1	5207	tags=29%, list=9%, signal=32%
NFE2L2.V2	443	0.33444002	1.1482269	0.043209877	0.21954389	1	5163	tags=23%, list=9%, signal=25%
LEF1_UP.V1_UP	189	0.35781455	1.1402278	0.13054188	0.22859871	1	6613	tags=34%, list=12%, signal=38%
KRAS.AMP.LUNG_UP.V1_DN	137	0.36763945	1.1303136	0.1598063	0.24194734	1	5172	tags=25%, list=9%, signal=27%
PRC2_SUZ12_UP.V1_UP	180	0.36056408	1.129645	0.1484375	0.23572789	1	5801	tags=26%, list=10%, signal=28%
P53_DN.V1_UP	188	0.3551615	1.1239165	0.14285715	0.24101885	1	6313	tags=32%, list=11%, signal=36%
NRL_DN.V1_DN	130	0.3683067	1.1220392	0.17085427	0.23828094	1	6219	tags=32%, list=11%, signal=36%
ATF2_S_UP.V1_UP	184	0.35174403	1.1180388	0.15167095	0.23962294	1	7869	tags=35%, list=14%, signal=41%
IL15_UP.V1_UP	180	0.34741217	1.0986463	0.20408164	0.27593216	1	5079	tags=26%, list=9%, signal=28%
EGFR_UP.V1_DN	188	0.33923462	1.0757606	0.21505377	0.3239593	1	2944	tags=22%, list=5%, signal=23%
TBK1.DN.48HRS_DN	50	0.41038764	1.0738732	0.3272311	0.32051444	1	7426	tags=38%, list=13%, signal=44%
CSR_EARLY_UP.V1_UP	152	0.34655768	1.0694181	0.24939467	0.3240171	1	8190	tags=39%, list=15%, signal=45%
JAK2_DN.V1_UP	174	0.33457637	1.0504899	0.28533334	0.36530247	1	6929	tags=26%, list=12%, signal=30%
SIRNA_EIF4GI_DN	92	0.3565203	1.0272043	0.37588653	0.42244884	1	5997	tags=28%, list=11%, signal=32%
PDGF_ERK_DN.V1_UP	138	0.33252293	1.0162523	0.39099526	0.44549736	1	5762	tags=27%, list=10%, signal=30%
P53_DN.V2_UP	145	0.32177395	0.99758	0.4555256	0.4955497	1	3347	tags=19%, list=6%, signal=20%
BRCA1_DN.V1_DN	133	0.3277704	0.9953412	0.44652405	0.49176452	1	7812	tags=29%, list=14%, signal=33%
KRAS.LUNG.BREAST_UP.V1_UP	138	0.32414296	0.9873838	0.47921762	0.50743544	1	8698	tags=36%, list=15%, signal=43%
CTIP_DN.V1_DN	126	0.3256546	0.9829276	0.45343137	0.5109972	1	6141	tags=27%, list=11%, signal=30%
PRC1_BMI_UP.V1_UP	182	0.30172926	0.9559453	0.58	0.5925697	1	4613	tags=20%, list=8%, signal=22%
BCAT_GDS748_DN	45	0.33781058	0.8585551	0.7276888	0.9142249	1	4760	tags=24%, list=8%, signal=27%
BCAT_BILD_ET_AL_DN	46	0.29015687	0.75080264	0.9326087	0.99237084	1	14542	tags=52%, list=26%, signal=70%

Supplementary Table 6:

GSEA report for genes associated with shHuR#1-infected cells

NAME	SIZE	ES	NES	NOM p-val	FDR q-val	FWER p-val	RANK AT MAX	LEADING EDGE
BMI1_DN_MEL18_DN.V1_DN	140	-0.65756273	-1.9316547	0	0	0	6383	tags=53%, list=11%, signal=59%
MEL18_DN.V1_DN	140	-0.6470315	-1.9101604	0	0	0	6830	tags=54%, list=12%, signal=62%
STK33_UP	273	-0.5978454	-1.888939	0	4.30E-04	0.001	8476	tags=49%, list=15%, signal=58%
STK33_NOMO_UP	272	-0.58234036	-1.8480598	0	3.22E-04	0.001	7740	tags=47%, list=14%, signal=55%
PRC2_EED_UP.V1_UP	182	-0.5965234	-1.8123351	0	2.58E-04	0.001	6805	tags=46%, list=12%, signal=52%
STK33_SKM_UP	268	-0.55316085	-1.7442031	0	0.002238993	0.011	7054	tags=38%, list=13%, signal=43%
BMI1_DN.V1_DN	138	-0.5773672	-1.6887712	0	0.00365305	0.02	6830	tags=43%, list=12%, signal=49%
MTOR_UP.N4.V1_DN	169	-0.55986893	-1.6829	0	0.00351008	0.022	6973	tags=40%, list=12%, signal=45%
ERB2_UP.V1_UP	183	-0.548108	-1.6590604	0	0.004342172	0.03	6402	tags=43%, list=11%, signal=48%
KRAS.KIDNEY_UP.V1_UP	140	-0.56736267	-1.6563841	0.001655629	0.004284753	0.033	2844	tags=38%, list=5%, signal=40%
ATF2_S_UP.V1_DN	180	-0.5472791	-1.6556194	0	0.00389523	0.033	6668	tags=48%, list=12%, signal=55%
IL2_UP.V1_DN	183	-0.53921	-1.6400353	0	0.004720663	0.044	7629	tags=38%, list=14%, signal=44%
CAHOY_ASTROGLIAL	96	-0.5661811	-1.6217597	0	0.005484538	0.056	6804	tags=45%, list=12%, signal=51%
ESC_V6.5_UP_LATE.V1_UP	185	-0.53170925	-1.6210804	0	0.005177034	0.057	6374	tags=40%, list=11%, signal=45%
ATF2_UP.V1_DN	180	-0.53176725	-1.6154956	0	0.00532063	0.062	4385	tags=37%, list=8%, signal=40%
RPS14_DN.V1_UP	187	-0.5262576	-1.6150908	0.001650165	0.00498809	0.062	7404	tags=40%, list=13%, signal=45%
RB_P130_DN.V1_DN	130	-0.55093676	-1.6129909	0	0.004769147	0.063	7701	tags=46%, list=14%, signal=53%
HOXA9_DN.V1_UP	181	-0.53509647	-1.6123956	0	0.004504195	0.063	6915	tags=41%, list=12%, signal=47%
E2F1_UP.V1_DN	183	-0.5256635	-1.5868429	0	0.006698331	0.097	6804	tags=43%, list=12%, signal=48%
CSR_EARLY_UP.V1_DN	139	-0.54012334	-1.5828733	0.001675042	0.00648976	0.099	8014	tags=45%, list=14%, signal=53%
ESC_J1_UP_LATE.V1_UP	186	-0.5178298	-1.566775	0.001620746	0.007163001	0.115	6374	tags=42%, list=11%, signal=47%
AKT_UP.V1_DN	179	-0.510245	-1.5351222	0.001633987	0.010299	0.174	7583	tags=42%, list=13%, signal=48%
LEF1_UP.V1_DN	181	-0.50430536	-1.5283473	0	0.010674264	0.187	6892	tags=45%, list=12%, signal=51%
CSR_LATE_UP.V1_DN	157	-0.5095311	-1.5267802	0	0.010275873	0.188	3803	tags=32%, list=7%, signal=34%
WNT_UP.V1_DN	164	-0.50823104	-1.5252419	0	0.009965336	0.19	5912	tags=36%, list=10%, signal=40%
P53_DN.V1_DN	187	-0.50242114	-1.5183854	0.001666667	0.010564892	0.207	7369	tags=45%, list=13%, signal=52%
PDGF_UP.V1_DN	126	-0.5178902	-1.5111766	0	0.010716772	0.215	6840	tags=36%, list=12%, signal=41%
CRX_DN.V1_DN	131	-0.5105873	-1.5057538	0.001692047	0.010831025	0.224	5824	tags=34%, list=10%, signal=38%
RELA_DN.V1_DN	130	-0.5106599	-1.502233	0	0.010917531	0.233	6327	tags=32%, list=11%, signal=36%
SNF5_DN.V1_DN	156	-0.505419	-1.5021679	0.003174603	0.010553614	0.233	4278	tags=31%, list=8%, signal=33%
IL15_UP.V1_DN	172	-0.4874963	-1.4768474	0.001639344	0.014126416	0.3	4935	tags=26%, list=9%, signal=29%
IL21_UP.V1_DN	172	-0.48531935	-1.4578557	0.004942339	0.018886749	0.397	8422	tags=35%, list=15%, signal=42%
CTIP_DN.V1_UP	130	-0.4980072	-1.4321824	0.003350084	0.025314162	0.505	6327	tags=25%, list=11%, signal=29%
KRAS.LUNG_UP.V1_DN	136	-0.48795864	-1.4268856	0.00174216	0.02614274	0.524	6526	tags=33%, list=12%, signal=37%
PRC1_BMI_UP.V1_DN	177	-0.46813366	-1.4235611	0.00331675	0.026448129	0.537	8339	tags=40%, list=15%, signal=46%
KRAS.600.LUNG.BREAST_UP.V1_DN	274	-0.4455334	-1.4080275	0.001545595	0.032223556	0.609	6536	tags=30%, list=12%, signal=34%
PIGF_UP.V1_UP	187	-0.4598211	-1.4061472	0.009771987	0.03257395	0.622	8646	tags=36%, list=15%, signal=42%
KRAS.LUNG.BREAST_UP.V1_DN	136	-0.47849157	-1.4030654	0.010327023	0.033116005	0.635	6499	tags=34%, list=12%, signal=38%
PKCA_DN.V1_UP	166	-0.4687581	-1.3980176	0.00487013	0.03395229	0.657	7992	tags=39%, list=14%, signal=45%
PRC2_EZH2_UP.V1_DN	183	-0.4541471	-1.3906862	0.003378379	0.036799826	0.695	6425	tags=43%, list=11%, signal=48%
VEGF_A_UP.V1_UP	189	-0.45748687	-1.3872037	0.007898894	0.03729289	0.714	6768	tags=34%, list=12%, signal=39%
EGFR_UP.V1_UP	185	-0.4549511	-1.3788	0.003284072	0.040864076	0.762	7308	tags=45%, list=13%, signal=52%
MEK_UP.V1_UP	188	-0.45373976	-1.378503	0.011217949	0.0401403	0.763	5729	tags=34%, list=10%, signal=38%
PTEN_DN.V2_UP	136	-0.46210852	-1.3765156	0.015463918	0.040344935	0.773	4212	tags=30%, list=7%, signal=33%
ESC_J1_UP_EARLY.V1_UP	168	-0.45925367	-1.3724189	0.006493507	0.041627586	0.781	7031	tags=39%, list=12%, signal=45%
MTOR_UP.V1_DN	178	-0.45521936	-1.3679518	0.011627907	0.043126527	0.796	7705	tags=37%, list=14%, signal=43%
KRAS.50_UP.V1_UP	47	-0.5499441	-1.365234	0.04920914	0.043695517	0.806	6035	tags=43%, list=11%, signal=48%
CAHOY_NEURONAL	95	-0.4838539	-1.3634824	0.021922428	0.043876994	0.815	5980	tags=37%, list=11%, signal=41%
ALK_DN.V1_UP	137	-0.46332282	-1.3590385	0.016920473	0.045335766	0.828	8093	tags=38%, list=14%, signal=44%
MYC_UP.V1_DN	157	-0.4463858	-1.3410349	0.012965964	0.055599816	0.894	6881	tags=34%, list=12%, signal=38%
SIRNA_EIF4GI_UP	91	-0.4781993	-1.3376204	0.04042179	0.057057574	0.91	7772	tags=40%, list=14%, signal=46%
TGFB_UP.V1_DN	183	-0.43953583	-1.3299516	0.014218009	0.06085203	0.928	5341	tags=29%, list=9%, signal=32%
RAF_UP.V1_UP	187	-0.43491796	-1.3236665	0.017271157	0.064317055	0.943	6708	tags=39%, list=12%, signal=44%
AKT_UP.V1_UP	163	-0.4465274	-1.3232546	0.010256411	0.0634152	0.943	5341	tags=29%, list=9%, signal=32%
PRC2_SUZ12_UP.V1_DN	178	-0.43630323	-1.3204952	0.01584786	0.06416449	0.946	7343	tags=33%, list=13%, signal=38%
EIF4E_DN	97	-0.47950608	-1.3200814	0.03691275	0.06344434	0.947	7612	tags=37%, list=14%, signal=43%
ESC_V6.5_UP_EARLY.V1_DN	171	-0.43647343	-1.3151157	0.011382114	0.06571491	0.959	6383	tags=36%, list=11%, signal=41%
JNK_DN.V1_UP	182	-0.43130216	-1.3147677	0.01618123	0.06481554	0.959	6822	tags=31%, list=12%, signal=36%
KRAS.BREAST_UP.V1_DN	138	-0.4474425	-1.3136595	0.031719532	0.06449671	0.961	6421	tags=30%, list=11%, signal=34%
GCNP_SHH_UP_EARLY.V1_DN	164	-0.43535474	-1.3025784	0.032312926	0.072718576	0.974	6259	tags=29%, list=11%, signal=32%
LTE2_UP.V1_UP	180	-0.4305646	-1.3000975	0.014925373	0.07323898	0.976	6681	tags=33%, list=12%, signal=38%
BCAT_BILD_ET_AL_UP	44	-0.5213563	-1.2990202	0.07509158	0.07285976	0.977	6584	tags=39%, list=12%, signal=44%
CYCLIN_D1_KE_.V1_UP	188	-0.42368215	-1.2925098	0.031719532	0.0770856	0.985	7522	tags=35%, list=13%, signal=40%
KRAS.600_UP.V1_DN	275	-0.40660927	-1.2846336	0.009188362	0.08312739	0.988	6570	tags=27%, list=12%, signal=30%
ATM_DN.V1_DN	145	-0.43178657	-1.2720134	0.0539629	0.0936025	0.992	4076	tags=21%, list=7%, signal=23%
MEK_UP.V1_DN	187	-0.4179306	-1.2624791	0.034941763	0.10203829	0.996	8636	tags=37%, list=15%, signal=44%
AKT_UP_MTOR_DN.V1_UP	174	-0.41666472	-1.2565967	0.04399323	0.10667876	0.996	5768	tags=28%, list=10%, signal=31%
GCNP_SHH_UP_LATE.V1_DN	178	-0.4182879	-1.2528129	0.046357617	0.109699294	0.997	8615	tags=38%, list=15%, signal=44%
YAP1_DN	42	-0.50460243	-1.2466412	0.11588785	0.11543524	0.999	5806	tags=33%, list=10%, signal=37%
AKT_UP_MTOR_DN.V1_DN	177	-0.40857023	-1.2437973	0.0539629	0.11731986	0.999	6792	tags=32%, list=12%, signal=36%

CYCLIN_D1_UP.V1_UP	184	-0.40996936	-1.2426367	0.041467305	0.11701133	0.999	7729	tags=32%, list=14%, signal=37%
KRAS.600_UP.V1_UP	267	-0.39482123	-1.2404662	0.031722054	0.11752298	0.999	5107	tags=25%, list=9%, signal=28%
RAF_UP.V1_DN	186	-0.40824127	-1.2385556	0.04639175	0.11826252	0.999	7391	tags=34%, list=13%, signal=39%
RAPA_EARLY_UP.V1_DN	188	-0.4034595	-1.229752	0.05409836	0.12861106	1	8071	tags=40%, list=14%, signal=47%
SNF5_DN.V1_UP	167	-0.4062797	-1.2276385	0.053658538	0.12961918	1	6971	tags=34%, list=12%, signal=39%
ESC_V6.5_UP_EARLY.V1_UP	164	-0.4069866	-1.2189118	0.07559055	0.14075077	1	7791	tags=38%, list=14%, signal=44%
BCAT_GDS748_UP	48	-0.49227807	-1.2180996	0.13186814	0.14029224	1	3250	tags=33%, list=6%, signal=35%
KRAS.KIDNEY_UP.V1_DN	132	-0.41603586	-1.215755	0.08280255	0.14170973	1	6043	tags=24%, list=11%, signal=27%
RAPA_EARLY_UP.V1_UP	170	-0.40015364	-1.2105435	0.06259542	0.14767988	1	8281	tags=32%, list=15%, signal=38%
IL21_UP.V1_UP	179	-0.39640853	-1.1967514	0.07096774	0.16624704	1	5944	tags=30%, list=11%, signal=34%
DCA_UP.V1_DN	172	-0.39655963	-1.1931783	0.08674304	0.16995634	1	8647	tags=34%, list=15%, signal=40%
P53_DN.V2_DN	144	-0.39602318	-1.1818166	0.094527364	0.18789166	1	4017	tags=22%, list=7%, signal=24%
TBK1.DF_UP	274	-0.3752914	-1.1801769	0.0748503	0.1880153	1	6836	tags=35%, list=12%, signal=39%
PTEN_DN.V1_UP	178	-0.39142367	-1.1790795	0.09737249	0.18758051	1	7096	tags=30%, list=13%, signal=34%
CAHOY_ASTROCYTIC	98	-0.4201239	-1.1784772	0.13416816	0.18634637	1	5631	tags=32%, list=10%, signal=35%
GLI1_UP.V1_DN	25	-0.5265167	-1.1754428	0.19855596	0.18937056	1	11987	tags=68%, list=21%, signal=86%
WNT_UP.V1_UP	174	-0.3880947	-1.1652383	0.10981697	0.20461807	1	5463	tags=22%, list=10%, signal=25%
RB_P107_DN.V1_DN	124	-0.40116835	-1.1642183	0.13879599	0.20420375	1	8647	tags=39%, list=15%, signal=46%
KRAS.300_UP.V1_UP	137	-0.4008654	-1.1640077	0.13565217	0.20229357	1	4934	tags=28%, list=9%, signal=30%
CRX_NRL_DN.V1_DN	121	-0.39857513	-1.1579727	0.14262295	0.21177538	1	5320	tags=29%, list=9%, signal=32%
KRAS.DF.V1_DN	188	-0.3757507	-1.1439826	0.14379086	0.23671098	1	5254	tags=24%, list=9%, signal=26%
RB_DN.V1_DN	120	-0.39151672	-1.1425174	0.16440678	0.23713848	1	6315	tags=32%, list=11%, signal=36%
ATM_DN.V1_UP	142	-0.38630423	-1.139042	0.1826923	0.24180397	1	8213	tags=35%, list=15%, signal=41%
BRCA1_DN.V1_UP	130	-0.39138877	-1.1367528	0.17478992	0.2443973	1	6046	tags=18%, list=11%, signal=20%
BCAT.100_UP.V1_DN	39	-0.46870634	-1.1356789	0.2410072	0.24386278	1	5586	tags=23%, list=10%, signal=26%
ESC_J1_UP_EARLY.V1_DN	172	-0.37756443	-1.1302017	0.17462933	0.25283358	1	6792	tags=30%, list=12%, signal=34%
TBK1.DF_DN	271	-0.354238	-1.110956	0.16850394	0.29304767	1	8807	tags=34%, list=16%, signal=40%
KRAS.PROSTATE_UP.V1_DN	140	-0.37569675	-1.107071	0.20921986	0.29909995	1	6090	tags=26%, list=11%, signal=29%
KRAS.AMP.LUNG_UP.V1_UP	134	-0.3755756	-1.1046449	0.22222222	0.30164874	1	11014	tags=37%, list=20%, signal=46%
SINGH_KRAS_DEPENDENCY_SIGNATURE_	20	-0.5250638	-1.1036365	0.31690142	0.3010787	1	5035	tags=30%, list=9%, signal=33%
KRAS.300_UP.V1_DN	136	-0.37628254	-1.1031575	0.22824302	0.29914707	1	8854	tags=34%, list=16%, signal=40%
KRAS.LUNG_UP.V1_UP	134	-0.37086076	-1.0970467	0.22704507	0.31044355	1	4833	tags=18%, list=9%, signal=20%
DCA_UP.V1_UP	177	-0.36171275	-1.0921688	0.24203822	0.3186584	1	7648	tags=28%, list=14%, signal=32%
KRAS.BREAST_UP.V1_UP	135	-0.37284163	-1.0911309	0.2594417	0.31776333	1	6327	tags=27%, list=11%, signal=30%
BCAT.100_UP.V1_UP	48	-0.43644738	-1.0902193	0.28623852	0.31710854	1	5236	tags=31%, list=9%, signal=34%
IL2_UP.V1_UP	181	-0.3569956	-1.0815799	0.252443	0.33586597	1	7391	tags=31%, list=13%, signal=36%
PKCA_DN.V1_DN	160	-0.3623502	-1.0806458	0.26554623	0.33467913	1	7471	tags=27%, list=13%, signal=31%
PTEN_DN.V1_DN	174	-0.35588032	-1.0788217	0.24088748	0.33641726	1	6236	tags=26%, list=11%, signal=29%
MTOR_UP.N4.V1_UP	190	-0.352475	-1.0739225	0.2651391	0.3454366	1	6703	tags=30%, list=12%, signal=34%
NOTCH_DN.V1_DN	177	-0.35606456	-1.0691338	0.277865	0.35474998	1	3906	tags=22%, list=7%, signal=24%
E2F3_UP.V1_DN	139	-0.35568857	-1.0588322	0.312187	0.3780714	1	6324	tags=23%, list=11%, signal=26%
KRAS.DF.V1_UP	184	-0.3455789	-1.0588236	0.30819672	0.37473845	1	7505	tags=35%, list=13%, signal=41%
CYCLIN_D1_KE_.V1_DN	187	-0.34854963	-1.0541956	0.30769232	0.3834846	1	5989	tags=25%, list=11%, signal=27%
CAMP_UP.V1_DN	194	-0.33900616	-1.038234	0.31726283	0.42363483	1	7150	tags=30%, list=13%, signal=34%
CAHOY_OLIGODENDROCUTIC	90	-0.36661395	-1.0233443	0.375	0.46248755	1	5278	tags=28%, list=9%, signal=31%
ESC_V6.5_UP_LATE.V1_DN	177	-0.34053668	-1.0189543	0.4130809	0.47102657	1	4010	tags=21%, list=7%, signal=23%
NOTCH_DN.V1_UP	177	-0.33687726	-1.0186822	0.40097404	0.46767175	1	5873	tags=24%, list=10%, signal=26%
RELA_DN.V1_UP	147	-0.34391066	-1.0186015	0.3993232	0.46389627	1	7564	tags=27%, list=13%, signal=31%
KRAS.600.LUNG.BREAST_UP.V1_UP	274	-0.31640318	-1.0032834	0.43317232	0.5042312	1	6527	tags=24%, list=12%, signal=27%
TBK1.DN.48HRS_UP	50	-0.39224413	-1.0005885	0.45137614	0.50812435	1	7006	tags=34%, list=12%, signal=39%
CYCLIN_D1_UP.V1_DN	186	-0.32892182	-0.9993821	0.45033112	0.50747675	1	5255	tags=23%, list=9%, signal=25%
NRL_DN.V1_UP	131	-0.3396588	-0.9910634	0.45890412	0.52836514	1	3957	tags=19%, list=7%, signal=20%
ATF2_UP.V1_UP	186	-0.3272957	-0.98880756	0.47460318	0.5308636	1	7359	tags=28%, list=13%, signal=33%
CRX_DN.V1_UP	131	-0.33606824	-0.98316747	0.48681897	0.5439377	1	7597	tags=27%, list=13%, signal=32%
PIGF_UP.V1_DN	185	-0.32482016	-0.9805442	0.5109375	0.54723155	1	6518	tags=27%, list=12%, signal=30%
SRC_UP.V1_UP	158	-0.32862654	-0.98052436	0.50161815	0.542925	1	7056	tags=27%, list=13%, signal=31%
KRAS.PROSTATE_UP.V1_UP	131	-0.33539593	-0.9757101	0.5147059	0.5532125	1	5782	tags=26%, list=10%, signal=29%
PDGF_UP.V1_UP	142	-0.32810605	-0.9740319	0.5074135	0.5537849	1	8728	tags=34%, list=15%, signal=40%
PDGF_ERK_DN.V1_DN	144	-0.32522762	-0.95591176	0.5565217	0.6048704	1	8609	tags=34%, list=15%, signal=40%
CAMP_UP.V1_UP	192	-0.31045902	-0.94301784	0.5972222	0.64022213	1	8887	tags=35%, list=16%, signal=42%
PTEN_DN.V2_DN	132	-0.31941798	-0.9429997	0.61435723	0.63538134	1	6248	tags=24%, list=11%, signal=27%
CRX_NRL_DN.V1_UP	134	-0.3177159	-0.9251622	0.66883117	0.68720186	1	7718	tags=27%, list=14%, signal=31%
JAK2_DN.V1_DN	129	-0.31307027	-0.9120055	0.6923077	0.7234041	1	4312	tags=20%, list=8%, signal=22%
EIF4E_UP	92	-0.31982827	-0.89133775	0.7325175	0.78107285	1	6048	tags=21%, list=11%, signal=23%
YAP1_UP	43	-0.35468942	-0.8805186	0.687389	0.8077706	1	7557	tags=33%, list=13%, signal=38%
KRAS.50_UP.V1_DN	45	-0.35012364	-0.8783998	0.6666667	0.80817103	1	12197	tags=42%, list=22%, signal=54%
ALK_DN.V1_DN	132	-0.30028018	-0.8743522	0.77759475	0.81399626	1	9043	tags=32%, list=16%, signal=38%
GLI1_UP.V1_UP	25	-0.32332292	-0.72717	0.9073084	0.9948409	1	6973	tags=28%, list=12%, signal=32%

Supplementary Table 7:
List or Reagents (qPCR primers, Antibodies, Plasmids)

Primers	Forward (5' - 3')	Reverse (5' - 3')
Human ANLN	ATGTCTTCGTGGCCGATTTGA	CTCTGACAGTGAGTTTCCTGTTT
Human BCL-9	GGATTGCGGGAAAAGGGTCT	AACTGGGCCCACATTCAGTC
Human BIRC3	CTTGTCCTTGCTGGTGCAT	AAGAAGTCGTTTTCCTCCTTTGT
Human BIRC5	GCCAGATGACGACCCCATGCAA	CACGGCGCACTTTCTCCGCA
Human BRD2	CGGTTCCTTGCGGTCAAGAT	CAGCAACCCTGCATTCCCTT
Human BRD3	CCATGGTGAGCAAGGGCGCT	GCACGGCCTTCAGCTGCTCC
Human BRD4	AACCTGGCGTTTCCACGGTA	GCCTGCACAGGAGGAGGATT
Human CDK2	CTCCACCGAGACCTTAAACCTCAG	TCGGTACCACAGGGTCACCA
Human CDK4	CTTCTGCAGTCCACATATGCAACA	CAACTGGTCGGCTTCAGAGTTTC
Human CDK6	GATCTCTGGAGTGTTGGCTGCATA	GGCAACATCTCTAGGCCAGTCTTC
Human CTGF	ACCGACTGGAAGACACGTTTG	CCAGGTCAGCTTCGCAAGG
Human CTNNB1	CATCTACACAGTTTGATGCTGCT	GCAGTTTTGTCAGTTCAGGGA
Human CYCLIN D1	AAAATGCCAGAGGCGGAGGAGA	GGAGGGCGGATTGGAAATGAAC
Human CYCLIN D2	CTTCAAGTGCGTGCAGAAGGA	CCAAGAAACGGTCCAGGTAAT
Human CYCLIN E	ACAATAATGCAGTCTGTGCA	ATAGACTTCACACACCTCCA
Human CYR61	TGAAGCGGCTCCCTGTTTT	CGGGTTTCTTTCACAAGGCG
Human E2F1	TATGGTGATCAAAGCCCCTC	AGATGATGGTGGTGGTGACA
Human E2F2	GCCTATGTGACTTACCAGGATATCC	CCTTGACGGCAATCACTGTCT
Human E2F3	GAGACTGAAACACACAGTCC	CCTGAGTTGGTTGAAGCC
Human GAPDH	ACCCACTCCTCCACCTTTGA	CTGTTGCTGTAGCCAAATTCGT
Human HuR	GGTTCGGAGGCCCCGTTCAC	CCAGCCGGAGGAGGCGTTTC
Human p16	GGCACCAGAGGCAGTAACCA	GGACCTTCGGTGACTGATGATCTAA
Human p21	AAGACCATGTGGACCTGTCACTGT	GAAGATCAGCCGGCGTTTG
Human p27	GAAGCCTGGCCTCAGAAGAC	CCATTCCATGAAGTCAGCGAT
Human TAZ	CCATCACTAATAATAGCTCAGATC	GTGATTACAGCCAGGTTAGAAAG
Human TEAD1	GATGATGCTGGGGCTTTTTA	AGGAGCAAACTTTGGTGGAA
Human TEAD2	TTTTGGTCTGGAGGATCTGG	CGTACCCTGGGAGGTCAGTA
Human TEAD3	TCATCCACAAGCTGAAGCAC	AGCAATGACAAGCAGGGTCT
Human TEAD4	TTGCCAGGCCAAGCCGGAAC	CGGGCCCTGCAGGAGACTCA
Human YAP1	ATCCCAGCACAGCAAATTCT	TGGATTTTGAGTCCCACCAT

Antibody	Source	Catalogue Number
Mouse IgG, HRP linked antibody	Cell Signaling	Cat# 7076, RRID:AB_330924
Rabbit IgG, HRP linked antibody	Cell Signaling	Cat# 7074, RRID:AB_2099233
Anti-β-Actin	Sigma-Aldrich	Cat# A5441, RRID:AB_476744
Anti-Aurora A (D3E4Q)	Cell Signaling	Cat# 14475, RRID:AB_2665504
Anti-Aurora B/AIM1	Cell Signaling	Cat# 3094, RRID:AB_2061777
Anti-BRD2	Abcam	Cat# ab139690, RRID:AB_2737409
Anti-BRD3 [2088C3a]	Abcam	Cat# ab50818, RRID:AB_868478
Anti-BRD4 [EPR5150(2)]	Abcam	Cat# ab128874, RRID:AB_11145462
Anti-β-catenin (H-102)	Santa Cruz Biotechnology	Cat# sc-7199, RRID:AB_634603
Anti-c-Myc (D3N8F)	Cell Signaling	Cat# 13987, RRID:AB_2631168
Anti-E2F1	Cell Signaling	Cat# 3742, RRID:AB_2096936
Anti-E2F2 [EPR8622]	Abcam	ab138515
Anti-E2F-3 (PG30)	Santa Cruz Biotechnology	Cat# sc-56665, RRID:AB_1122397
Anti-HuR (3A2)	Santa Cruz Biotechnology	Cat# sc-5261, RRID:AB_627770
Anti-GAPDH	Abcam	Cat# ab8245, RRID:AB_2107448
Anti-Phospho-Rb (Ser780)	Cell Signaling	Cat# 9307, RRID:AB_330015
Anti-Ki67	Abcam	Cat# ab66155, RRID:AB_1140752
Anti-Cleaved Caspase-3 (Asp175)	Cell Signaling	Cat# 9661, RRID:AB_2341188
Anti-Bromodeoxyuridine	Roche	Cat# 11170376001, RRID:AB_514483
Anti-YAP1	Thermo Fisher Scientific	Cat# PA1-46189, RRID:AB_2219137
Anti-TAZ (1F1)	Santa Cruz Biotechnology	Cat# sc-293183
Anti-TEF-1 (H-4) (Tead 1)	Santa Cruz Biotechnology	Cat# sc-376113, RRID:AB_10988229
Anti-Tead 2	Thermo Fisher Scientific	Cat# PA5-40316, RRID:AB_2607746
Anti-TEF-3 (B-5) (Tead 4)	Santa Cruz Biotechnology	Cat# sc-390578
Anti-Cyclin D1 (A-12)	Santa Cruz Biotechnology	Cat# sc-8396, RRID:AB_627344
Anti-Cyclin D2 (D52F9)	Cell Signaling Technology	Cat# 3741, RRID:AB_2070685
Anti-Cyclin E (E-4)	Santa Cruz Biotechnology	Cat# sc-377100
Anti-CDK2 (78B2)	Cell Signaling Technology	Cat# 2546, RRID:AB_2276129
Anti-CDK4	Cell Signaling Technology	Cat# 12790, RRID:AB_2631166
ANTI-CDK6 (DCS83)	Cell Signaling Technology	Cat# 3136, RRID:AB_2229289
Anti-p21 Waf1/Cip1 (12D1)	Cell Signaling Technology	Cat# 2947, RRID:AB_823586
Anti-p27 Kip1 (D69C12) XP	Cell Signaling Technology	Cat# 3686, RRID:AB_2077850
Anti-Phospho-Rb (Ser780)	Cell Signaling Technology	Cat# 9307, RRID:AB_330015
Anti-Rb (IF8)	Santa Cruz Biotechnology	Cat# sc-102, RRID:AB_628209
Anti-Bcl-9(B-4)	Santa Cruz Biotechnology	Cat# sc-398131
Anti-Sox9 antibody	Millipore	Cat# AB5535, RRID:AB_2239761
Anti-BRD2 (ChIP)	Bethyl Laboratories	A302-583A, RRID:AB_2034829
Anti-BRD3 (ChIP)	Bethyl Laboratories	A302-367A, RRID:AB_1907250
Anti-BRD4 (ChIP)	Bethyl Laboratories	A301-985A50, RRID:AB_2631449
Anti-Histone H3 (mono methyl K4)	Abcam	Cat# ab8895, RRID:AB_306847
Anti-Histone H3 (acetyl K27)	Abcam	Cat# ab4729, RRID:AB_2118291

Recombinant DNA	Abbreviation in manuscript	Source (Reference)
pLKO.puro.empty	sh Ctrl	SIGMA-ALDRICH (SHC001)
pLKO.puro.shHuR#1	sh HuR#1 (sh H#1)	SIGMA-ALDRICH (TRCN0000276186)
pLKO.puro.shHuR#2	sh HuR#2	SIGMA-ALDRICH (TRCN0000276129)
pLKO.puro.shβ-cat#1 **(a)**	sh β-Cat#1	ADDGENE (ref 18803)
pLKO.puro.shβ-cat#2 (**b**)	sh β-Cat#2	ADDGENE (ref 19761)
pLKO.puro.shBRD2#1	sh BRD2#1	GE Healthcare (TRCN0000006308)
pLKO.puro.shBRD2#2	sh BRD2#2	GE Healthcare (TRCN0000006310)
TRIPZ -non-silencing shRNAmir-Ctrl	TRIPZ-sh *i*Control	DHARMACON (RHS4743)
TRIPZ-shHuR#3	TRIPZ-sh *i*HuR	DHARMACON (V3THS_331824)
pcw107.puro.empty **(c)**	pcw107–EV	ADDGENE (ref 62511)
pcw107.puroBETA-CATENIN (S33A, S37A, T41A, S45A) **(d)**	pcw107–β-Cat (4A)	ADDGENE (ref 64612)
pLKO.neo.empty **(e)**	sh Control (shC)	ADDGENE (ref 13425)
pLKO.neo.shHuR	sh HuR#3 (shH#3)	SIGMA-ALDRICH (TRCN0000276186)
pCDH-EF1-FHC **(f)**	pCDH-EV	ADDGENE (ref 64874)
pCDH-puro-cMyc **(g)**	pCDH-c-MYC	ADDGENE (ref 46970)
plenti6.2 EGFP-SOX9	pLenti6.2-EV	kindly provided by Vincent J Hearing, NCI
plenti6.2 EGFP	pLenti6.2-SOX9	kindly provided by Vincent J Hearing, NCI
pWPI-EV **(h)**	pWPI-EV	ADDGENE (ref 12254)
pWPI-E2F1 **(i)**	pWPI-E2F1	ADDGENE (ref 114296)
pWPI-E2F2 **(j)**	pWPI-E2F2	ADDGENE (ref 114297)
pWPI-E2F3 **(k)**	pWPI-E2F3	ADDGENE (ref 114298)
pCDH-empty	pCDH-EV	kindly provided by Ruben D. Carrasco, Harvard University
pCDH-BCL9	pCDH-BCL9	kindly provided by Ruben D. Carrasco, Harvard University
TRIPZ-HuR	TRIPZ-HuR	kindly provided by Samuel C. Dudley, Lillehei Heart Institute

ACKNOWLEDGEMENTS	REFERENCES
**(a)** pLKO.1 puro shRNA beta-catenin was a gift from Bob Weinberg (Addgene plasmid # 18803 ; http://n2t.net/addgene:18803 ; RRID:Addgene_18803)	(23)
**(b)** pLKO.1.sh.beta-catenin.1248 was a gift from William Hahn (Addgene plasmid # 19761 ; http://n2t.net/addgene:19761 ; RRID:Addgene_19761)	(24)
**(c)** pcw107 was a gift from John Doench & David Sabatini (Addgene plasmid # 62511 ; http://n2t.net/addgene:62511 ; RRID:Addgene_62511)	(25)
**(d)** beta-catenin (S33A, S37A, T41A, S45A)-pcw107 was a gift from David Sabatini & Kris Wood (Addgene plasmid # 64612 ; http://n2t.net/addgene:64612 ; RRID:Addgene_64612)	(25)
**(e)** pLKO.1 neo was a gift from Sheila Stewart (Addgene plasmid # 13425 ; http://n2t.net/addgene:13425 ; RRID:Addgene_13425)	–
**(f)** pCDH-EF1-FHC was a gift from Richard Wood (Addgene plasmid # 64874 ; http://n2t.net/addgene:64874 ; RRID:Addgene_64874)	(26)
**(g)** pCDH-puro-cMyc was a gift from Jialiang Wang (Addgene plasmid # 46970 ; http://n2t.net/addgene:46970 ; RRID:Addgene_46970)	(27)
**(h)** pWPI was a gift from Didier Trono (Addgene plasmid # 12254 ; http://n2t.net/addgene:12254 ; RRID:Addgene_12254)	–
**(i)** pWPI-E2F1 was a gift from Patrick Salmon (Addgene plasmid # 114296 ; http://n2t.net/addgene:114296 ; RRID:Addgene_114296)	–
**(j)** pWPI-E2F2 was a gift from Patrick Salmon (Addgene plasmid # 114297 ; http://n2t.net/addgene:114297 ; RRID:Addgene_114297)	–
**(k)** pWPI-E2F3 was a gift from Patrick Salmon (Addgene plasmid # 114298 ; http://n2t.net/addgene:114298 ; RRID:Addgene_114298)	–

SUPPLEMENTARY REFERENCES

1. Pytel P, et al. Neoplasms with schwannian differentiation express transcription factors known to regulate normal schwann cell development. *Int J Surg Pathol.* 2010;18(6):449-457.
2. Keene JD, Komisarow JM, Friedersdorf MB. RIP-Chip: the isolation and identification of mRNAs, microRNAs and protein components of ribonucleoprotein complexes from cell extracts. *Nat Protoc.* 2006;1(1):302-307.
3. Jayaseelan S, Doyle F, Tenenbaum SA. Profiling post-transcriptionally networked mRNA subsets using RIP-Chip and RIP-Seq. *Methods.* 2014;67(1):13-19.
4. Martin M. Cutadapt removes adapter sequences from high-throughput sequencing reads. *EMBnetJournal.* 2011;17(1):10–12.
5. Dobin A, et al. STAR: ultrafast universal RNA-seq aligner. *Bioinformatics.* 2013;29(1):15-21.
6. Li B, Dewey CN. RSEM: accurate transcript quantification from RNA-Seq data with or without a reference genome. *BMC Bioinformatics.* 2011;12:323.
7. Ritchie ME, et al. limma powers differential expression analyses for RNA-sequencing and microarray studies. *Nucleic Acids Res.* 2015;43(7):e47.
8. Fontanals-Cirera B, et al. Harnessing BET Inhibitor Sensitivity Reveals AMIGO2 as a Melanoma Survival Gene. *Mol Cell.* 2017;68(4):731-744 e739.
9. Zhang Y, et al. Model-based analysis of ChIP-Seq (MACS). *Genome Biol.* 2008;9(9):R137.
10. Ascension AM, Arrospide-Elgarresta M, Izeta A, Arauzo-Bravo MJ. NaviSE: superenhancer navigator integrating epigenomics signal algebra. *BMC Bioinformatics.* 2017;18(1):296.
11. Subramanian A, et al. Gene set enrichment analysis: a knowledge-based approach for interpreting genome-wide expression profiles. *Proc Natl Acad Sci U S A.* 2005;102(43):15545-15550.
12. Chen J, Bardes EE, Aronow BJ, Jegga AG. ToppGene Suite for gene list enrichment analysis and candidate gene prioritization. *Nucleic Acids Res.* 2009;37(Web Server issue):W305-311.
13. Jessen WJ, et al. MEK inhibition exhibits efficacy in human and mouse neurofibromatosis tumors. *J Clin Invest.* 2013;123(1):340-347.
14. Miller SJ, et al. Integrative genomic analyses of neurofibromatosis tumours identify SOX9 as a biomarker and survival gene. *EMBO Mol Med.* 2009;1(4):236-248.
15. Wu LMN, et al. Programming of Schwann Cells by Lats1/2-TAZ/YAP Signaling Drives Malignant Peripheral Nerve Sheath Tumorigenesis. *Cancer Cell.* 2018;33(2):292-308 e297.
16. Tremblay AM, et al. The Hippo transducer YAP1 transforms activated satellite cells and is a potent effector of embryonal rhabdomyosarcoma formation. *Cancer Cell.* 2014;26(2):273-287.
17. De Raedt T, et al. PRC2 loss amplifies Ras-driven transcription and confers sensitivity to BRD4-based therapies. *Nature.* 2014;514(7521):247-251.
18. Fishilevich S, et al. GeneHancer: genome-wide integration of enhancers and target genes in GeneCards. *Database (Oxford).* 2017;2017.

19. Nikolsky Y, Ekins S, Nikolskaya T, Bugrim A. A novel method for generation of signature networks as biomarkers from complex high throughput data. *Toxicol Lett.* 2005;158(1):20-29.
20. Crespo I, Krishna A, Le Bechec A, del Sol A. Predicting missing expression values in gene regulatory networks using a discrete logic modeling optimization guided by network stable states. *Nucleic Acids Res.* 2013;41(1):e8.
21. Shannon P, et al. Cytoscape: a software environment for integrated models of biomolecular interaction networks. *Genome Res.* 2003;13(11):2498-2504.
22. Zhou A, et al. RNA Binding Protein, HuR, Regulates SCN5A Expression Through Stabilizing MEF2C transcription factor mRNA. *J Am Heart Assoc.* 2018;7(9).
23. Onder TT, et al. Loss of E-cadherin promotes metastasis via multiple downstream transcriptional pathways. *Cancer Res.* 2008;68(10):3645-3654.
24. Firestein R, et al. CDK8 is a colorectal cancer oncogene that regulates beta-catenin activity. *Nature.* 2008;455(7212):547-551.
25. Martz CA, et al. Systematic identification of signaling pathways with potential to confer anticancer drug resistance. *Sci Signal.* 2014;7(357):ra121.
26. Yousefzadeh MJ, et al. Mechanism of suppression of chromosomal instability by DNA polymerase POLQ. *PLoS Genet.* 2014;10(10):e1004654.
27. Cheng Z, et al. Inhibition of BET bromodomain targets genetically diverse glioblastoma. *Clin Cancer Res.* 2013;19(7):1748-1759.

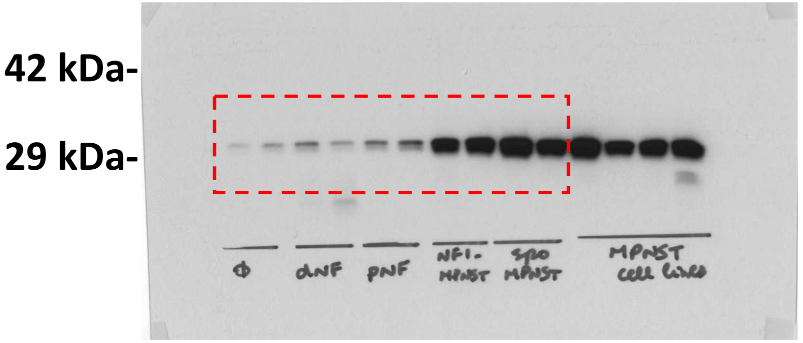

HuR

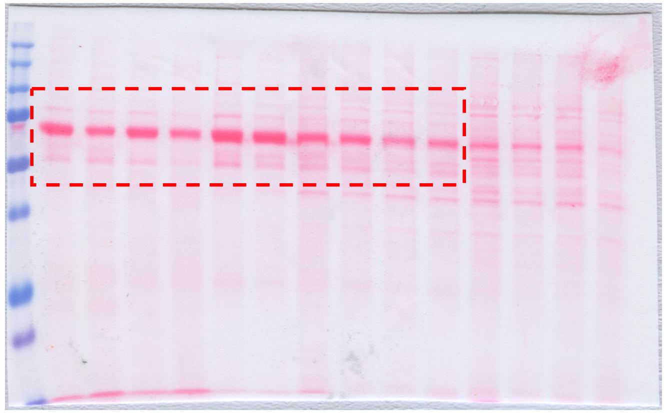

Ponceau

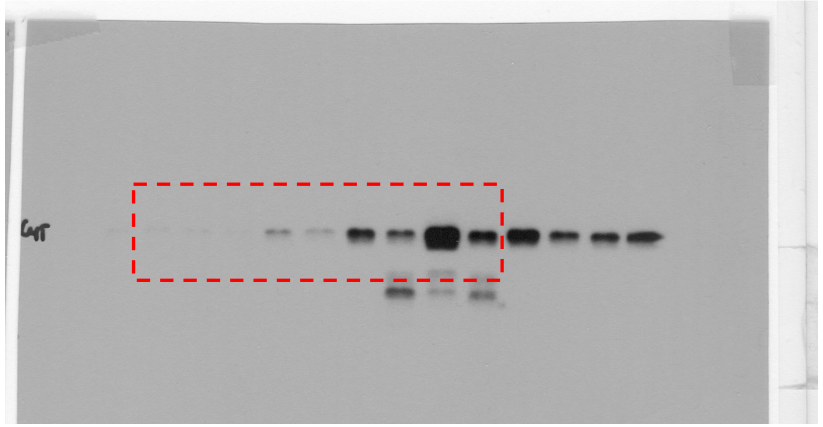

Cytoplasmic HuR

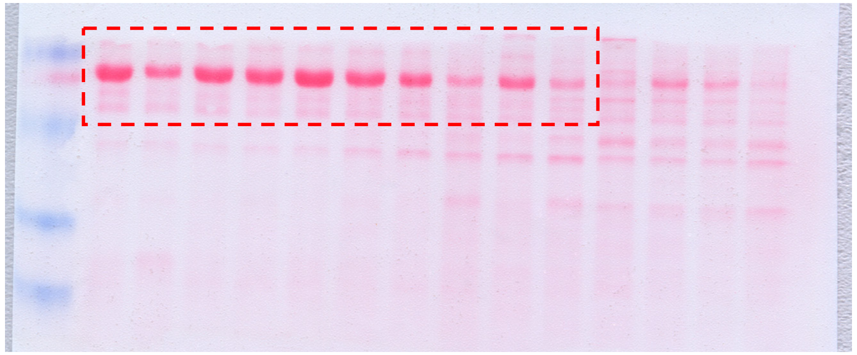

Ponceau

Full unedited gel Figure 1D

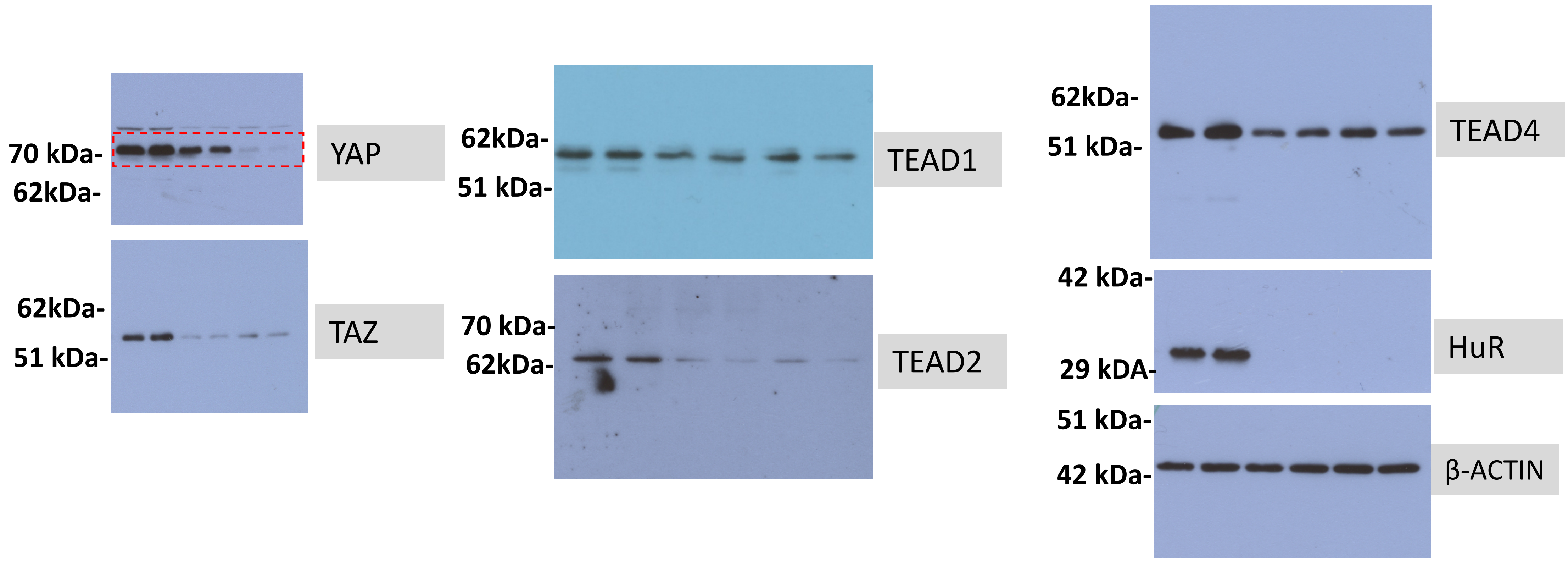

Full unedited gel Figure 7D

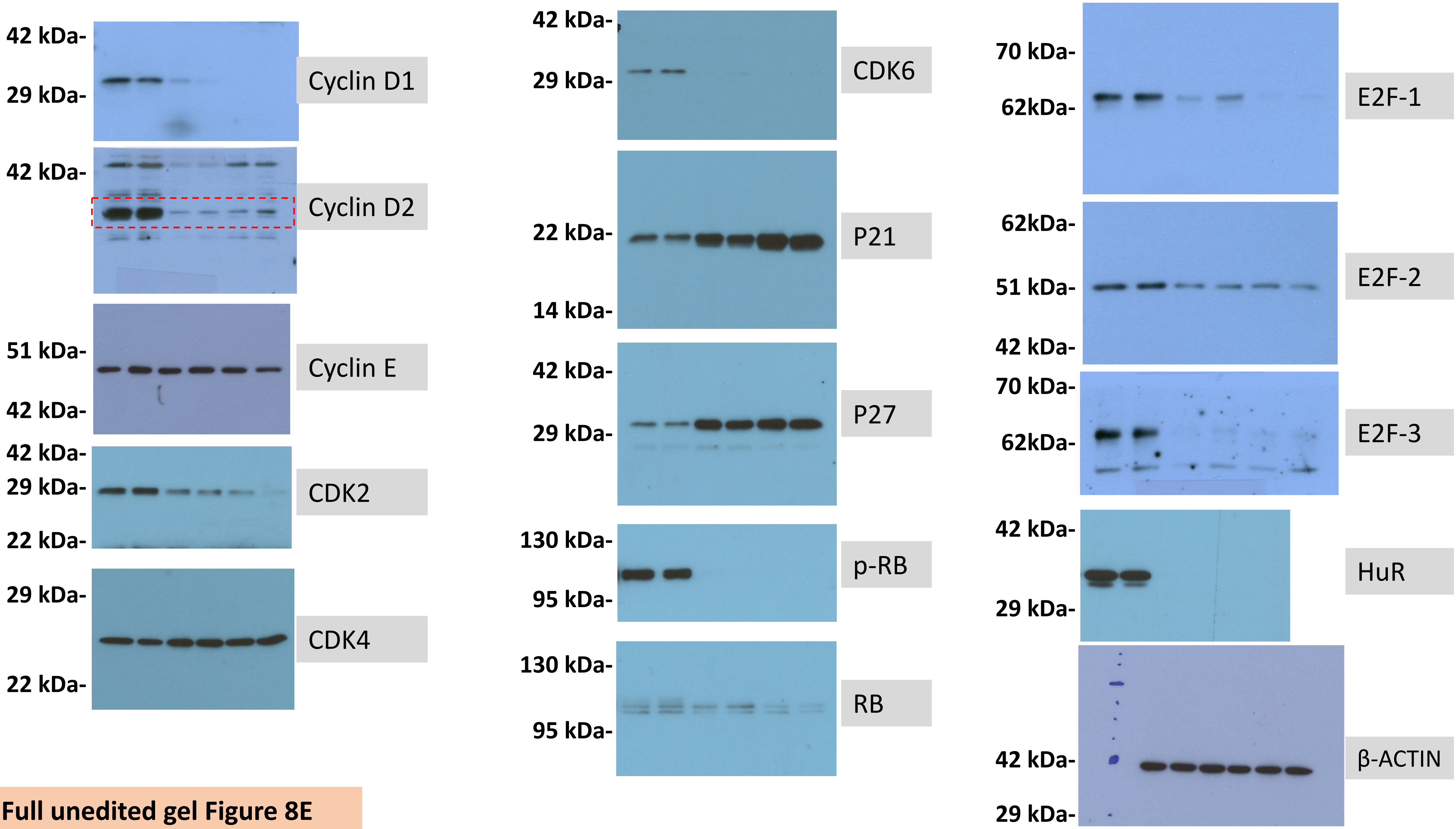

Full unedited gel Figure 8E

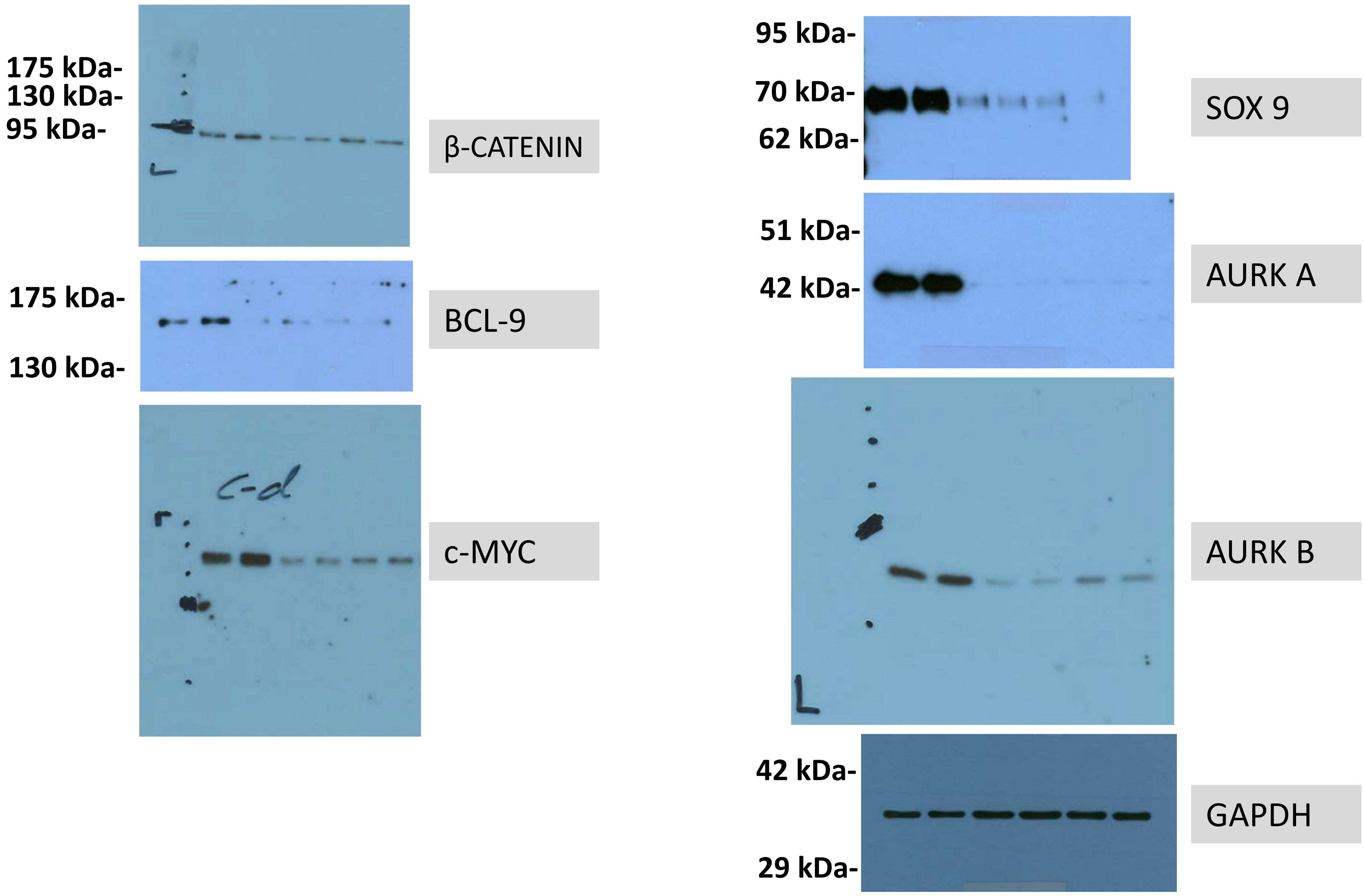

Full unedited gel Figure 9D

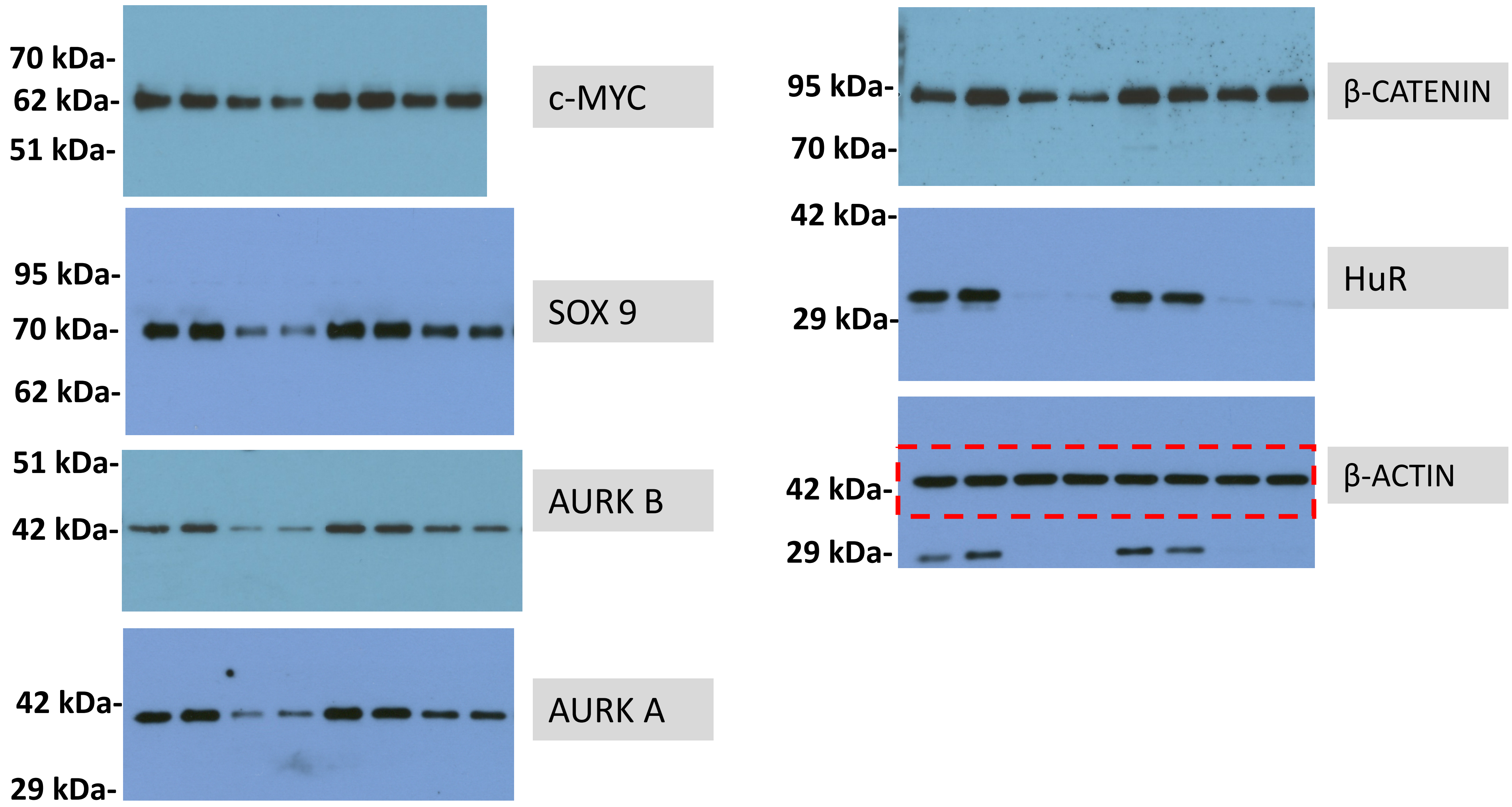

Full unedited gel Figure 9E

95 kDa-

42 kDa-

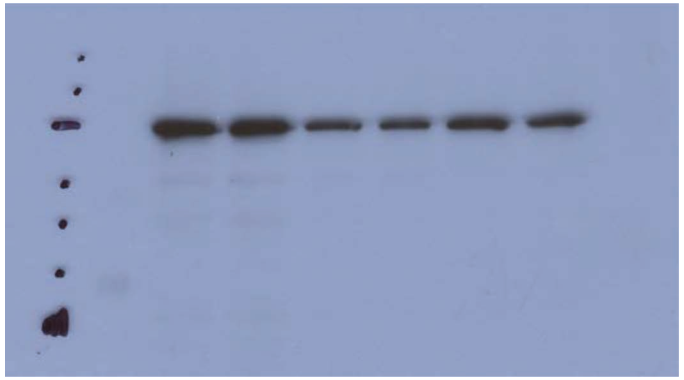

BRD2

95 kDa-

42 kDa-

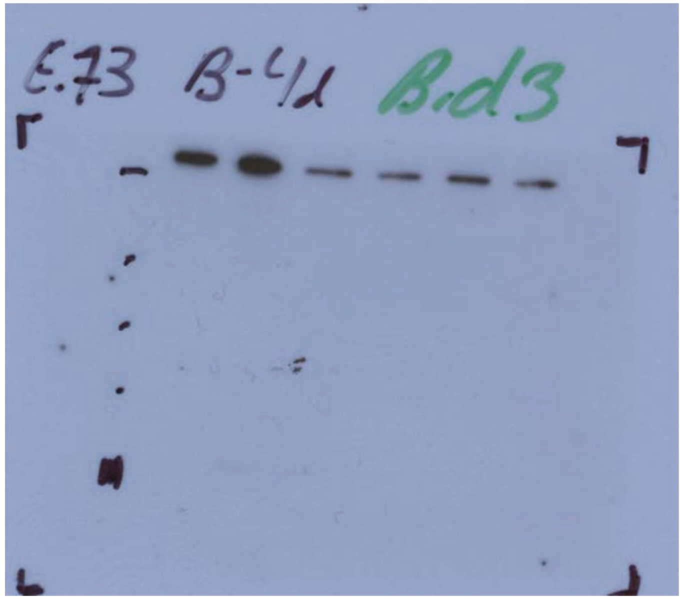

BRD3

95 kDa-

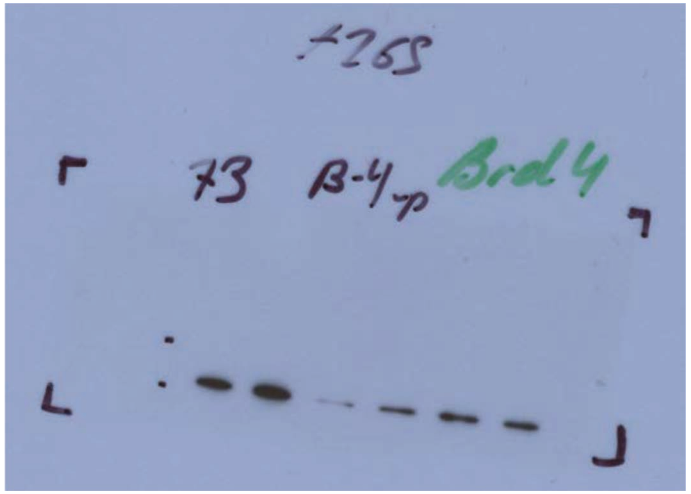

BRD4

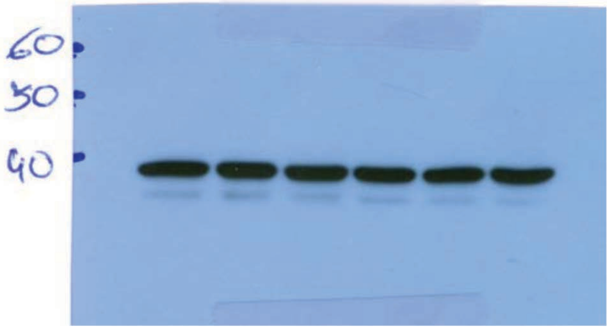

β-ACTIN

Full unedited gel Figure 10C

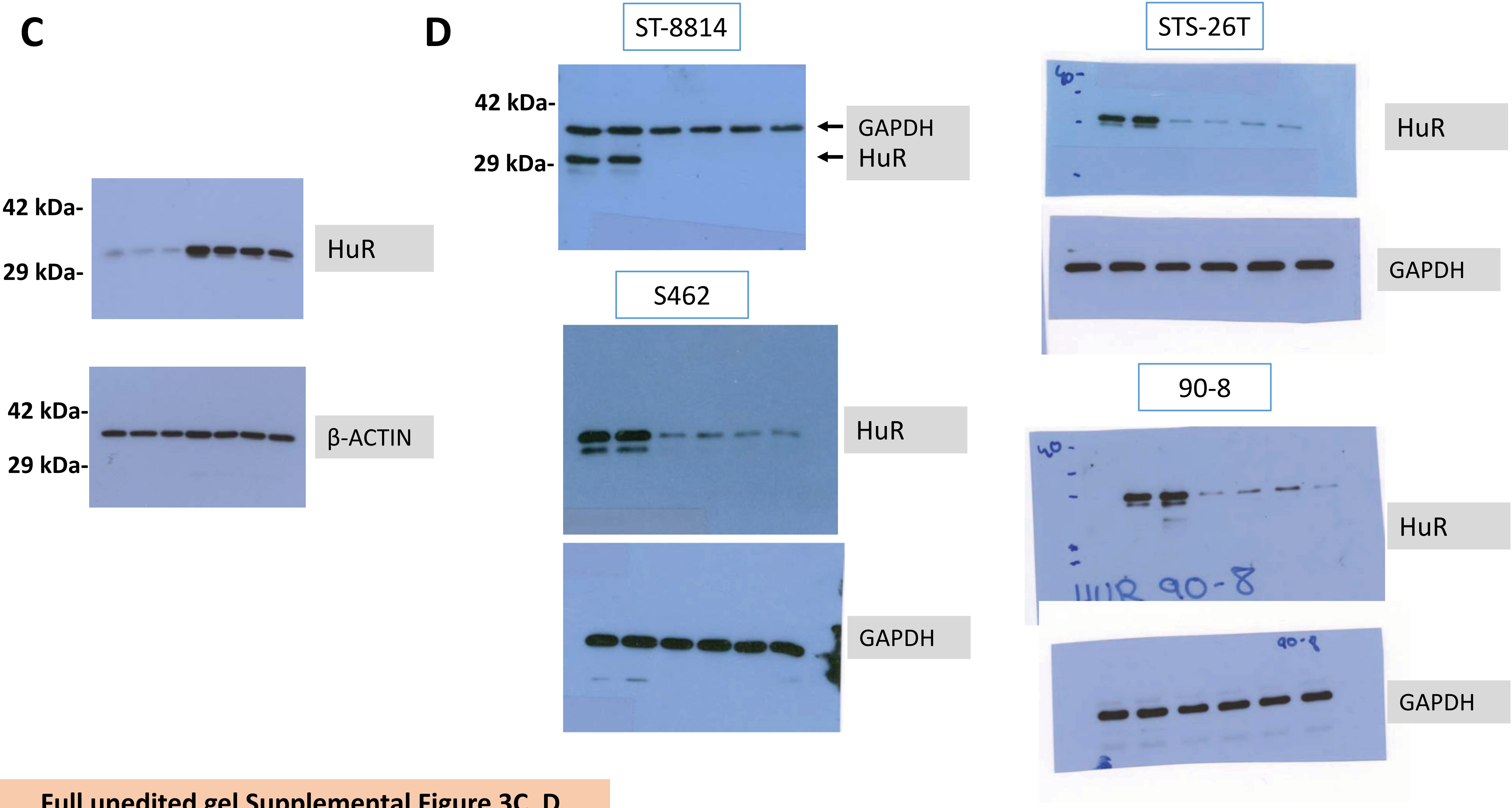

Full unedited gel Supplemental Figure 3C, D

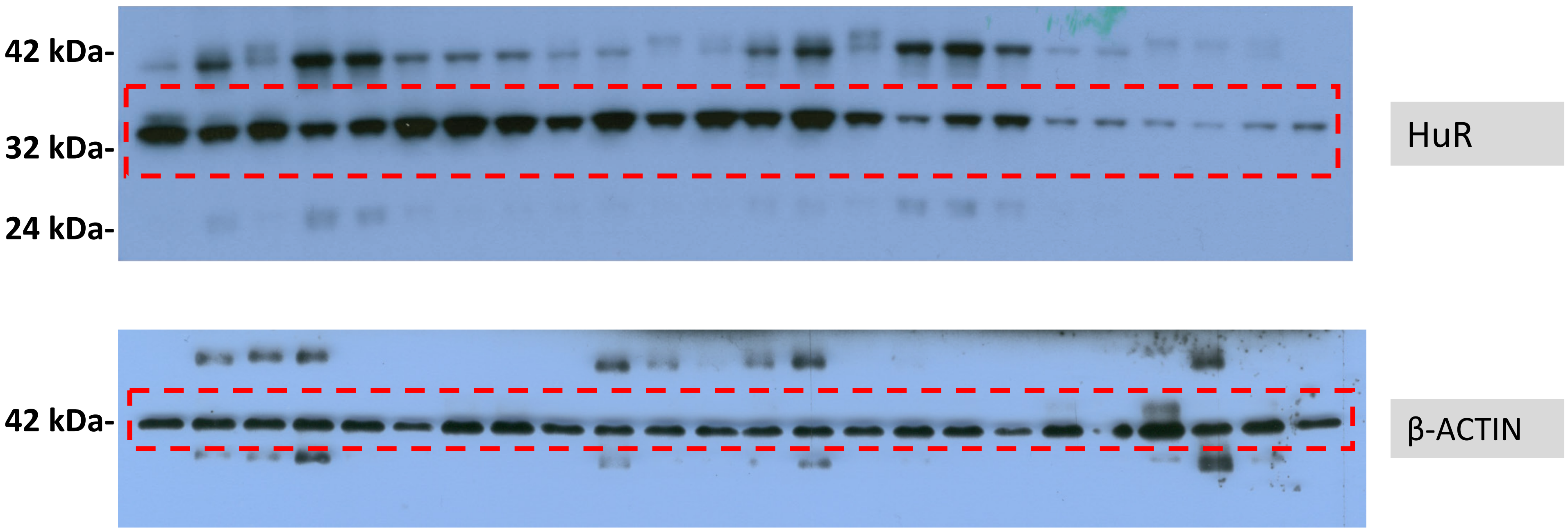

Full unedited gel Supplemental Figure 5B

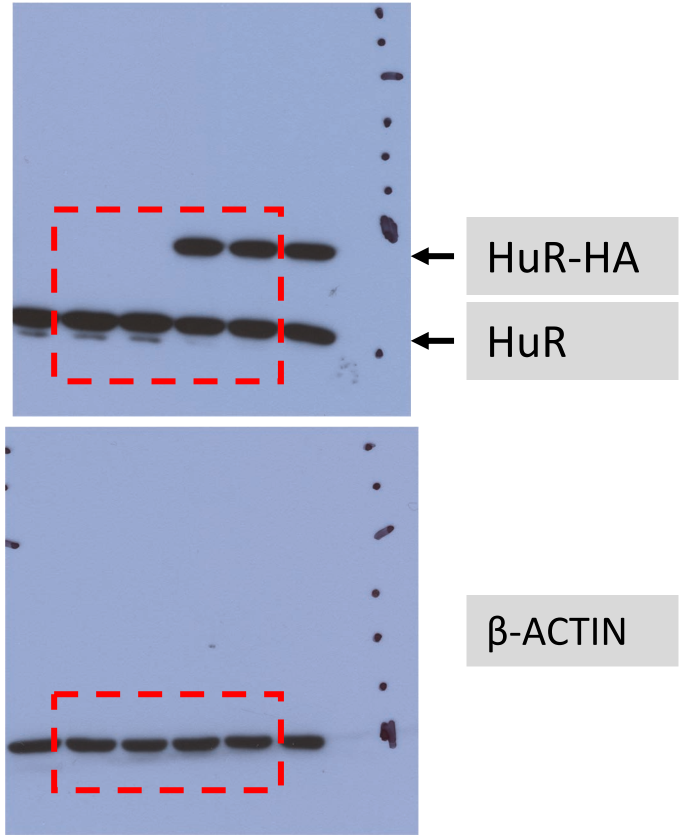

Full unedited gel Supplemental Figure 7A

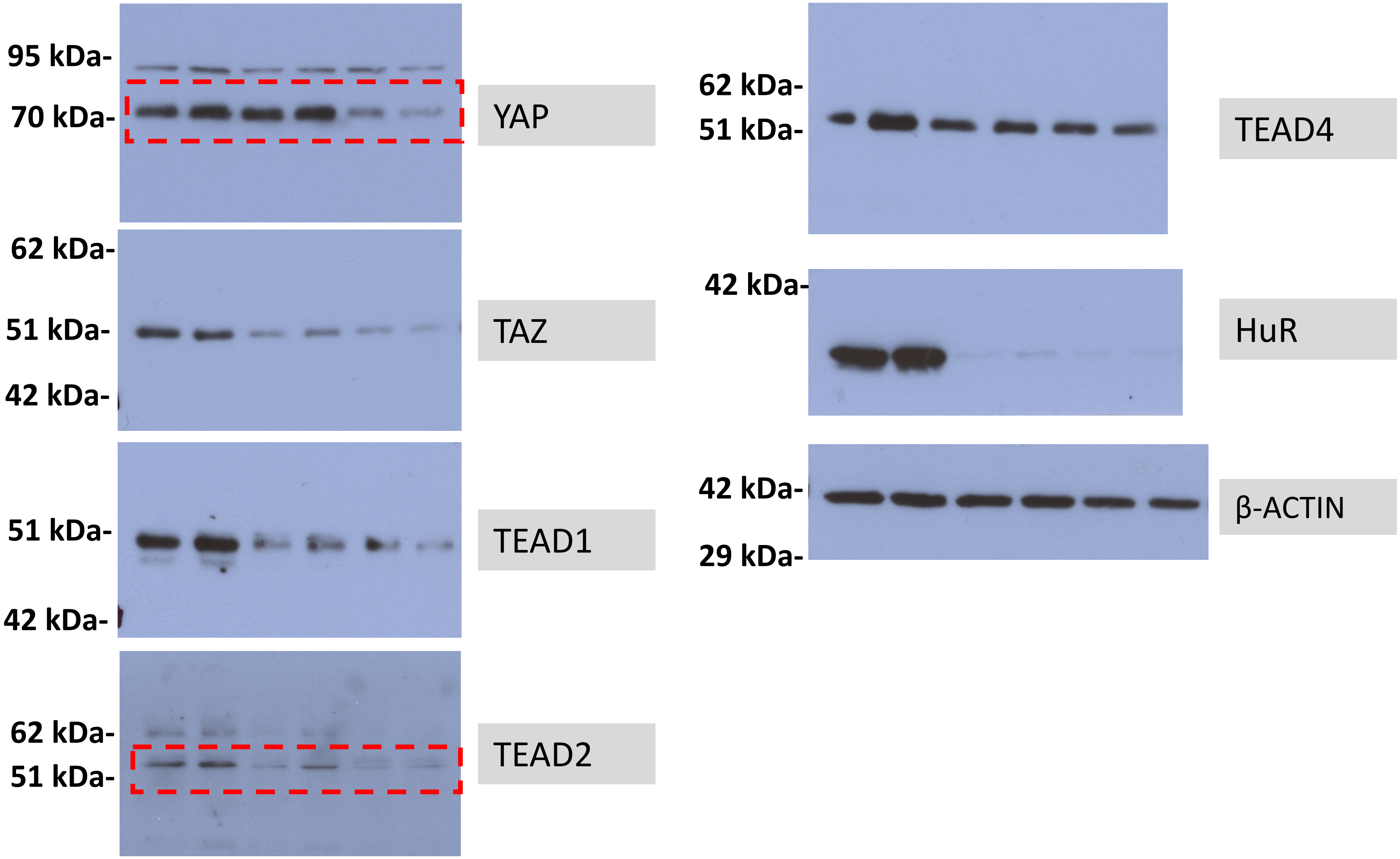

Full unedited gel Supplemental Figure 8B

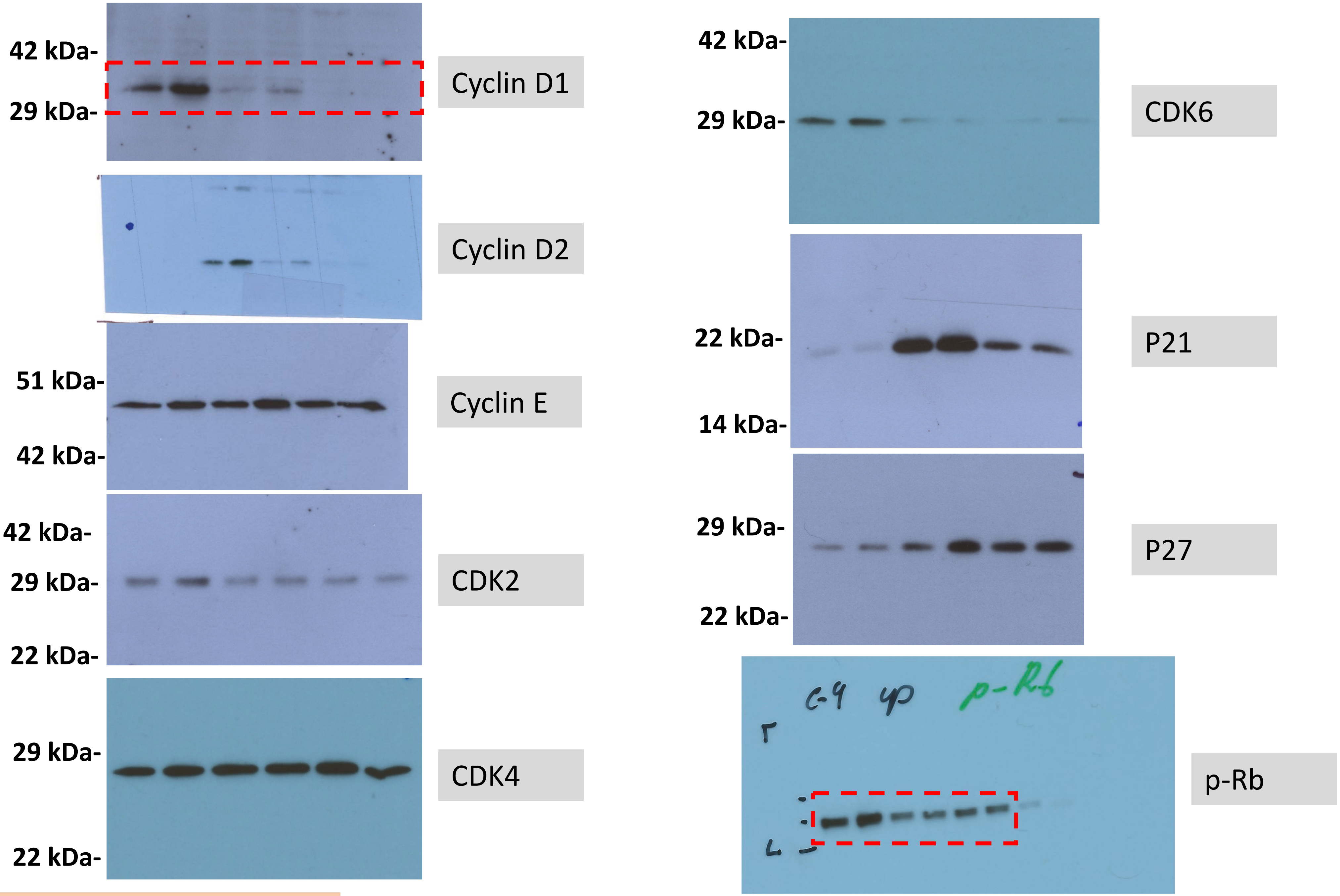

Full unedited gel Supplemental Figure 9D

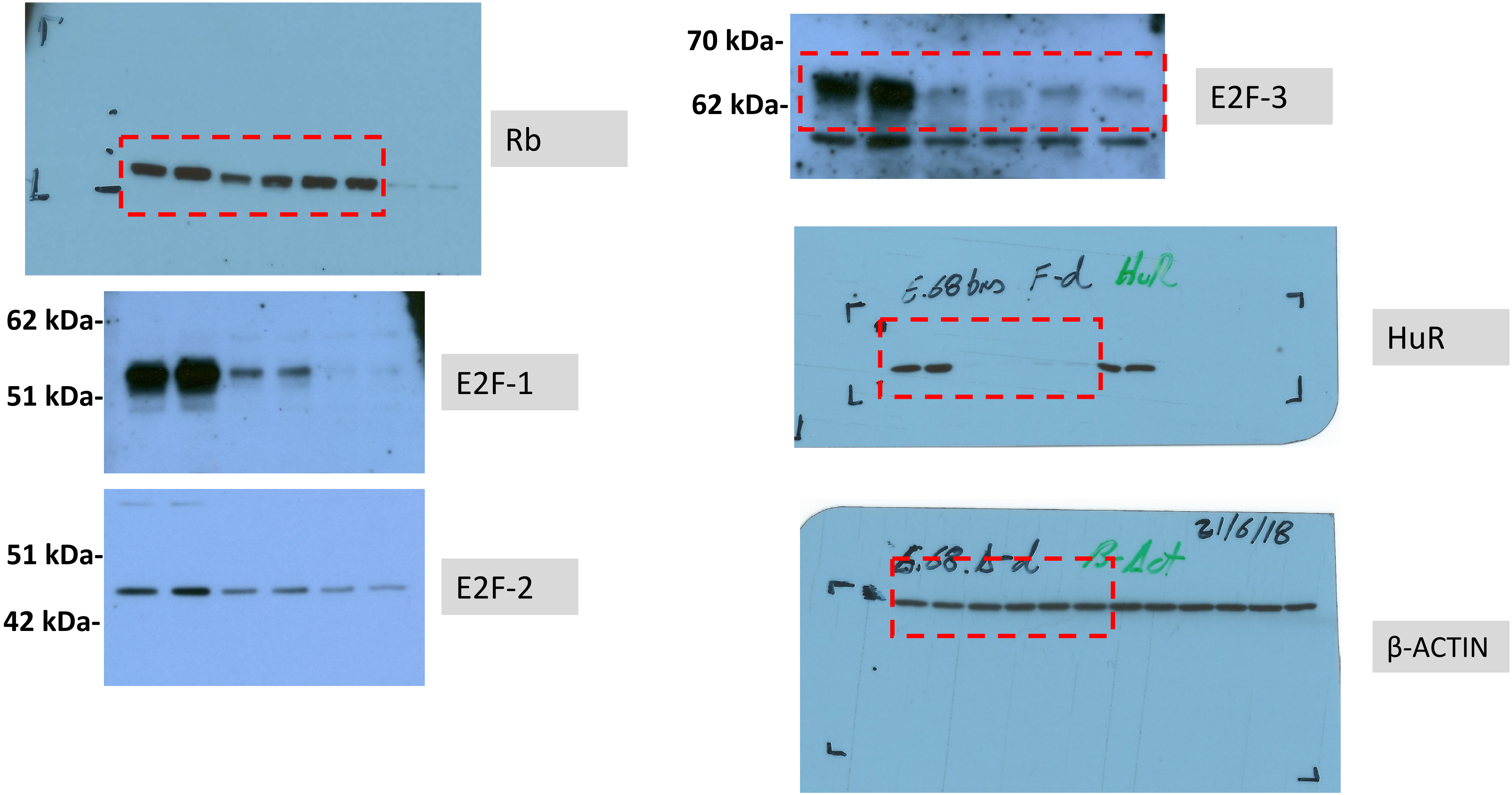

Full unedited gel Supplemental Figure 9D

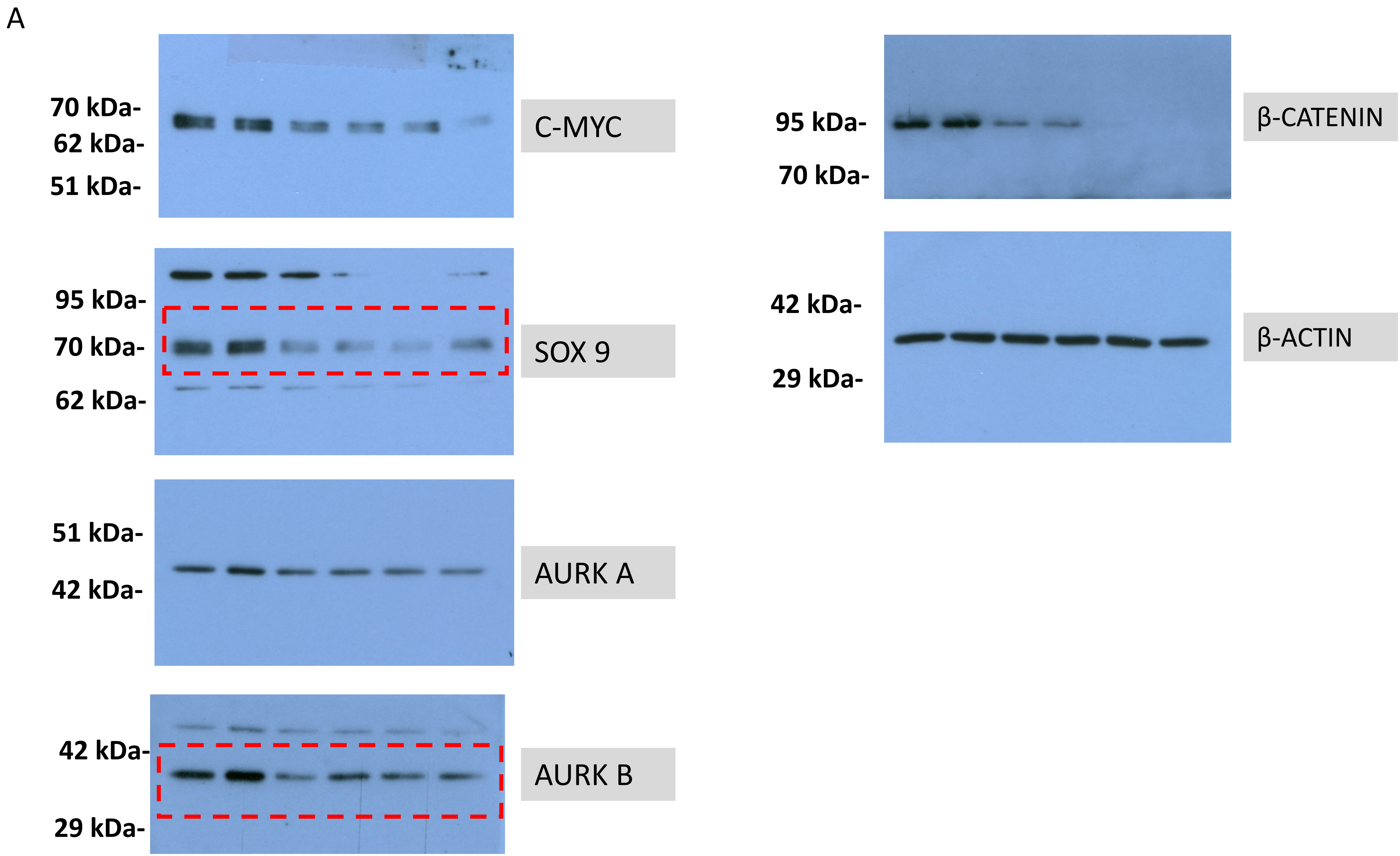

Full unedited gel Supplemental Figure 10A

B

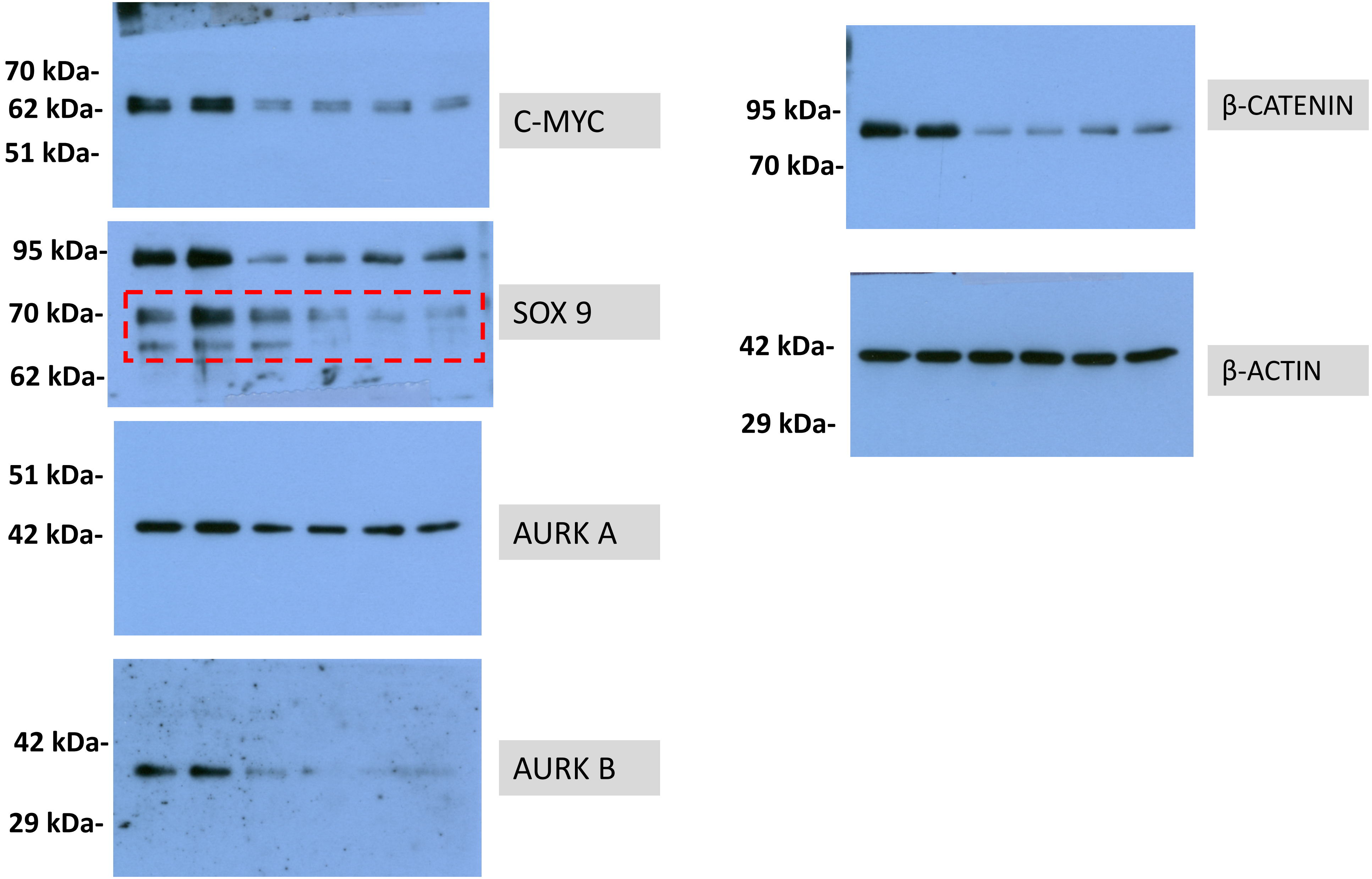

Full unedited gel Supplemental Figure 10B

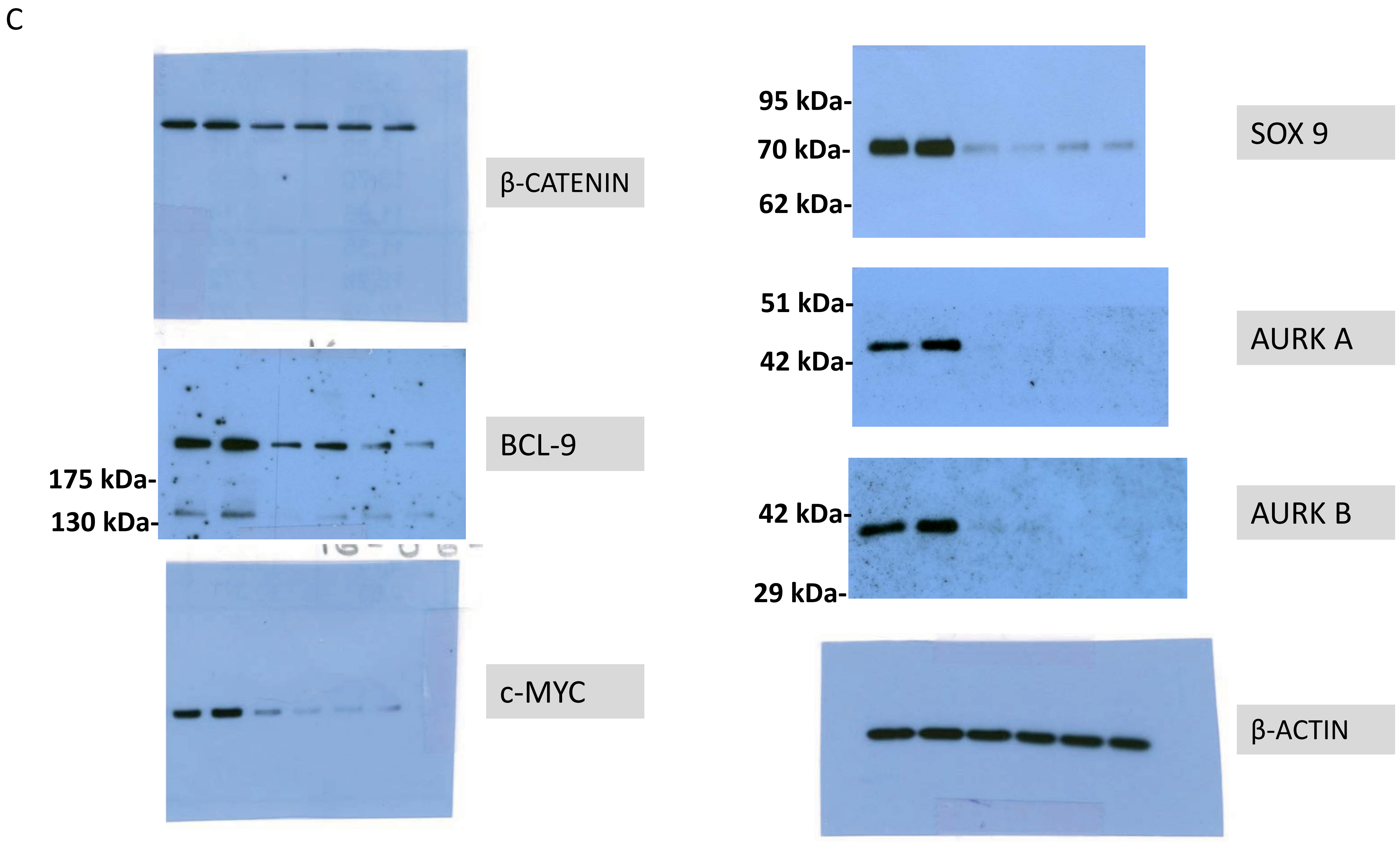

Full unedited gel Supplemental Figure 10C

D

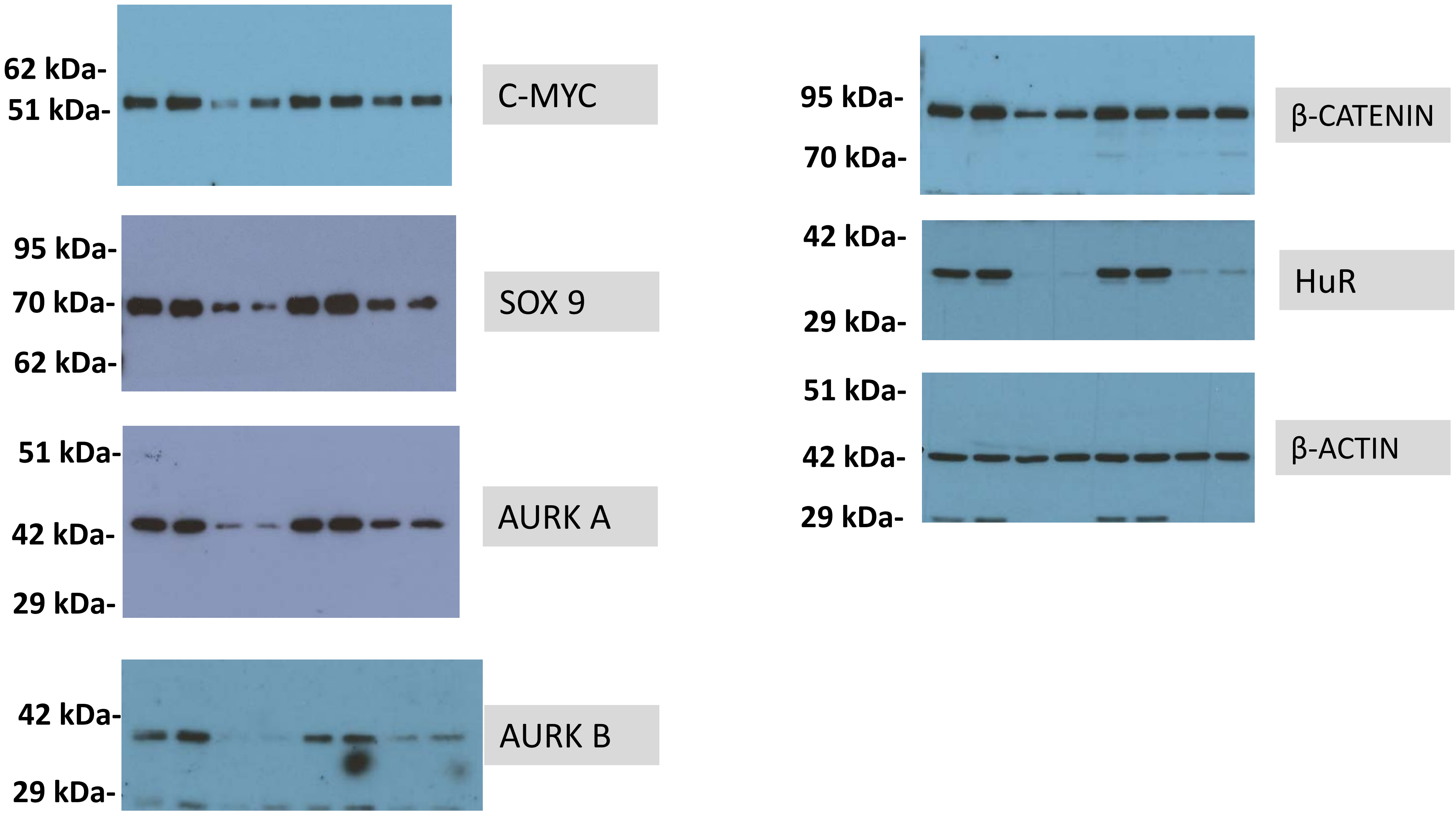

Full unedited gel Supplemental Figure 10D

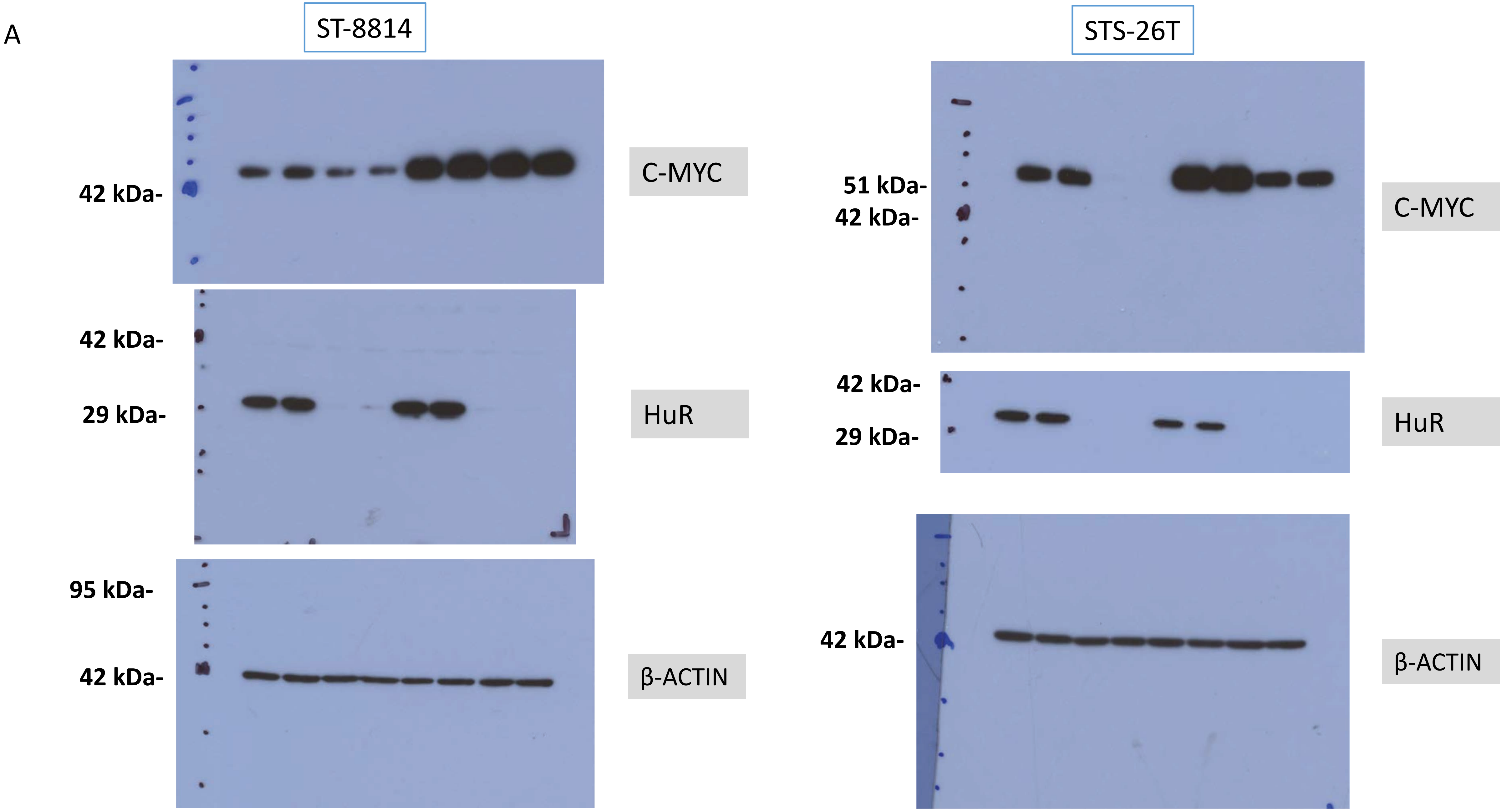

Full unedited gel Supplemental Figure 11A

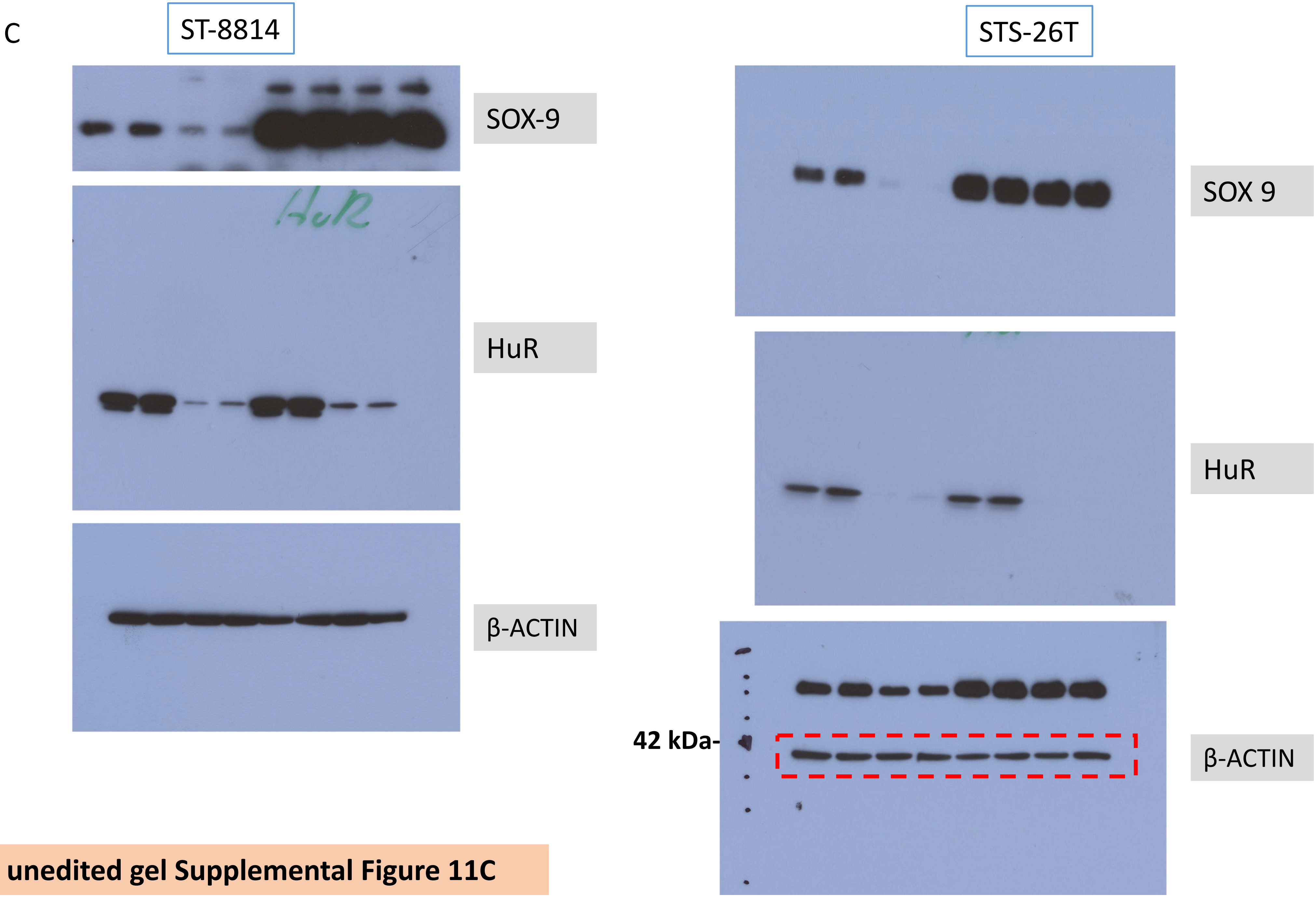

Full unedited gel Supplemental Figure 11C

A

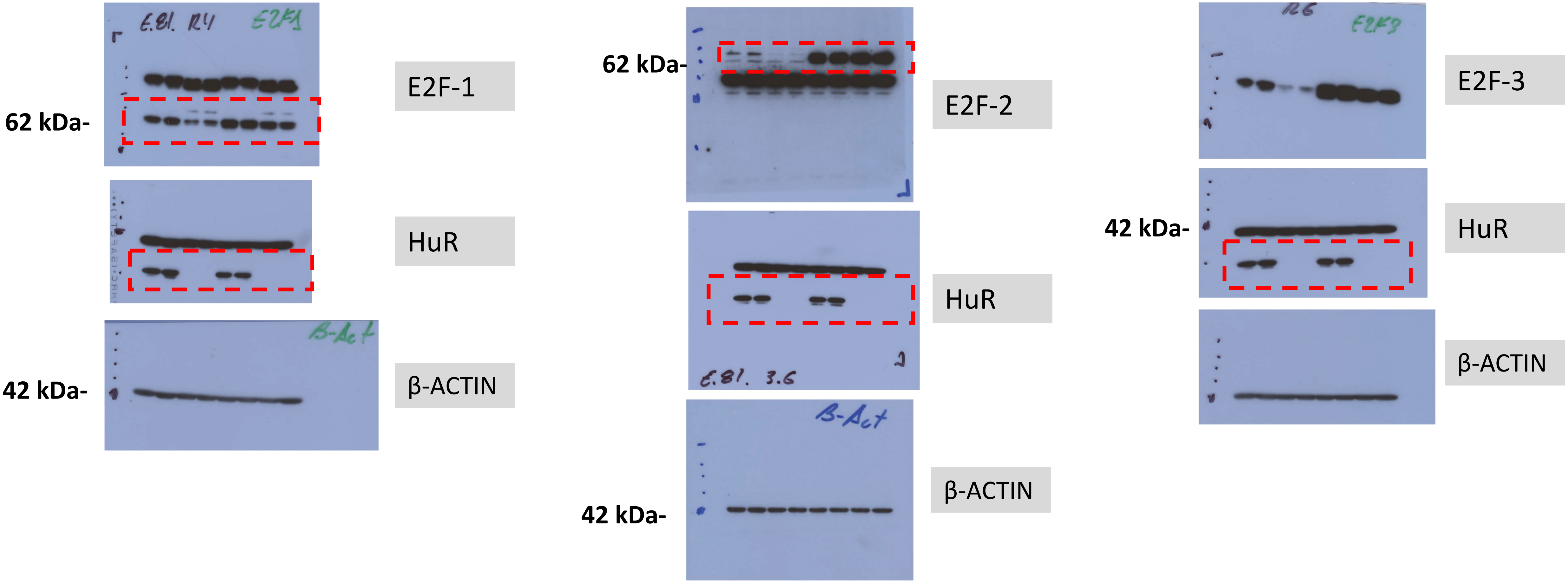

Full unedited gel Supplemental Figure 12A

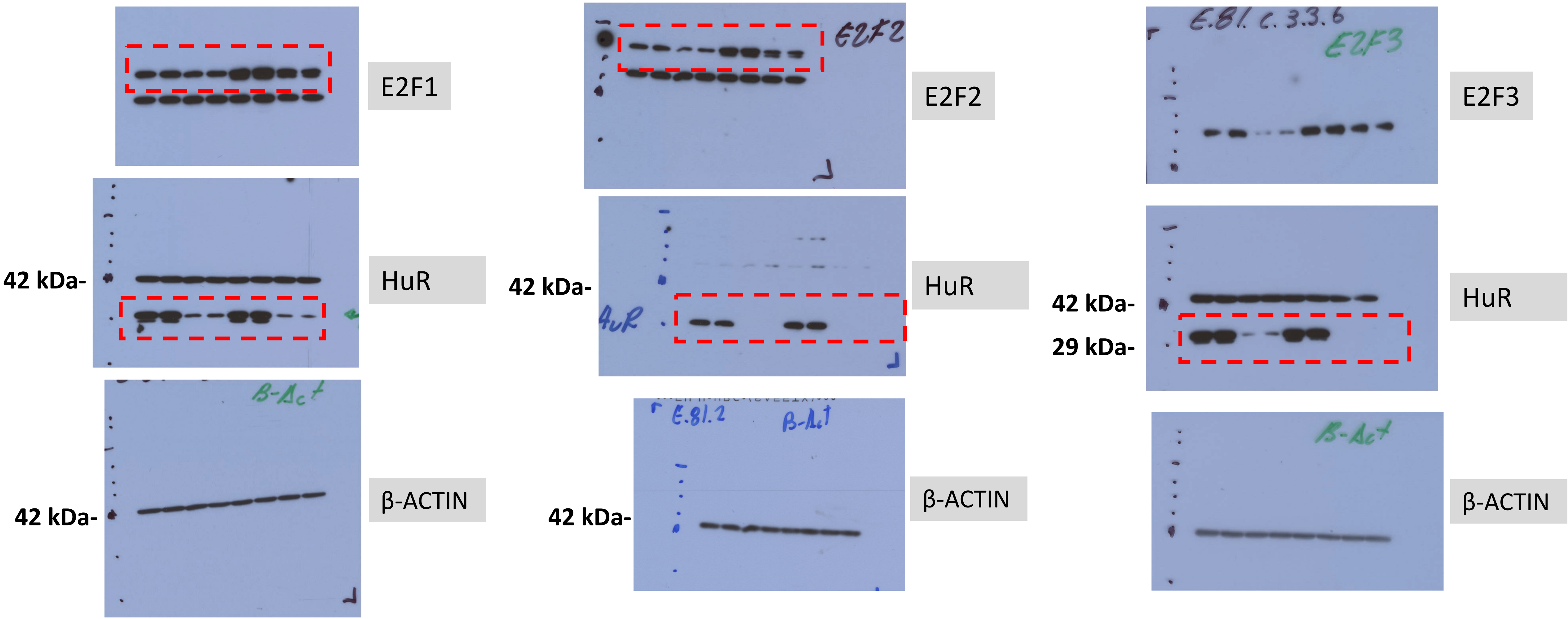

Full unedited gel Figure Supplemental 12C

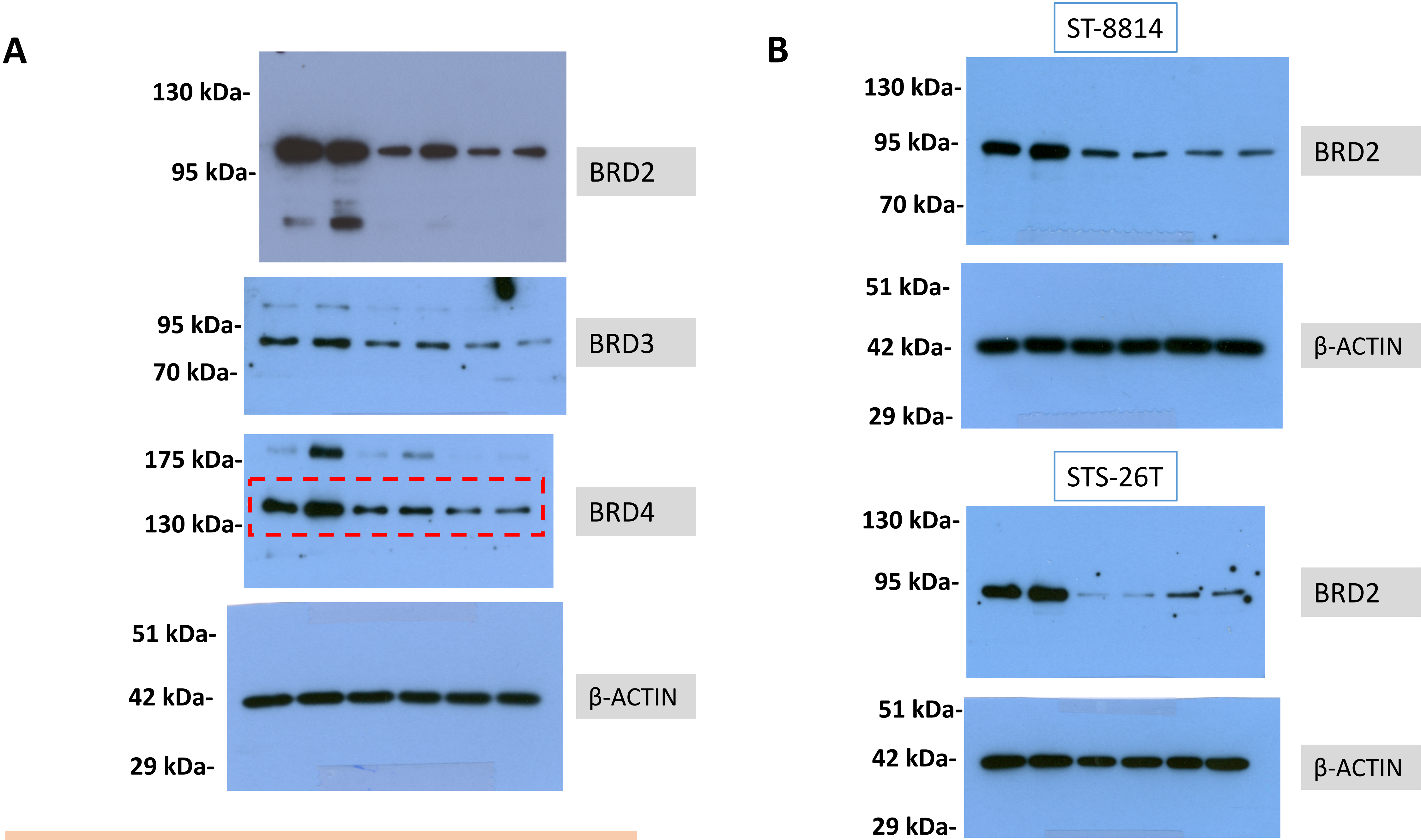

Full unedited gel Supplemental Figure 13A, B
